# Synthesis and Cheminformatics-Directed Antibacterial Evaluation of Echinosulfonic Acid-Inspired Bis-Indole Alkaloids

**DOI:** 10.3390/molecules29122806

**Published:** 2024-06-12

**Authors:** Darren C. Holland, Joshua B. Hayton, Milton J. Kiefel, Anthony R. Carroll

**Affiliations:** 1 School of Environment and Science, Griffith University, Southport, QLD 4222, Australia; j.hayton@griffith.edu.au (J.B.H.); m.kiefel@griffith.edu.au (M.J.K.); 2 Griffith Institute for Drug Discovery, Griffith University, Nathan, QLD 4111, Australia; 3 Institute for Glycomics, Griffith University, Southport, QLD 4221, Australia

**Keywords:** bis-indole alkaloids, marine natural products, cheminformatics, structure revision, antibacterial

## Abstract

Synthetic efforts toward complex natural product (NP) scaffolds are useful ones, particularly those aimed at expanding their bioactive chemical space. Here, we utilised an orthogonal cheminformatics-based approach to predict the potential biological activities for a series of synthetic bis-indole alkaloids inspired by elusive sponge-derived NPs, echinosulfone A (**1**) and echinosulfonic acids A–D (**2**–**5**). Our work includes the first synthesis of desulfato-echinosulfonic acid C, an α-hydroxy bis(3′-indolyl) alkaloid (**17**), and its full NMR characterisation. This synthesis provides corroborating evidence for the structure revision of echinosulfonic acids A-C. Additionally, we demonstrate a robust synthetic strategy toward a diverse range of α-methine bis(3′-indolyl) acids and acetates (**11**–**16**) without the need for silica-based purification in either one or two steps. By integrating our synthetic library of bis-indoles with bioactivity data for 2048 marine indole alkaloids (reported up to the end of 2021), we analyzed their overlap with marine natural product chemical diversity. Notably, the C-6 dibrominated α-hydroxy bis(3′-indolyl) and α-methine bis(3′-indolyl) analogues (**11**, **14**, and **17**) were found to contain significant overlap with antibacterial C-6 dibrominated marine bis-indoles, guiding our biological evaluation. Validating the results of our cheminformatics analyses, the dibrominated α-methine bis(3′-indolyl) alkaloids (**11**, **12**, **14**, and **15**) were found to exhibit antibacterial activities against methicillin-sensitive and -resistant *Staphylococcus aureus*. Further, while investigating other synthetic approaches toward bis-indole alkaloids, 16 incorrectly assigned synthetic α-hydroxy bis(3′-indolyl) alkaloids were identified. After careful analysis of their reported NMR data, and comparison with those obtained for the synthetic bis-indoles reported herein, all of the structures have been revised to α-methine bis(3′-indolyl) alkaloids.

## 1. Introduction

Marine indole alkaloids (MIAs) represent a diverse subclass of natural products (NPs) with increasing numbers continuing to be reported each year [1,2]. However, the majority of MIAs, and marine natural products (MNPs) more broadly, have been examined against a narrow breadth of disease and infection targets. In most cases, they are reported with potencies well below the threshold useful for further drug development [1,3]. To increase the likelihood of uncovering meaningful NP bioactivities, more thoughtful approaches toward their biological evaluations should be adopted. At the very least, more directed investigations of NP bioactivity will contribute to an expanded understanding of bioactive NP chemical space. Despite ongoing advancements in secondary metabolite omics methods, including metabolomics, genomics, proteomics, and transcriptomics, the translation of these data toward more sophisticated approaches for selecting biological targets for NP activity continues to lag behind [1].

**Figure 1:**
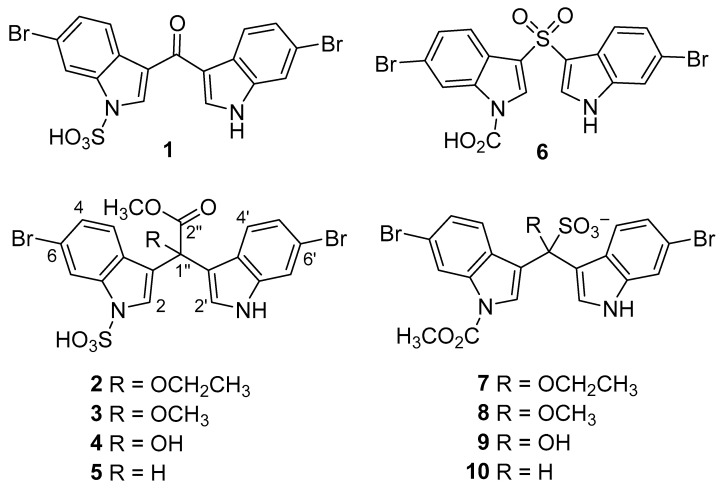
Revised structures for echinosulfone A (**1**) and the echinosulfonic acids A-D (**2**–**5**), alongside their incorrectly reported structures **6**–**10**.

With several online databases housing NP structures and their metadata [4], such as MarinLit [2], COCONUT [5], and the Dictionary of Natural Products [6], cheminformatics techniques have evolved into excellent analytical tools for handling large datasets. Recently, we have demonstrated the analytical power of cheminformatics, specifically utilising machine learning, self-organising maps (SOM), and principal component analyses. Our research has not only explored the structural diversity of NPs (by subclass, terrestrial vs. marine, and producing phyla) [1,3,7], but also examined the bioactivities and the diversity of bioassays used for their biological evaluations [1,3]. Despite advancements in 2D NMR spectroscopy and access to an ever-increasing toolbox of orthogonal structure elucidation and NMR fact-checking techniques [8,9,10], the reporting of incorrect NP structures continues to permeate the literature. Incorrectly assigned molecules can have far-reaching consequences, particularly in multidisciplinary fields where NPs are a central focus such as NP biosynthesis, molecular biology, chemical ecology, synthetic biology, agriculture, and drug discovery and development. To date, we have reported structure revisions for nearly twenty terrestrial and marine NPs, including a series of brominated sponge-derived bis-indole alkaloid sulfamates, echinosulfone A (**1**) and the echinosulfonic acids A−D (**2**–**5**, Figure 1) [11]. 

The co-isolates, echinosulfone A (**6**) and the echinosulfonic acids A–C (**7**–**9**), inappropriately named based on their incorrectly assigned structures, were reported from the Australian sponge *Echinodictyum* sp. [12], while echinosulfonic acid D (**10**), its structure based on the incorrectly assigned co-isolate **8**, was reported as part of a bioassay-guided investigation of the New Caledonian sponge *Psammoclema* sp. [13]. Their structures were subsequently revised to indole sulfamates **1**–**5** based on reinterpretation of their reported NMR and MS experimental data, the total synthesis of echinosulfone A (**1**), and what was clearly more plausible biosynthetic grounds [11]. The revised structures **1**–**5** were supported by the re-isolation of echinosulfone A (**1**) and echinosulfonic acid B (**3**) [14], while a second synthesis of echinosulfone A (**1**) was also published [15]. Recently, the total synthesis of echinosulfonic acid D (**5**) was reported by Abe et al. using a multi-step umpolung method with dimethylbarbituric acid [16].

**Figure 2:**
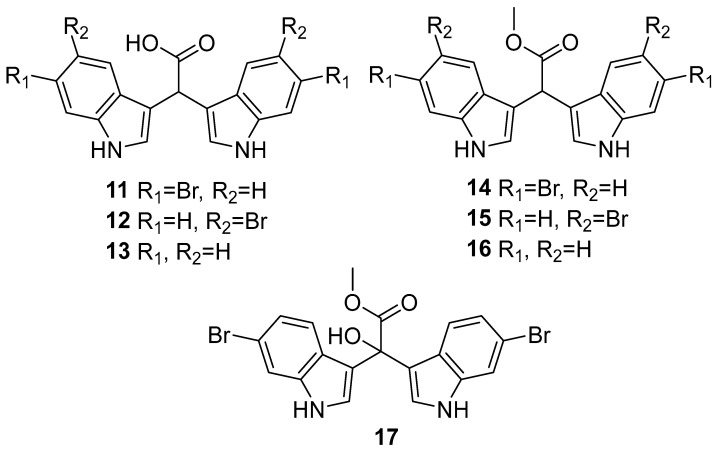
Bis(3′-indolyl) α-methine acetic acids **11**–**13** and acetates **14**–**16**, and 6-dibrominated bis(3′-indolyl) α-hydroxy acetate **17**.

While the structures of echinosulfone A (**1**) and echinosulfonic acids A–D (**2**–**5**) have now been formally revised, synthetic efforts toward these interesting marine bis-indole alkaloid scaffolds present both inspiration and challenges. Such endeavours prove valuable for exploring and expanding NP biological activities. Herein, we describe an acid-catalysed double Friedel–Crafts reaction affording relatively simple access to brominated and non-brominated α-methine bis(3′-indolyl) acetic acids and acetates (**11**–**16**, Figure 2). In addition, utilising the aforementioned bis(3′-indolyl) α-methine acetate scaffolds and exploiting a proposed indole azafulvenium pathway, we report the first synthesis of α-hydroxy bis(3′-indolyl) acetate scaffolds and the full NMR characterisation of 6-brominated α-hydroxy bis(3′-indolyl) alkaloid **17** (Figure 2). Despite the instability of **17** (and the partially purified debromo analogue **18**), this marks the first successful synthetic approach toward the elusive echinosulfonic acid C (**4**) scaffold outside of sponge secondary metabolism.

The synthesis of the bis-indoles presented herein also led to the re-examination of 16 synthetic bis(3′-indolyl) alkaloids (**3o′**, **5a**–**n**, and **5ab′**) [17]. This series of synthetic bis-indoles were assigned as α-hydroxy bis(3′-indolyl) alkaloids. However, discrepancies identified in the reported NMR and MS data for the synthetic α-hydroxy bis-indoles raised concerns regarding the accuracy of their assigned structures and the outcomes claimed for their acid-catalysed Friedel–Crafts synthetic strategy with acylsilane [17]. Comparative analyses of NMR and MS experimental data with the data obtained for the synthetic bis-indoles reported in this study (**11**–**18**) conclusively established the true identities of all 16 synthetic indoles as α-methine bis(3′-indolyl)s, further reinforcing the difficulties associated with the laboratory synthesis of α-hydroxy bis-indoles.

Furthermore, we employed cheminformatics analyses of MIA structural diversity (*n* = 2048) integrated with the findings from our recent meta-analysis of MIA bioactivities [1] to explore the biologically relevant chemical space of the synthetic bis(3′-indolyls) **11**–**17**. Also included in these analyses were synthetic mono- and dibrominated bis(3′-indolyl) methanones **19**–**22** synthesised via our previously reported method [11]. Using a self-organising map (SOM) of MIA structural diversity scaled for biological activity, the synthetic bis-indoles were identified to share chemical space with other known marine bis-indoles reported with antibacterial activities. Their predicted antibacterial activities were subsequently examined against methicillin-susceptible and methicillin-resistant *Staphylococcus aureus* (MSSA and MRSA) and *Pseudomonas aeruginosa*. The effectiveness of cheminformatics-guided approaches in targeting biological evaluations was underscored by the activity of dibrominated bis(3′-indolyl) α-methines **11**, **12**, **14**, and **15** against both MSSA and MRSA. Notably, 6-dibromo bis(3′-indolyl) acetate **14** exhibited the highest potency with MIC_50_ values of 4 µg/mL and 8 µg/mL against MSSA and MRSA, respectively.

## 2. Results

### 2.1. Synthesis of α-Methine Bis(3′-indolyl) Acetic Acids and Methyl Acetates (**11**–**16**)

**Scheme 1:**
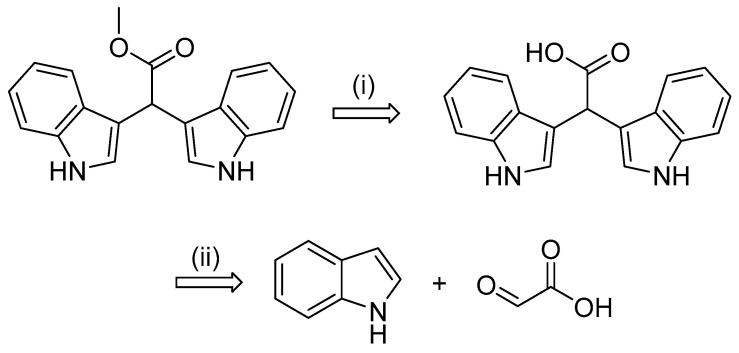
Retrosynthetic analysis of representative α-methine bis(3′-indolyl) methyl acetate scaffold (**16**); (i) esterification of carboxylic acid and (ii) bis-indole formation.

Our ongoing interest in exploring MIA diversity coupled with the recent total synthesis of echinosulfonic acid D (**5**), using a multi-step umpolung method with dimethylbarbituric acid [16], inspired design of a more direct approach toward accessing the structural diversity of desulfonated MNP-inspired α-methine bis(3′-indolyl) acetate scaffolds. A simple disconnection of the α-methine bis(3′-indolyl) methyl acetate (**16**) suggested the adoption of a two-step synthetic approach (Scheme 1).

A modified Lewis-acid-catalysed Friedel–Crafts method, based on that reported by Sathieshkumar et al. for the total synthesis of the tris-indole natural product pseudellone C [18], formed the basis of our double Friedel–Craft bis-indole formation step. However, pyruvic acid was exchanged for glyoxylic acid in our strategy with the aim of accessing the required α-methine bis(3′-indolyl) acetic acid which we reasoned could be esterified to afford the bis-indole methyl acetate.

**Scheme 2:**
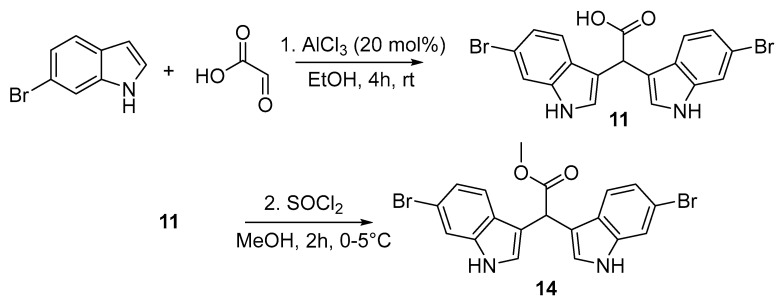
Acid-catalysed double Friedel–Crafts reaction to afford 6-dibromo α-methine bis(3′-indolyl) acetic acid **11** followed by thionyl chloride mediated esterification to 6-dibromo α-methine bis(3′-indolyl) methyl acetate **14**.

Using aluminium chloride (AlCl_3_) as the catalyst (20 mol%), 2 equivalents (eq.) of indole (either 5-bromo, 6-bromo, or non-brominated indole) were reacted with glyoxylic acid (1.2 eq.) in a stirred solution of anhydrous EtOH under an inert atmosphere, and the reaction mixture was monitored by thin-layer chromatography (TLC) for the disappearance of starting material (Scheme 2). After 4 h, the reaction mixture was quenched with cold H_2_O. After careful pH-monitored acid–base workup, the desired α-methine bis(3′-indolyl) acetic acids (**11**–**13**) were obtained in excellent yields (>88%) without the need for further purification. Following this, the α-methine bis(3′-indolyl) acetic acids (**11**–**13**, Figure 2) were esterified by stirring thionyl chloride in anhydrous MeOH at 0−5 °C under inert conditions for 2 h (Scheme 2). The reaction mixture was quenched with cold H_2_O, and after careful extraction with EtOAc and water, the organic phase was concentrated to dryness to afford quantitative yields of halogenated and non-halogenated α-methine bis(3′-indolyl) methyl acetates (**14**–**16**, Figure 2). The synthetic strategy outlined above provides a straightforward approach for synthesising a diverse range of bis-indole acids and esters, requiring only one step for bis-indole acids and two steps for bis-indole methyl esters. Furthermore, the synthetic bis-indoles were obtained in excellent yields without the need for any silica-based purification steps.

### 2.2. Synthesis of α-Hydroxy Bis(3′-indolyl) Methylacetates (**17**−**19**)

**Scheme 3:**
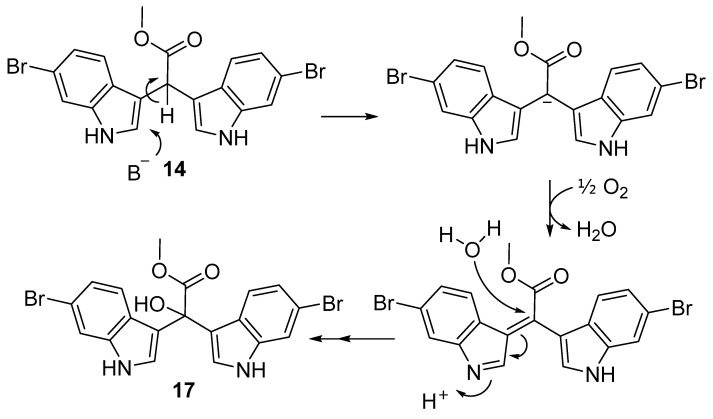
Proposed base-catalysed aerial oxidation bis-indole azafulvenium pathway from α-methine bis-indole scaffold **14** toward α-hydroxy bis-indole **17**.

With a robust approach in place for the synthesis of α-methine bis(3′-indolyl) methyl acetates (**14**–**16**), our focus shifted to the sponge NP α-hydroxy bis(3′-indolyl) acetate scaffolds, the echinosulfonic acids A–C (**2**–**4**). To the best of our knowledge, no syntheses of α-hydroxy bis(3′-indolyl) acetate scaffolds have been reported in the literature. Sala et al. reported the desulfonation products of echinosulfone A (**22**) and echinosulfonic acid B (**3a**, Figure S47) resulting from their chemical investigation of a Western Australian *Crella* sponge species [14]. The desulfonation products were rationalised to form via an acid-catalysed azafulvenium pathway initiated by the loss of sulfonic acid from indole, with the postulated azafulvenium intermediate reported as an intense purple colour. However, we propose that both desulfonated echinosulfone A and echinosulfonic acid B are more likely to have simply hydrolysed, losing sulfuric acid. Regardless, in both cases an azafulvenium intermediate would likely provide stability. With this in mind, we explored the prospect of bis-indole azafulveniums that we could generate from our synthetic α-methine bis(3′-indolyl) methyl acetates **14** and **16** to make α-hydroxy bis(3′-indolyl) methyl acetates. We hypothesised that compounds **11**–**16** could be oxidised under basic conditions by exploiting the acidity of the methine proton H-1″. We had previously noted intense deep purple colours in certain reaction mixtures during the optimisation of the Friedel–Crafts reaction for the synthesis of the bis-indole acids (**11**–**13**) and suspected that this colouration might be attributed to an aerial oxidation process. We reasoned that upon oxidation of the bis-indole scaffold to the bis-indole azafulvenium, we could then introduce the tertiary alcohol group at C-1″ to form the α-hydroxy bis(3′-indolyl) alkaloid scaffold (Scheme 3).

**Scheme 4:**
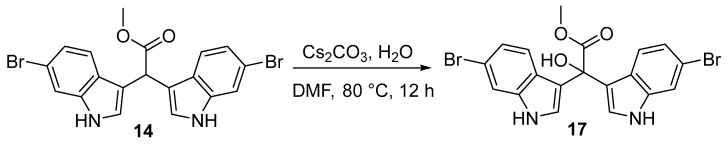
Synthesis of the desulfonated echinosulfonic acid C scaffold 6-dibromo α-hydroxy bis(3′-indolyl) acetate (**17**) from 6-dibromo α-methine bis(3′-indolyl) acetate (**14**) via what is likely a base-catalysed bis-indole azafulvenium intermediate.

To preserve the integrity of bis-indole acetate scaffolds (**14** and **16**), we opted for the mild base cesium carbonate (Cs_2_CO_3_) in lieu of stronger alternatives. Our base-catalysed approach involved heating **14** in DMF to around 80 °C, followed by the addition of 1.5 eq. of Cs_2_CO_3_. The reaction mixture was left to stir for 12 h (Scheme 4). Upon a noticeable colour change from red to deep purple after several hours, the reaction mixture was quenched with H_2_O, cooled to room temperature, and stirred for an additional 15 min. Following careful extraction with EtOAc, the organic phase was adsorbed to C_18_-bonded silica gel and loaded into a refillable guard column. Subsequently, the C_18_-infused reaction mixture underwent purification via preparative reversed-phase high-pressure liquid chromatography (RP HPLC) using a decreasing polarity solvent gradient from H_2_O to CH_3_CN over 60 min, with fractions collected each minute. Fractional ^1^H NMR analysis confirmed that HPLC fractions 27–30 contained pure methyl-2,2-bis(6-bromo-1*H*-indol-3-yl)-2-hydroxyacetate (**17**).

The HSQC and ^13^C NMR spectra for **17** displayed an oxygenated, non-protonated carbon resonance at δ_C_ 73.8 (C-1″), while the ^1^H NMR and HSQC spectra contained a singlet proton resonance at δ_H_ 6.13 not directly attached to carbon, thereby representing the OH proton attached to C-1″. To the best of our knowledge, compound **17** represents the first reported synthesis and full NMR characterisation of a synthetic bis-indolyl-α-hydroxyacetate. The ^1^H and ^13^C NMR data for **17** were also identical to those reported for the non-sulfated 6-bromoindole, α-hydroxy carbon, and methyl ester resonances in echinosulfonic acid C (**4**), thus providing the first direct experimental evidence to corroborate the revised structure of echinosulfonic acid C.

**Scheme 5:**
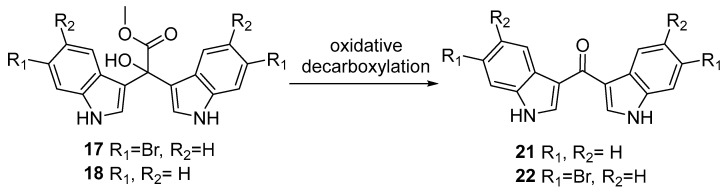
Oxidative decarboxylation of the bis-indolyl-α-hydroxyacetates **17** and **18** to their respective ketone-bridged bis-indole methanones **21** and **22**.

Using our base-catalysed azafulvenium method, we were also able to synthesise the non-brominated α-hydroxy bis-indole **18**; however, once the hydroxyl was installed at C-1″, the compound readily underwent oxidative decarboxylation to the bis-indole ketone **21** during HPLC purification (polar aprotic solvents) and concentration under vacuum (Scheme 5).

The oxidative decarboxylation of C-1″ hydroxy bis(3′-indolyl) methyl acetates **17** and **18** to ketone-bridged bis-indoles **22** and **21** respectively, was observed during HPLC purification (particularly with MeOH as eluent) and concentration of samples in polar aprotic solvents under heat and vacuum. The ^1^H NMR spectra depicting the decarboxylation of non-brominated hydroxyacetate **18** to **21** during HPLC purification are displayed in Figure S30. The instability of the synthetic α-hydroxy bis-indole methyl acetates **17** and **18** provides evidence for the biosynthetic relationship between echinosulfone A (**1**) and the echinosulfonic acids A–D (**2**–**5**). Our findings suggest that **1** is likely the result of oxidative decarboxylation of any of the echinosulfonic acids A–D (**2**–**5**), most likely the hydroxy bis-indole sulfamate **4**. Furthermore, we expect that the ethoxy and methoxy containing echinosulfonic acids A and B (**2** and **3**) are likely to be artifacts generated by solvolysis during extraction and purification with ethanol and methanol, respectively. Precedence for solvolysis was established during the original isolation of echinosulfonic acid A (**2**) extracted in ethanol [12], while echinosulfonic acid B (**3**) reportedly underwent solvolysis with deuterated MeOH [14]. All of these findings suggest that echinosulfonic acid C (**3**) is most likely the authentic sponge-derived NP, with the co-isolates (**1**, **2**, **4**, and **5**) able to be reasonably linked to this scaffold via solvolysis and/or oxidative decarboxylation.

### 2.3. Structure Revision of α-Hydroxy Bis(3′-indolyl) Compounds **3o′**, **5a**–**5n**, and **5ab′**

**Figure 3:**
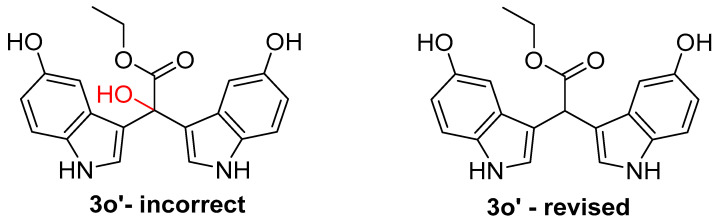
Incorrectly assigned synthetic 1-hydroxy-bis(3′-indolyl) ethylacetate (**3o′-incorrect**) and revised structure of α-methine bis(3′-indolyl) ethylacetate (**3o′-revised**) based on reanalysis of reported NMR and MS experimental data.

While reviewing the literature for synthetic methods reporting other C-1″ hydroxy bis-indoles, we encountered a study reporting the synthesis of 16 α-hydroxy bis(3′indolyl) indoles via an acid-catalysed double Friedel–Crafts strategy [17]. Using 5-hydroxyindole, camphorsulfonic acid, and *tert*-butyldimethylsilane (TBS) glyoxylate in water, the authors proposed a reaction mechanism in which selectivity for α-hydroxy bis-indole formation was attributed to an intermolecular hydrogen bond between C-3 TBS-substituted 5-hydroxyindole and water. After this, a Brook rearrangement occurs to form the 3-TBS 5-hydroxyindole azafulvenium, followed by the loss of TBS and coupling with another 5-hydroxyindole to form α-hydroxy bis(3′indolyl) compounds **3o′**, **5a**–**n,** and **5ab′** (Figure 3 and Figure S45) [17]. Given the challenges installing and maintaining a hydroxyl group at C-1″ encountered during our work, along with the difficulties reported by other research groups [19,20], we carefully examined the published experimental NMR and MS data reported for the 16 synthetic indoles [17].

Upon close inspection of the ^1^H and ^13^C NMR data for **3o′** and the other synthetic analogues, irregularities with its proposed structure were immediately obvious. Most notably, the α-hydroxy carbon C-1″ in **3o′** resonates at *δ*_C_ 40.6, a chemical shift too shielded for an oxygenated quaternary *sp*^3^ carbon. In contrast, C-1″ resonates at *δ*_C_ 73.8 in our synthetic α-hydroxy bis-indole **17**, more than 30 ppm further downfield of that reported for **3o′**. Consistent with the NMR data obtained for **11**–**16** reported herein, the C-1″ carbon resonance for **3o′** was in excellent agreement with the chemical shifts (ranging from *δ*_C_ 39.5 to 40.4) obtained for the α-methine bis(3′-indolyl) acids and methyl esters. In addition, the ^1^H NMR data for **3o′** clearly displayed a singlet proton resonance at *δ*_H_ 5.14 consistent with the α-methine proton at C-1″ in the synthetic bis-indoles (**11**–**16**), all of which resonated between *δ*_H_ 5.30 and 5.57. Moreover, confounding positive-mode HRMS (ESI) spectrometric data reported for the 16 α-hydroxy bis-indoles **3o′**, **5a**–**n**, and **5ab′** were also clearly refuted by the published NMR data. We can only speculate that the [M − (H_2_O) + H]^+^ dehydrated molecules, purportedly obtained from positive-mode HRMS, represent either the azafulvenium bis-indoles we have demonstrated can be generated from α-methine bis-indole acetates, or [M−H]^-^ data acquired in negative-mode ESI HRMS.

Therefore, the structures for α-hydroxy bis(3′indolyl) indoles **3o′**, **5a**–**n**, and **5ab′** should be revised to the α-methine bis(3′indolyl) indole compounds shown in Figure 3 and Figure S45. It is regrettable that neither a ^13^C DEPT nor HSQC NMR experiment was utilised during the characterisation of the synthetic bis-indoles **3o′**, **5a**–**n**, and **5ab′**. Such readily available NMR experiments would have likely revealed the true structural identities of the aforementioned synthetic indoles and avoided inaccuracies associated with the author’s proposed reaction mechanism (Brook rearrangement) and results of their acid-catalysed double Friedel–Crafts reaction with acylsilanes [17].

### 2.4. Cheminformatics-Directed Prediction of Synthetic Bis-Indole Bioactivities

**Figure 4:**
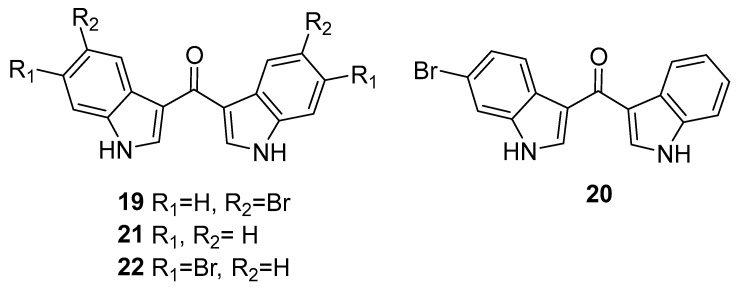
Synthetic bis-indole methanones included in cheminformatics and antibacterial analyses: bis(5-bromo-1*H*-indol-3-yl)methanone (**19**), (6-bromo-1*H*-indol-3-yl)(1*H*-indol-3-yl)methanone (**20**), di(1*H*-indol-3-yl)methanone (**21**), and bis(6-bromo-1*H*-indol-3-yl)methanone (**22**).

The biological activities of the echinosulfonic acid series remain relatively unexplored, with only weak KB cell cytotoxicity reported for the echinosulfonic acids B and D (**3** and **5**) [13], alongside unquantified sponge crude extract anti-parasitic and antibacterial activities [12]. With this in mind, we were interested in exploring the chemical diversity of our synthetic library of bis-indoles compared with that present within MIAs to better direct their biological testing. Based on the results of our recent meta-analysis of MIA chemical diversity and bioactivities [1], we employed a cheminformatics-based approach to guide the biological evaluation of the marine-inspired synthetic bis-indole scaffolds reported herein. Using our existing MIA structure and bioactivity dataset (*n* = 2048 MIAs, with disease and infection targets and the potency of their reported bioactivities according to the formal classifications displayed in Table S1) [1], we integrated our library of synthetic bis-indoles **11**–**17** alongside an additional four ketone-bridged bis-indole methanones synthesised using our previously reported procedure [11]: bis(5-bromo-1*H*-indol-3-yl)methanone (**19**), (6-bromo-1*H*-indol-3-yl)(1*H*-indol-3-yl)methanone (**20**), di(1*H*-indol-3-yl)methanone (**21**), and bis(6-bromo-1*H*-indol-3-yl)methanone (**22**, Figure 4).

**Figure 5:**
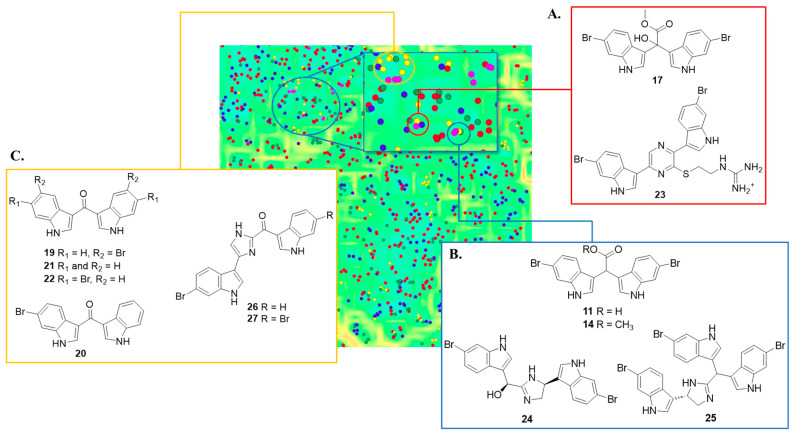
Chemical cluster analysis of MIA dataset (*n* = 2048) integrated with synthetic bis-indoles **11**–**17** and **19**–**22** (pink circles) visualised as a self-organising map (SOM, 50 × 50 neurons) using the Skelspheres 1024-bit chemical fingerprint descriptor. MIAs are coloured according to their reported potency of biological activity (blue = not tested, red = inactive, green = weakly active, and yellow = moderate to potently active). (**A**). Red cluster of synthetic **17** with dragmacidin G (**23**). (**B**). Blue cluster of synthetic 6-dibrominated bis-indoles **11** and **14** with dihydrospongotine C (**24**) and tulongicin (**25**). (**C**). Yellow cluster with bis-indole methanones **19**, **21**, and **22** with bromodeoxytopsentin (**26**) and dibromodexoytopsentin (**27**).

Within the freely available cheminformatics platform Osiris Datawarrior [21], we examined the chemical diversity of the synthetic indoles **11**–**17** and **19**–**22** with that occupied by MIAs (*n* = 2048) visualised in a self-organising map (SOM, 50 × 50 neurons with the SkelSpheres chemical descriptor, Figure S46). SOMs are effective tools for examining chemical diversity and are generated from molecular fragment analyses using neural network algorithms that display structurally similar compounds in clusters of two-dimensional chemical space. The SOM clearly showed that all of the synthetic bis-indoles (**11**–**17** and **19**–**22**) occupied similar areas of chemical space with interesting overlap observed with structurally related bioactive MIAs (Figure 5). The synthetic 6-dibrominated α-hydroxy bis-indole acetate **17** (Figure 5A, red inset) clustered closely with dragmacidin G (**23**), a sponge-derived dibrominated guanidine bis-indole pyrazine reported with potent inhibitory activity against *Staphylococcus aureus* (MIC 0.62 μg/mL) [22] and weak cancer cell line cytotoxicity [23,24]. In addition, the 6-dibromo bis-indole acid **11** and acetate **14** clustered with two bioactive brominated MIAs, the bis-indole dihydrospongotine C (**24**) and the tris-indole tulongicin (**25**, Figure 5B) [25]. Both **24** and **25** were reported with moderate antibacterial activities against *S. aureus* at MIC 1.2 and 3.7 µg/mL, respectively. Moreover, both MIAs were found to be inactive against BSC-1 and HCG-116 cancer cell lines [25].

While the remaining synthetic bis-indoles **12**, **13**, **15**, **16**, and **19**–**22** did not form clusters that directly overlapped with MIAs in our dataset, they did occupy regions of chemical space similar to other MIA bis-indole alkaloids. The bis-indole methanone analogues **19, 21**, and **22** were clustered relatively closely with the bioactive sponge-derived bis-indoles, the topsentins (Figure 5C, yellow inset). While topsentin MIAs have been assigned diverse bioactivities, including cytotoxic, antiviral, and antifungal ones, of most interest was their inhibitory activity against methicillin-resistant *S. aureus* (MRSA) pyruvate kinase (PK) [26]. Both bromodeoxytopsentin (**26**) and dibromodexoytopsentin (**27**) were reported with nanomolar potent and selective inhibition of MRSA PK with IC_50_ values of 60 and 2.1 nM, respectively. Interestingly, structure–activity relationships (SARs) suggest that the halogenated topsentin analogues are favourable for MRSA PK activity [26]. These promising antibacterial results and predicted bioactivity from our cheminformatics analyses therefore prompted our evaluation of synthetic indoles **11**–**17** and **19**–**22** and synthetic echinosulfone A (**1**) [14] against Gram-positive and Gram-negative bacteria.

### 2.5. Antibacterial Testing of Synthetic Bis-Indoles **1**, **11**–**17**, and **19**–**22**

The synthetic bis-indoles **1**, **11**–**17**, and **19**–**22** were evaluated against methicillin-susceptible and methicillin-resistant *S. aureus* (MSSA and MRSA, respectively) and *Pseudomonas aeruginosa*. It was found that four of the bis-indoles were active against MSSA and MRSA, while none displayed activity toward *P. aeruginosa* (Table 1). Most notably, the C-6 dibrominated α-methine bis-indole acetate **14** exhibited the highest potency toward both *S. aureus* strains, with MIC_50_ values of 4 and 8 µg/mL against MSSA and MRSA, respectively. The corresponding C-6 dibrominated α-methine bis-indole acid analogue **11** was also active against both *S. aureus* strains; however, four-fold and two-fold decreases in potency were observed toward MSSA and MRSA compared with the acetate **14**. Additionally, the C-5 dibrominated α-methine bis-indole acid **12** and acetate **15** were also found to be active against MSSA and MRSA, but not at the potencies observed for C-6 dibrominated bis-indole acetate **14**. In contrast, the non-brominated α-methine analogues **13** and **16** and the C-3 ketone-bridged bis-indoles **1**, and **19**–**22** were all found to display no antibacterial activities. There appears to be a clear SAR among the bis-indoles screened with bromination and the presence of either the α-methine acetic acid or methyl acetate, essential for antibacterial activity. This is supported by the complete loss of antibacterial activity when either of the aforementioned structural features is absent. The potency of C-6 dibrominated α-methine bis-indole **14** compared with the acid **11** suggests that the ester is also an important discriminating feature between these two analogues. Consistent with the instability reported above for α-hydroxy bis-indole **17** and **18**, we suggest that **17** likely underwent oxidative decarboxylation to **22** during the course of the antibacterial evaluation.

## 3. Materials and Methods

### 3.1. Chemistry

All anhydrous solvents and reagents used in reactions were purchased from Sigma-Aldrich, except for 5- and 6-bromoindole (Enamine). Further to this, bulk solvents (Merck) were distilled under a normal atmosphere prior to use to ensure non-volatile components were not present—they were not dried. All reactions were carried out under inert N_2_ or argon atmospheres under standard reaction conditions. NMR spectra were recorded at 25 °C on a Bruker^®^ 400 MHz Avance III spectrometer equipped with a 5 mm BBFO probe with Z-gradient and automatic tuning with a SampleCase automatic sample changer, a Bruker Avance III 500 MHz spectrometer (BBFO Smartprobe, 5 mm 31P-109Ag), or a Bruker Avance III HDX 800 MHz with a triple (TCl) resonance 5 mm cryoprobe (all Bruker equipment sourced from Preston, Victoria, Australia). The ^1^H and ^13^C NMR chemical shifts were referenced to the solvent peak for DMSO-*d*_6_ at δ_H_ 2.50 and δ_C_ 39.52. High-resolution negative electrospray ionisation mass measurements were acquired using CH_3_CN as the mobile phase on an Agilent Technologies 6530 Accurate-Mass Q-TOF LC/MS (Mulgrave, Victoria, Australia) with a 1200 Series autosampler and 1290 Infinity HPLC, while low-resolution mass measurements were obtained using a Waters ZQ electrospray ionisation mass spectrometer (Rydalmere, NSW, Australia). A Merck Hitachi L7100 pump equipped with a Merck Hitachi L7455 PDA detector (Bayswater, Victoria, Australia) was used for HPLC purifications. Fractions were collected using a Gilson 215 liquid handler. The solvents used for chromatography were Scharlau HPLC-grade, and H_2_O was Millipore Milli-QPF-filtered (Bayswater, Victoria, Australia). Trifluoroacetic acid (TFA) was spectroscopy-grade from Alfa Aesar, while solvents used for HRESIMS were MS-grade.

### 3.2. Synthetic Procedures

#### 3.2.1. Synthesis of Bis(1H-indol-3-yl)acetic Acids (**11**–**13**)

Based on a procedure described by Sathieshkumar et al. [18], glyoxylic acid monohydrate (1.2 eq.) and AlCl_3_ (20 mol%) were added to a stirred suspension of an appropriate indole (2 eq.) in EtOH under an argon atmosphere and left at room temperature for 3 h. The crude reaction mixture was quenched with cold distilled water (3.0 mL), basified with 1 M NaOH (5.0 mL, pH > 10), and extracted between CHCl_3_ and H_2_O (3 × 30 mL of CHCl_3_). The aqueous phase was collected, acidified with 1 M HCl (5.0 mL, pH < 3), and extracted with EtOAc (3 × 30 mL). The organic phase was dried with Na_2_SO_4_, filtered, and concentrated to dryness without the need for further purification. The α-methine bis-indole carboxylic acids **11**–**13** were afforded in yields greater than 85%.

#### 3.2.2. Synthesis of Bis(1H-indol-3-yl)acetates (**14**–**16**)

Thionyl chloride was added dropwise (1.2 eq.) to the appropriate 2,2-bis(1H-indol-3-yl)acetic acid (**11**–**13**, 1 eq.) under an argon atmosphere; anhydrous MeOH (4–8.0 mL) was then added. The reaction mixture was stirred at 5 °C for 2 h, after which it was quenched with H_2_O (2.0 mL), extracted with EtOAc, dried over Na_2_SO_4_, filtered, and concentrated to dryness without the need for further purification. The α-methine bis-indole acetates **14**–**16** were acquired in yields greater than 90%.

#### 3.2.3. Synthesis of Methyl 2,2-Bis(6-bromo-1H-indol-3-yl)-2-hydroxyacetate (**17** and **18**)

To a stirred suspension of **14** (72 mg, 0.15 mmol) in DMF (2.0 mL) at 80 °C, Cs_2_CO_3_ (145 mg, 0.3 mmol) was added under a normal atmosphere for 12 h. The reaction mixture was quenched with H_2_O (1.0 mL) and left to cool to room temperature. The reaction mixture was extracted in EtOAc, dried over Na_2_SO_4_, and concentrated to dryness. The crude reaction mixture was adsorbed to C_18_-bonded silica gel, loaded into a refillable guard column, and purified by preparative RP HPLC (Kinetex EVO C_18_ 5 μm 100 Å, 21.2 mm × 150 mm) using a solvent gradient from H_2_O to CH_3_CN over 50 min with fractions collected each minute. Compound **17** was afforded in fractions 24 to 27.

#### 3.2.4. Synthesis of Bis(1H-indol-3-yl)methanones (**19**–**22**)

In accordance with our previously reported method [11], 3 M ethyl magnesium bromide (1.2 eq.) was added dropwise under an argon atmosphere at 0 °C to a stirred solution of appropriate indole (1 eq.) in anhydrous diethyl ether (4.0 mL). The reaction mixture was then stirred at room temperature for 2 h, after which it was concentrated to dryness. The resultant indole magnesium salts were resuspended in anhydrous CH_2_Cl_2_ (4.0 mL); then, triphosgene (0.6 eq.) was added under inert conditions, and the reaction mixture was stirred for 24 h at 5 °C. The reaction was quenched with cold distilled H_2_O and carefully partitioned between ethyl acetate and water with the organic phase collected and dried with Na_2_SO_4_. After filtration, the organic phase was concentrated in vacuo. Bis-indoles **19**, **21**, and **22** (^1^H and ^13^C NMR spectra, Figures S43 and S44) were purified by column chromatography on silica gel (3:2 EtOAc/hexanes), while **20** was adsorbed to C_18_-bonded silica gel, loaded into a refillable guard column, and purified by preparative RP HPLC (Kinetex EVO C_18_ 5 μm 100 Å, 21.2 mm × 150 mm) using a solvent gradient from H_2_O (0.1% TFA) to MeOH (0.1% TFA) over 50 min with fractions collected each minute. Compound **20** was afforded in fractions 37 to 39.

2,2-bis(6-bromo-1*H*-indol-3-yl)acetic acid *(***11***):* 350.6 mg (88.7% yield), red amorphous solid; UV(MeOH) λ_max_ (log ε) 287 (5.9), 229 (6.7) nm; IR (neat); *V*_max_ 3410, 1726, 1611, and 1453 cm^−1^; ^1^H NMR (400 MHz, DMSO) δ 11.06 (s, 2H), 7.53 (d, *J* = 1.6 Hz, 2H), 7.43 (d, *J* = 8.5 Hz, 2H), 7.23 (d, *J* = 2.2 Hz, 2H), 7.05 (dd, *J* = 8.5, 2.2 Hz, 2H), 5.30 (s, 1H); ^13^C NMR (101 MHz, DMSO) δ 174.1, 137.3, 125.6, 124.8, 121.4, 120.9, 114.2, 113.9, 113.3, 40.4. HRMS (ESI-QTOF) *m*/*z*: [M−H]^−^ calcd. for C_18_H_11_N_2_O_2_^79^Br^81^Br, (446.9164); found 446.9167. (NMR spectra, Figures S1–S4).

2,2-bis(5-bromo-1*H*-indol-3-yl)acetic acid *(***12***):* 205.7 mg (88.2% yield), red amorphous solid; UV(MeOH) λ_max_ (log ε) 290 (3.1), 228 (3.9) nm; IR (neat); *V*_max_ 3415, 1712, 1611, and 1453 cm^−1^; ^1^H NMR (500 MHz, DMSO) δ 11.16 (s, 2H), 7.68 (d, *J* = 1.8 Hz, 2H), 7.33 (d, *J* = 8.6 Hz, 2H), 7.30 (d, *J* = 2.3 Hz, 2H), 7.17 (dd, *J* = 8.6, 1.8 Hz, 2H), 5.35 (s, 1H); ^13^C NMR (101 MHz, DMSO) δ 174.0, 135.0, 128.2, 125.4, 123.5, 121.4, 113.6, 112.4, 111.1, 40.1; HRMS (ESI-QTOF) *m*/*z*: [M−H]^-^ calcd. for C_18_H_11_N_2_O_2_^79^Br^81^Br, (446.9168); found 446.9167. (NMR spectra, Figures S5–S8).

2,2-di(1*H*-indol-3-yl)acetic acid *(***13***):* 334.1 mg (88.5% yield), red amorphous solid; UV(MeOH) λ_max_ (log ε) 282 (3.0), 222 (3.7) nm; IR (neat); *V*_max_ 3417, 1710, 1614, and 1451 cm^−1^; ^1^H NMR (500 MHz, DMSO) δ 10.91 (s, 2H), 7.54 (d, *J* =7.9 Hz, 2H), 7.35 (d, *J* = 8.0f Hz, 2H), 7.21 (d, *J* = 2.2 Hz, 2H), 7.06 (dd, *J* = 8.0, 7.5 Hz, 2H), 6.94 (dd, *J* = 7.9, 7.5 Hz, 2H), 5.34 (s, 1H); ^13^C NMR (101 MHz, DMSO) δ 174.4, 136.3, 126.5, 123.6, 121.0, 119.0, 118.4, 112.8, 111.5, 40.4. HRMS (ESI-QTOF) *m*/*z*: [M−H]^−^ calcd. for C_18_H_13_N_2_O_2_, (289.0978); found 289.0987. (NMR spectra, Figures S9–S12).

methyl 2,2-bis(6-bromo-1*H*-indol-3-yl)acetate *(***14***):* (300.5 mg, 94.4% yield), dark purple amorphous solid, UV(MeOH) λ_max_ (log ε) 287 (3.2), 228 (4.1) nm; IR (neat); *V*_max_ 3411, 1726, 1612, and 1453 cm^−1^; ^1^H NMR (400 MHz, DMSO) δ 11.12 (br s, 2H), 7.54 (d, *J* = 1.8 Hz, 2H), 7.42 (d, *J* = 8.5 Hz, 2H), 7.25 (d, *J* = 2.2 Hz, d), 7.07 (dd, *J* = 8.5, 1.8 Hz, 2H), 5.47 (s, 1H), 3.64 (s, 3H); ^13^C NMR (101 MHz, DMSO) δ 172.9, 137.2, 125.3, 124.9, 121.5, 120.6, 114.1, 113.9, 112.4, 52.0, 39.5. HRMS (ESI-QTOF) *m/z*: [M−H]^-^ calcd. for C_19_H_13_N_2_O_2_^79^Br^81^Br, (460.9324); found 460.9320. (NMR spectra, Figures S13–S16).

methyl 2,2-bis(5-bromo-1*H*-indol-3-yl)acetate *(***15***):* (176.1 mg, 93.3% yield), red amorphous solid; UV(MeOH) λ_max_ (log ε) 290 (3.4), 222 (4.2) nm; IR (neat); *V*_max_ 3410, 1728, 1610, and 1451 cm^−1^; ^1^H NMR (500 MHz, DMSO) δ 11.2 (s, 2H), 7.66 (d, *J* = 1.8 Hz, 2H), 7.33 (d, *J* = 8.6 Hz, 2H), 7.33 (d, *J* = 2.4 Hz, 2H), 7.17 (dd, *J* = 8.6, 1.9 Hz, 2H), 5.52 (s, 1H), 3.66 (s, 3H); ^13^C NMR (101 MHz, DMSO) δ 173.0 135.0, 128.0, 125.5, 123.6, 121.2, 113.6, 111.8, 111.2, 52.0, 39.6; HRMS (ESI-QTOF) *m/z*: [M−H]^-^ calcd. for C_19_H_13_N_2_O_2_^79^Br^81^Br, (460.9324); found 460.9324. (NMR spectra, Figures S17–S20).

methyl 2,2-di(1*H*-indol-3-yl)acetate *(***16***):* (281.0 mg, 92.5% yield), red amorphous solid; UV(MeOH) λ_max_ (log ε) 336 (2.2), 281 (3.3) 222 (3.9); IR (neat); *V*_max_ 3410, 1728, 1610, and 1451 cm^−1^; ^1^H NMR (500 MHz, DMSO) δ 10.96 (s, 2H), 7.52 (d, *J* = 7.9 Hz, 2H), 7.36 (d, *J* = 8.1 Hz, 2H), 7.06 (ddd, *J* = 8.1, 7.6. 1.0 Hz, 2H), 6.95 (ddd, *J* = 7.9, 7.6, 0.8 Hz, 2H), 5.47 (s, 1H), 3.65 (s, 3H); ^13^C NMR (101 MHz, DMSO) δ 173.1, 136.3, 126.3, 123.7, 121.1, 118.8, 118.5, 112.3, 111.5, 51.8, 40.0. HRMS (ESI-QTOF) *m/z*: [M−H]^−^ calcd. for C_19_H_15_N_2_O_2_, (303.1135); found 303.1132. (NMR spectra, Figures S21–S24).

methyl 2,2-bis(6-bromo-1*H*-indol-3-yl)-2-hydroxyacetate *(***17***):* (7.1 mg, 7.5% yield), orange/brown amorphous solid, UV(MeOH) λ_max_ (log ε) 286 (3.0), 226 (4.1) nm; IR (neat); *V*_max_ 3410, 1726, 1612, and 1453 cm^−1^; ^1^H NMR (800 MHz, DMSO) δ 11.12 (br s, 2H), 7.53 (d, *J* = 1.8 Hz, 2H), 7.29 (d, *J* = 8.5 Hz, 2H), 7.15 (d, *J* = 2.2 Hz, d), 6.99 (dd, *J* = 8.5, 1.8 Hz, 2H), 6.13 (s, 1H), 3.65 (s, 3H); ^13^C NMR (201 MHz, DMSO) δ 174.0, 137.5, 124.9, 124.7, 122.4, 121.4, 117.6, 114.0, 113.8, 73.9, 52.2. HRMS (ESI-QTOF) *m/z*: [M−H]^−^ calcd. for C_19_H_13_N_2_O_3_^79^Br^81^Br, (476.9273); found 476.9268. (NMR spectra, Figures S25–S29).

bis(5-bromo-1H-indol-3-yl)methanone *(***19***):* (178.2 mg, 64.5% yield), brown amorphous solid; UV(MeOH) λ_max_ (log ε) 322 (4.3), 281 (4.6), 253 (4.5), 222 (4.9) nm; IR (neat); *V*_max_ 3419, 2910, 1681, 1597, 1520, and 1034 cm^−1^; ^1^H NMR (400 MHz, DMSO) δ 12.08 (s, 2H), 8.42 (d, *J* =2.0 Hz, 2H), 8.30 (s, 2H), 7.48 (d, *J* = 8.6 Hz, 2H), 7.36 (dd, *J* = 8.6, 2.0 Hz, 2H); ^13^C NMR (101 MHz, DMSO) δ 183.8, 135.2, 133.3, 128.3, 125.2, 123.6, 115.8, 114.0, 113.9. HRMS (ESI-QTOF) *m/z*: [M−H]^−^ calcd. for C_17_H_9_N_2_O^79^Br^81^Br, (416.9062); found 416.9063. (NMR spectra, Figures S31–S34). 

(6-bromo-1*H*-indol-3-yl)(1*H*-indol-3-yl)methanone *(***20***):* (20.1 mg, 24.5% yield), brown amorphous solid; UV(MeOH) λ_max_ (log ε) 324 (4.1), 276 (4.2), 252 (4.1), 219 (4.6) nm; IR (neat); *V*_max_ 3416, 2906, 1678, 1596, 1510, and 1032 cm^−1^; ^1^H NMR (500 MHz, DMSO) δ 11.92 (s, 1H), 11.86 (s, 1H), 8.25 (d, *J* = 8.0 Hz, 1H), 8.20 (s, 1H), 8.19 (d, *J* = 8.5 Hz, 1H), 8.19 (s, 1H), 7.68 (d, *J* = 1.8 Hz, 1H), 7.50 (d, *J* = 8.0 Hz, 1H), 7.32 (dd, *J* = 8.5, 1.8 Hz, 1H), 7.22 (ddd, *J* = 8.0, 7.1, 1.3 Hz, 1H), 7.18 (ddd, *J* = 8.0, 7.1, 1.0 Hz, 1H); ^13^C NMR (101 MHz, DMSO) δ 184.3, 137.4, 136.5, 132.6, 132.3, 126.5, 125.6, 123.9, 123.2, 122.6, 121.5, 121.1, 116.8, 116.6, 115.1, 114.5, 111.9. HRMS (ESI-QTOF) *m/z*: [M−H]^-^ calcd. for C_17_H_10_N_2_O^81^Br, (338.9957); found 338.9963. (NMR spectra, Figures S35–S35).

di(1*H*-indol-3-yl)methanone *(***21***):* (100.3 mg, 70.1% yield), brown amorphous solid; UV(MeOH) λ_max_ (log ε) 321 (4.3), 274 (4.4), 250 (4.3), 217 (4.8); IR (neat); *V*_max_ 3416, 2914, 1676, 1594, 1518, and 1032 cm^−1^; ^1^H NMR (500 MHz, DMSO) δ 11.83 (s, 2H), 8.26 (d, *J* = 7.1 Hz, 2H), 8.16 (s, 2H), 7.50 (d, *J* = 7.8 Hz, 2H), 7.22 (ddd, *J* = 8.3, 7.1, 1.4 Hz, 2H), 7.18 (ddd, *J* = 8.3, 7.8, 1.3 Hz, 2H); ^13^C NMR (125 MHz, DMSO) δ 184.6, 136.5, 132.0, 126.6, 122.6, 121.5, 121.0, 116.8, 111.9. HRMS (ESI-QTOF) *m/z*: [M−H]^−^ calcd. for C_17_H_11_N_2_O, (259.0872); found 259.0875. (Figures S9–S12). (NMR spectra, Figures S39–S40).

### 3.3. Cheminformatics Analyses of Marine and Synthetic Indole Chemical Diversity

The structures for 2048 marine indole alkaloids reported in the MarinLit database (up to the end of 2021) [2] were imported into the freely available cheminformatics software, Osiris DataWarrior [21]. The reported biological activities for the 2048 compounds were scaled to disease and infection targets and potency of activity classifications in Table S1 (as reported in our published meta-analysis of MIAs) [1]. After the integration of the synthetic indoles **11**–**17** and **19**–**22** into the MIA dataset, the chemical diversity of MIAs compared with the synthetic indoles reported herein was visualised using a 50 × 50-neuron self-organising map (SOM) and the SkelSpheres chemical descriptor (1024 bin resolution byte vector encoding for stereochemistry, heteroatoms, and duplicate fragment counts). The SOM output was colour-coded to the potency of the activity of MIAs.

### 3.4. Antibacterial Testing of Synthetic Bis-Indoles **1**, **11**–**17**, and **19**–**22**

Compounds **1** (^1^H and ^13^C NMR spectra, Figures S41 and S42), **11**–**17**, and **19**–**22** were tested for antibacterial activity against two *Staphylococcus aureus* strains (ATCC 25923 and ATCC 43300) and a *Pseudomonas aeruginosa* strain (ATCC 27853). A modified resazurin microtiter plate assay method developed by Sarker et al. was used to determine antibacterial activities [27]. A stock solution of each bis-indole was prepared at 1.5 mM in DMSO, from which a stock plate was prepared using a ten-fold, 1:2 serial dilution. Each assay employed several antibiotic controls which were compared to MIC quality control ranges reported by the Clinical and Laboratory Institute (CLSI) [27]. The positive controls rifampicin and ciprofloxacin were used for MSSA and MRSA strains, while the positive control tobramycin was used for *P. aeruginosa*. For both *S. aureus* stains, 5% DMSO was used as a negative control, while 2.5% DMSO was used for *P. aeruginosa*. Overnight cultures were prepared by aseptically transferring colonies into 10 mL of sterile Luria–Bertani (LB) broth and incubating them for 16–18 h at 37.5 °C. The inoculate was prepared by adjusting the overnight culture to 5 × 10^5^ CFU/mL. The microdilution assay for *S. aureus* was performed in sterile 96-well plates, and to each well, 25 μL of double-strength LB broth, 5 μL of the stock solution, 20 μL of sterile H_2_O, and 50 μL of the inoculate were sequentially added. The volumes for *P. aeruginosa* were double that for *S. aureus*. Plates were incubated for 18 h at 37.5 °C while being shaken at 100 rpm, after which 10 μL of 704 μM resazurin (sodium salt, Sigma Aldrich, Melbourne, Victoria, Australia) was added to all wells and the assay plates were incubated for a further 1 h. Resazurin reduction was recorded on a BMG LABTECH, FLUOstar Omega (Mornington, Victoria, Australia) fluorescent plate reader (lex 544 nm, lem 590 nm). All experiments were run in triplicate over three consecutive days. MIC_50_ values were calculated in GraphPad Prism (version 5) using the log(inhibitor) vs. response (Variable slope) equation. Standard guidelines set by the Clinical and Laboratory Standards Institute were closely followed during each step of the assay [27].

## 4. Conclusions

In conclusion, we report a cheminformatics-guided approach to exploring the bioactivities of MNP-inspired bis-indole alkaloids. Moreover, we also report the first synthesis of the unstable α-hydroxy bis(3′-indolyl) alkaloids (**17**–**19**) with full NMR and MS characterisation of the C-6 dibrominated analogue **17**. The synthesis of desulfato-echinosulfonic acid C (**17**) provides experimental evidence to corroborate the structure revision of echinosulfonic acid C (**4**). In addition, we have demonstrated a robust synthetic strategy toward brominated and non-brominated α-methine bis(3′-indolyl) acids (**11**–**13**) and acetates (**14**–**16**) in one or two steps without the need for further purification. The synthetic results herein provide a simple strategy for accessing diverse bis-indole methanone scaffolds. The success of a cheminformatics-guided exploration of our library of synthetic bis-indoles with 2048 MIAs effectively directed the biological evaluation of **1**, **11**–**17**, and **19**–**22** toward their potential antibacterial activities. The promising MSSA and MRSA activities obtained for the brominated α-methine bis(3′-indolyl) alkaloids, in particular 6-dibrominated bis-indole acetate **14**, validated our cheminformatics predictions based on MIA chemical similarity analyses coupled with reported bioactivity. A clear SAR suggested that both brominated and α-methine bis-indoles were favoured over non-brominated (**13** and **16**) and planar ketone-bridged ones (**1** and **19**–**22**). This work highlights the synergy of NP and synthetic chemistry and the inspiration provided by complex NP scaffolds and the pursuit of their biological potentials. Here we demonstrate the power of cheminformatics-guided approaches for directing the biological evaluation of NP and synthetic scaffolds. It is hoped that more thoughtful strategies aimed at leveraging the plethora of information residing in large NP databases will be used for examining the bioactivities of NPs and NP-inspired compounds. These approaches aim to expand our understanding of NP bioactive chemical space, ultimately maximising future NP drug discovery efforts.

## Figures and Tables

**Table 1:** Antibacterial activities for synthetic indoles **1**, **11**–**17**, and **19**–**22**.

Compound	MIC_50_ (µg/mL (µM))		
	MSSA, ATCC 25923	MRSA, ATCC 43300	Pa, ATCC 27853
**1**	>64 (>128.5)	>64 (>128.5)	>32 (>64.2)
**11**	16 (35.7)	16 (35.7)	>32 (>71.4)
**12**	16 (35.7)	16 (35.7)	>32 (>71.4)
**13**	64 (220.4)	>64 (>220.4)	>32 (>110.2)
**14**	4 (8.7)	8 (17.3)	>32 (>69.2)
**15**	16 (34.6)	16 (34.6)	>32 (>69.2)
**16**	>64 (>210.3)	>64 (>210.3)	>32 (>105.1)
**17**	>64 (>133.9)	>64 (>133.9)	>32 (>66.9)
**19**	>64 (>153.1)	>64 (>153.1)	>32 (>76.5)
**20**	64 (188.7)	64 (188.7)	>32 (>94.3)
**21**	>64 (>245.9)	>64 (>245.9)	>32(>122.9)
**22**	>64 (>153.1)	>64 (>153.1)	>32 (>75.5)
rifampicin	0.25 (0.3)	<0.13 (<0.16)	>32 (>38.9)
ciprofloxacin	0.5 (1.5)	0.5 (1.5)	0.5 (1.5)
tobramycin	-	-	1 (2.1)

## Data Availability

Supporting data are available in the Electronic Supporting Information (ESI) provided.

## Supplementary Materials

The following supporting information can be downloaded at https://www.mdpi.com/article/10.3390/molecules29122806/s1: Figures S1–S44. Experimental NMR spectra for **1** and **11**–**22**; Figure S45. Incorrectly assigned synthetic α-hydroxy bis-indoles (red) and revised α-methine bis-indoles structures (black) for **3o′, 5ab′,** and **5a**–**n**; Figure S46. Chemical diversity of marine indole alkaloids (*n* = 2048) integrated with synthetic bis-indoles **11**–**17** and **19**–**22** visualised as 50 × 50 self-organising map (SOM) using the Skelspheres 1024-bit chemical fingerprint descriptor; Figure S47. Desulfonated echinosulfone A (**22**) and echinosulfonic acid B (**3a**); Table S1. Bioactivity classifications used for cheminformatic analysis of marine indole alkaloids. References [1,3,14,17] are cited in the Supplementary Materials.
