# Supplementary material for: Synthesis and Cheminformatics-Directed Antibacterial Evaluation of Echinosulfonic Acid-Inspired Bis-Indole Alkaloids
Source: Molecules. 2024 Jun 12;29(12):2806. doi: 10.3390/molecules29122806 (PMC11206493; doi:10.3390/molecules29122806)
Supplement: Supplementary file 1 [file molecules-29-02806-s001.zip › molecules-2983455-supplementary.pdf]

Supporting Information

Synthesis and Cheminformatics-Directed Antibacterial Evaluation of Echinosulfonic Acid-Inspired Bis-Indole Alkaloids

Darren C. Holland,[1,2*] Joshua B. Hayton,[1,2] Milton J. Kiefel,[1,3] and Anthony R. Carroll [1,2*]

[1]School of Environment and Science, Griffith University, Southport, QLD, 4222, Australia;

j.hayton@griffith.edu.au (J.B.H); m.kiefel@griffith.edu.au (M.J.K)

[2]Griffith Institute for Drug Discovery, Griffith University, Nathan, Queensland, QLD 4111, Australia

[3]Institute for Glycomics, Griffith University, Southport, Queensland, QLD 4221, Australia

*Correspondence: darren.holland@griffith.edu.au (D.C.H); a.carroll@griffith.edu.au (A.R.C)

1. Table of Contents

**Figure S1**	^{1}H NMR spectrum (500 MHz) of 2,2-bis(6-bromo-1*H*-indol-3-yl)acetic acid (**11**) in DMSO-d_6
**Figure S2**	COSY spectrum (500 MHz) of 2,2-bis(6-bromo-1*H*-indol-3-yl)acetic acid (**11**) in DMSO-d_6
**Figure S3**	HSQC spectrum (500 MHz) of 2,2-bis(6-bromo-1*H*-indol-3-yl)acetic acid (**11**) in DMSO-d_6
**Figure S4**	^{13}C NMR spectrum (125 MHz) of 2,2-bis(6-bromo-1*H*-indol-3-yl)acetic acid (**11**) in DMSO-d_6
**Figure S5**	^{1}H NMR spectrum (400 MHz) of 2,2-bis(5-bromo-1*H*-indol-3-yl)acetic acid (**12**) in DMSO-d_6
**Figure S6**	COSY spectrum (400 MHz) of 2,2-bis(5-bromo-1*H*-indol-3-yl)acetic acid (**12**) in DMSO-d_6
**Figure S7**	HSQC spectrum (400 MHz) of 2,2-bis(5-bromo-1*H* -indol-3-yl)acetic acid (**12**) in DMSO-d_6
**Figure S8**	^{13}C DEPT NMR spectrum (101 MHz) of 2,2-bis(5-bromo-1*H* -indol-3-yl)acetic acid (**12**) in DMSO-d_6
**Figure S9**	^{1}H NMR spectrum (500 MHz) of 2,2-di(1*H*-indol-3-yl)acetic acid (**13**) in DMSO-d_6
**Figure S10**	COSY spectrum (500 MHz) of 2,2-di(1*H*-indol-3-yl)acetic acid (**13**) in DMSO-d_6
**Figure S11**	HSQC spectrum (500 MHz) of 2,2-di(1*H*-indol-3-yl)acetic acid (**13**) in DMSO-d_6
**Figure S12**	^{13}C NMR spectrum (125 MHz) of 2,2-di(1*H*-indol-3-yl)acetic acid (**13**) in DMSO-d_6
**Figure S13**	^{1}H NMR spectrum (500 MHz) of methyl 2,2-bis(6-bromo-1*H* -indol-3-yl)acetate (**14**) in DMSO-d_6
**Figure S14**	COSY NMR spectrum (500 MHz) of methyl 2,2-bis(6-bromo-1*H* -indol-3-yl)acetate (**14**) in DMSO-d_6
**Figure S15**	HSQC NMR spectrum (500 MHz) of methyl 2,2-bis(6-bromo-1*H* -indol-3-yl)acetate (**14**) in DMSO-d_6
**Figure S16**	^{13}C NMR DEPT spectrum (125 MHz) of methyl 2,2-bis(6-bromo-1*H* -indol-3-yl)acetate (**14**) in DMSO-d_6
**Figure S17**	^{1}H NMR spectrum (500 MHz) of methyl 2,2-bis(5-bromo-1*H* -indol-3-yl)acetate (**15**) in DMSO-d_6
**Figure S18**	COSY NMR spectrum (500 MHz) of methyl 2,2-bis(5-bromo-1*H* -indol-3-yl)acetate (**15**) in DMSO-d_6
**Figure S19**	HSQC NMR spectrum (500 MHz) of methyl 2,2-bis(5-bromo-1*H* -indol-3-yl)acetate (**15**) in DMSO-d_6
**Figure S20**	^{13}C NMR spectrum (125 MHz) of methyl 2,2-bis(5-bromo-1*H* -indol-3-yl)acetate (**15**) in DMSO-d_6
**Figure S21**	^{1}H NMR spectrum (500 MHz) of methyl 2,2-bis(1*H* -indol-3-yl)acetate (**16**) in DMSO-d_6
**Figure S22**	COSY NMR spectrum (500 MHz) of methyl 2,2-bis(5-bromo-1*H* -indol-3-yl)acetate (**16**) in DMSO-d_6
**Figure S23**	HSQC spectrum (500 MHz) of methyl 2,2-bis(1*H* -indol-3-yl)acetate (**16**) in DMSO-d_6
**Figure S24**	^{13}C NMR spectrum (125 MHz) of methyl 2,2-bis(1*H* -indol-3-yl)acetate (**16**) in DMSO-d_6
**Figure S25**	^{1}H NMR spectrum (800 MHz) of methyl 2,2-bis(6-bromo-1*H* -indol-3-yl)-2-hydroxyacetate (**17**) in DMSO-d_6
**Figure S26**	COSY NMR spectrum (800 MHz) of methyl 2,2-bis(6-bromo-1*H* -indol-3-yl)-2-hydroxyacetate (**17**) in DMSO-d_6
**Figure S27**	HSQC NMR spectrum (800 MHz) of methyl 2,2-bis(6-bromo-1*H* -indol-3-yl)-2-hydroxyacetate (**17**) in DMSO-d_6
**Figure S28**	HMBC NMR spectrum (800 MHz) of methyl 2,2-bis(6-bromo-1*H* -indol-3-yl)-2-hydroxyacetate (**17**) in DMSO-d_6
**Figure S29**	^{13}C NMR spectrum (201 MHz) of methyl 2,2-bis(6-bromo-1*H* -indol-3-yl)-2-hydroxyacetate (**17**) in DMSO-d_6
**Figure S30**	^{1}H NMR spectrum (500 MHz) of methyl 2-hydroxy-2,2-di(1*H* -indol-3-yl)acetate (**18**, in mixture with **21**) and di(1*H* -indol-3-yl)methanone (**21**) in DMSO-d_6
**Figure S31**	^{1}H NMR spectrum (500 MHz) of bis(5-bromo-1*H* -indol-3-yl)methanone (**19**) in DMSO-d_6
**Figure S32**	COSY spectrum (500 MHz) of bis(5-bromo-1*H* -indol-3-yl)methanone (**19**) in DMSO-d_6

**Figure S33**	HSQC spectrum (500 MHz) of bis(5-bromo-1*H* -indol-3-yl)methanone (**19**) in DMSO-d_6
**Figure S34**	^{13}C DEPT NMR spectrum (101 MHz) of bis(5-bromo-1*H* -indol-3-yl)methanone (**19**) in DMSO-d_6
**Figure S35**	^{1}H NMR spectrum (500 MHz) of (6-bromo-1*H* -indol-3-yl)(1*H* -indol-3-yl)methanone (**20**) in DMSO-*d*
**Figure S36**	COSY spectrum (500 MHz) of (6-bromo-1*H* -indol-3-yl)(1*H* -indol-3-yl)methanone (**20**) in DMSO-d_6
**Figure S37**	HSQC spectrum (500 MHz) of (6-bromo-1*H* -indol-3-yl)(1*H* -indol-3-yl)methanone (**20**) in DMSO-d_6
**Figure S38**	^{13}C DEPT NMR spectrum (125 MHz) of (6-bromo-1*H* -indol-3-yl)(1*H* -indol-3-yl)methanone (**20**) in DMSO-d_6
**Figure S39**	^{1}H NMR spectrum (500 MHz) of di(1*H* -indol-3-yl)methanone (**21**) in DMSO-d_6
**Figure S40**	^{13}C NMR spectrum (125 MHz) of di(1*H* -indol-3-yl)methanone (**21**) in DMSO-d_6
**Figure S41**	^{1}H NMR spectrum (800 MHz) of synthetic echinosulfone A (**1**) in DMSO-d_6
**Figure S42**	^{13}C NMR spectrum (200 MHz) of echinosulfone A (**1**) in DMSO-d_6
**Figure S43**	^{1}H NMR spectrum (500 MHz) of bis(6-bromo-1*H* -indol-3-yl)methanone (**22**) in DMSO-d_6
**Figure S44**	^{13}C NMR spectrum (125 MHz) of bis(6-bromo-1*H* -indol-3-yl)methanone (**22**) in DMSO-d_6
**Figure S45**	Incorrectly assigned synthetic α-hydroxy bis-indoles (red) and revised α-methine bis-indoles (black) for **3o′, 5ab′,** and **5a-n**.
**Figure S46**	Chemical diversity of marine indole alkaloid (*n* = 2048) integrated with synthetic bis-indoles **11-17** and **19-22** visualized as 50 x 50 self-organizing map (SOM) using the Skelspheres 1024-bit chemical fingerprint descriptor.
**Figure S47**	Desulfonated echinosulfone A (**1a**) and echinosulfonic acid B (**3a**).
**Table S1**	Bioactivity classifications used for cheminformatic analysis of marine indole alkaloids and **1, 11-17,** and **19-22**.

2. References

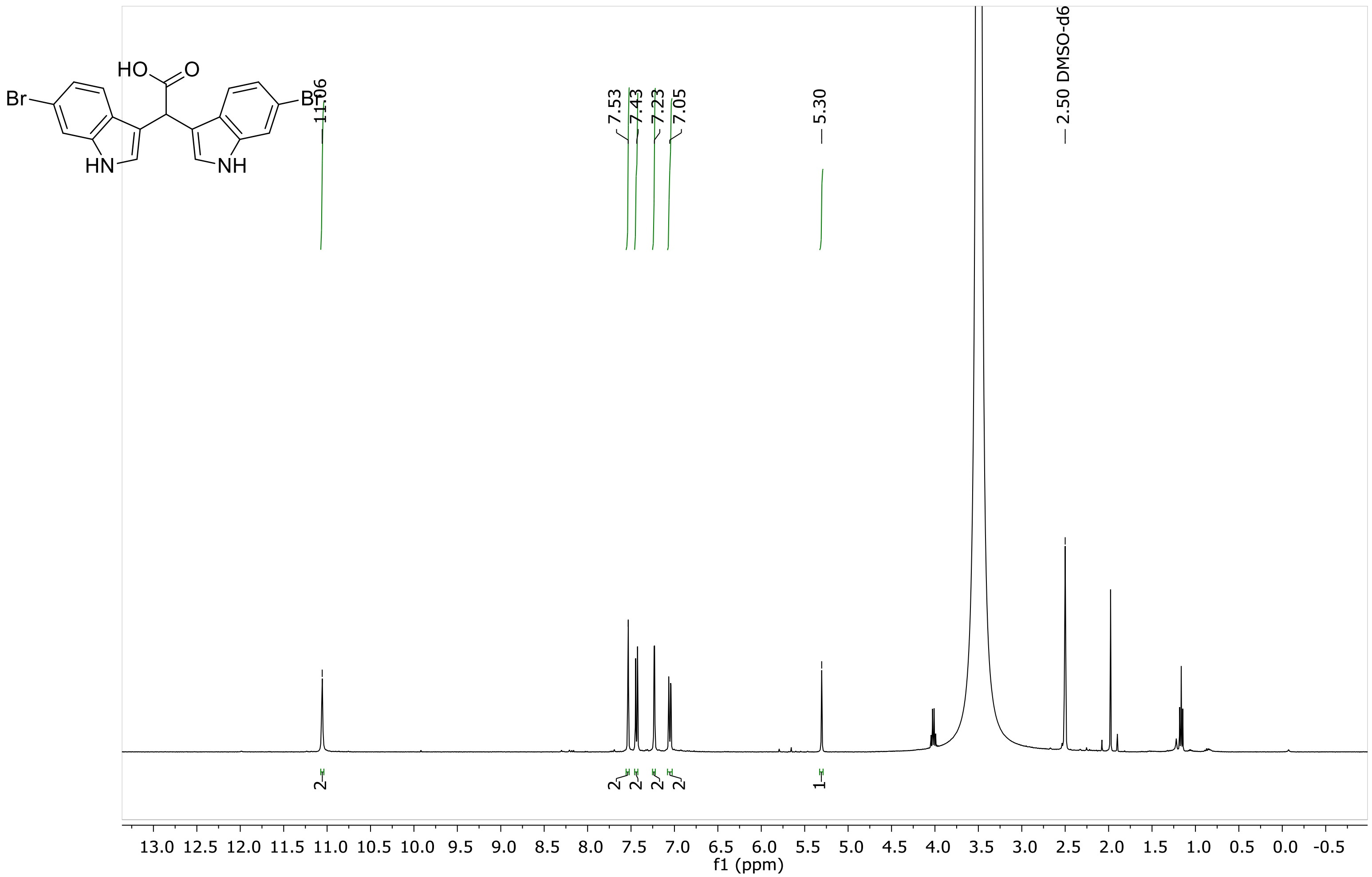

Figure S1 ^{1}H NMR spectrum (500 MHz) of 2,2-bis(6-bromo-1*H*-indol-3-yl)acetic acid (**11**) in DMSO-d_6

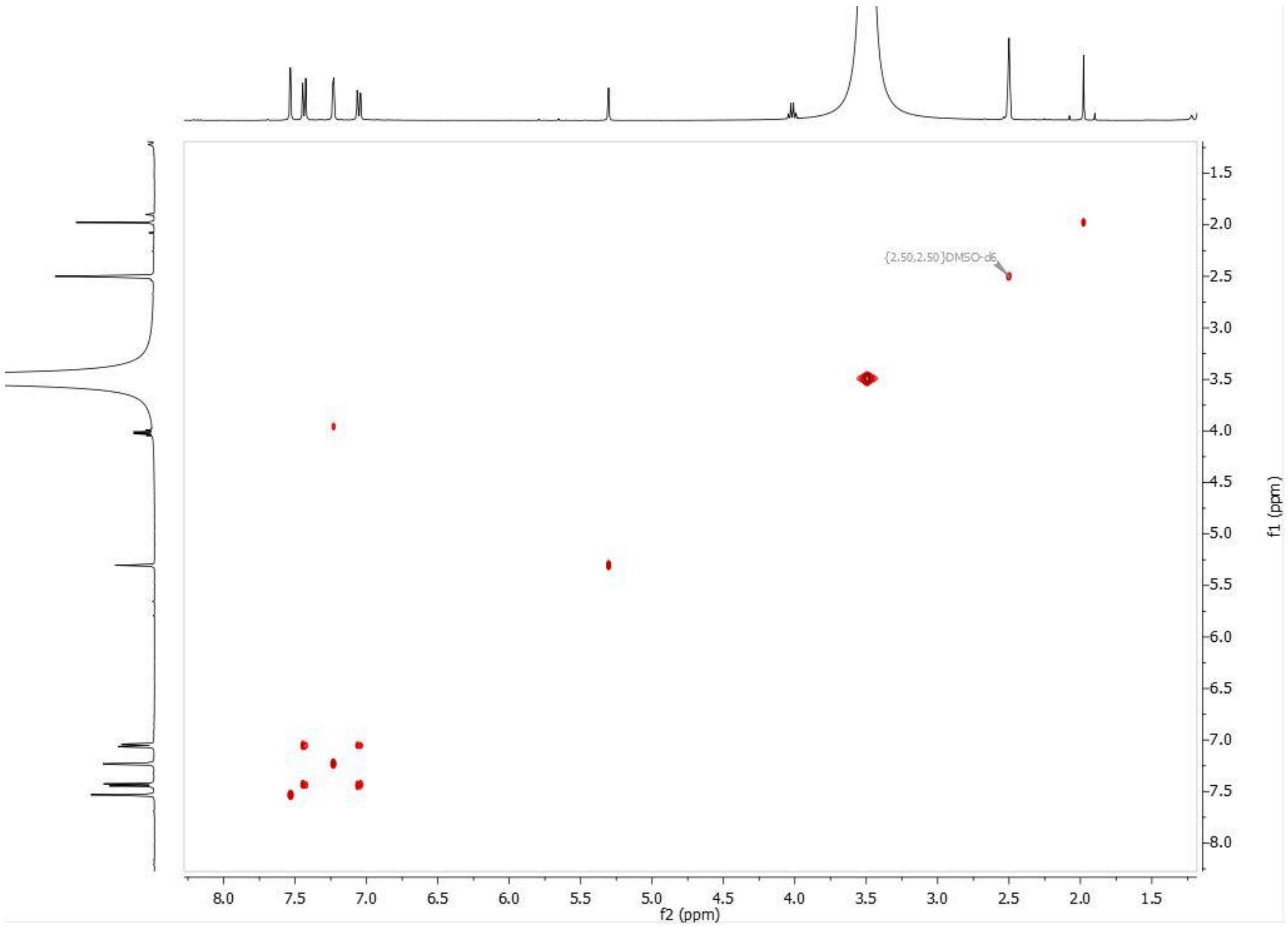

Figure S2. COSY NMR spectrum (500 MHz) of 2,2-bis(6-bromo-1*H* -indol-3-yl)acetic acid (**11**) in DMSO-d_6

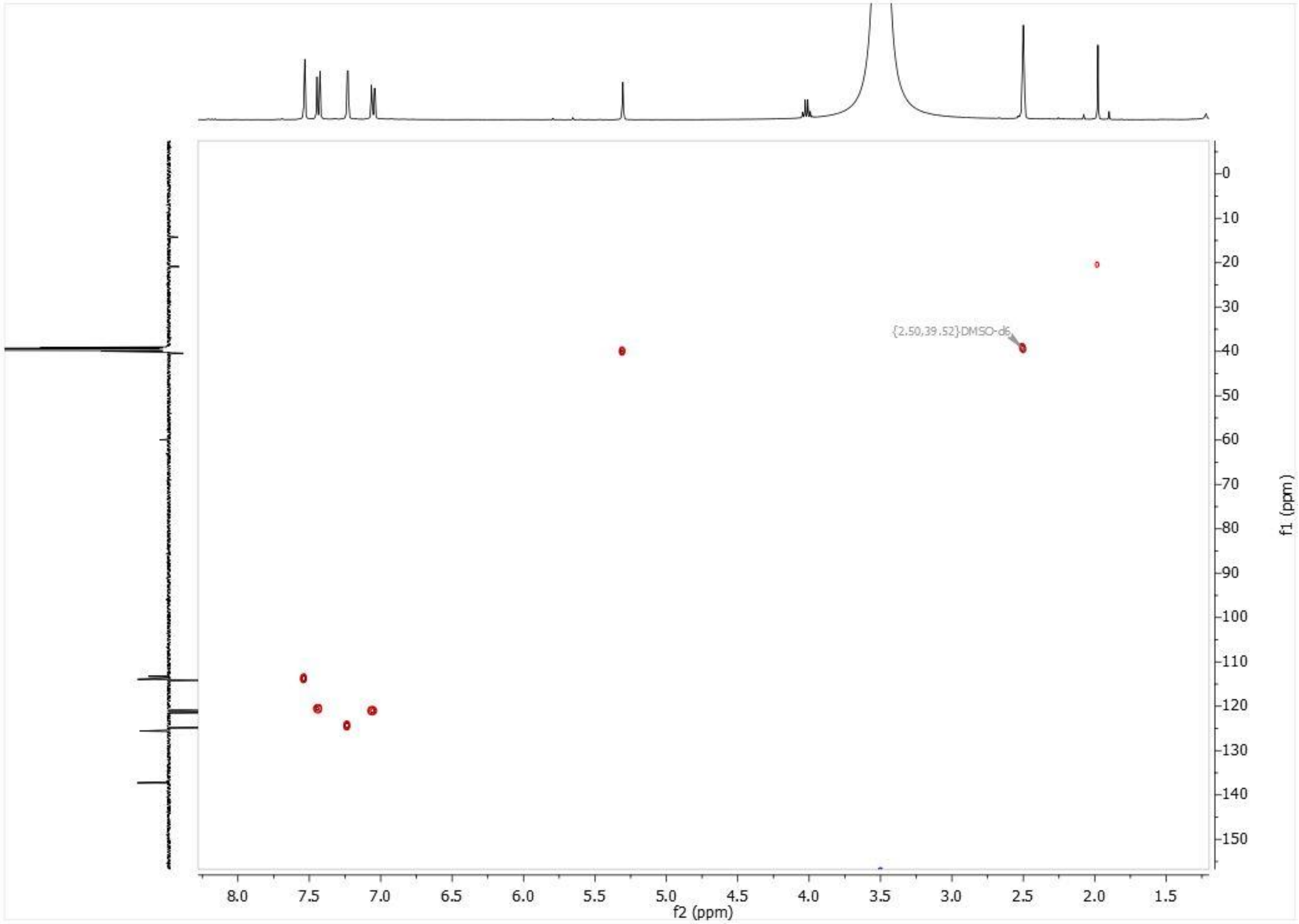

Figure S3. HSQC NMR spectrum (500 MHz) of 2,2-bis(6-bromo-1*H* -indol-3-yl)acetic acid (**11**) in DMSO-d_6

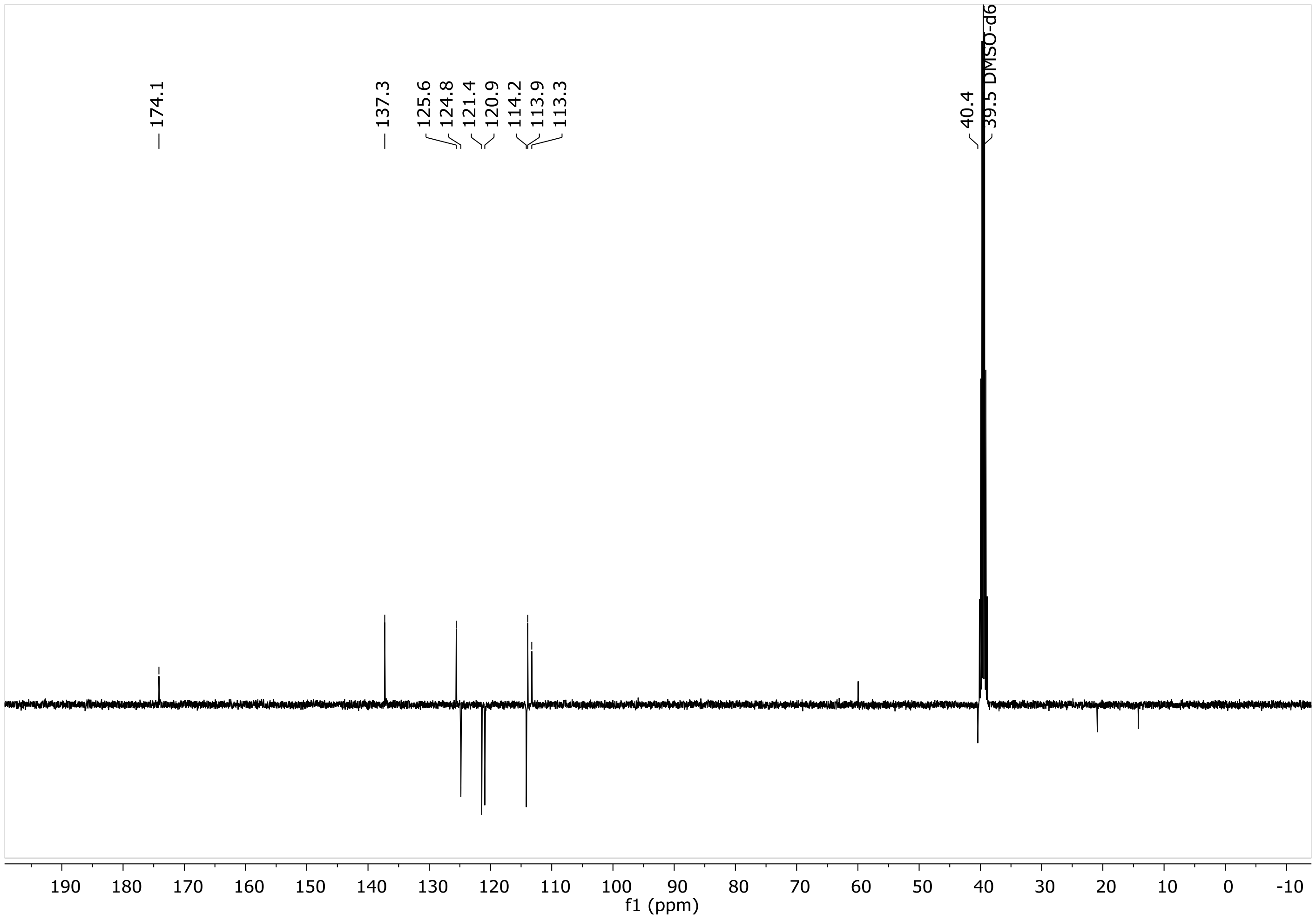

Figure S4. ^{13}C NMR spectrum (125 MHz) of 2,2-bis(6-bromo-1*H* -indol-3-yl)acetic acid (**11**) in DMSO-d_6

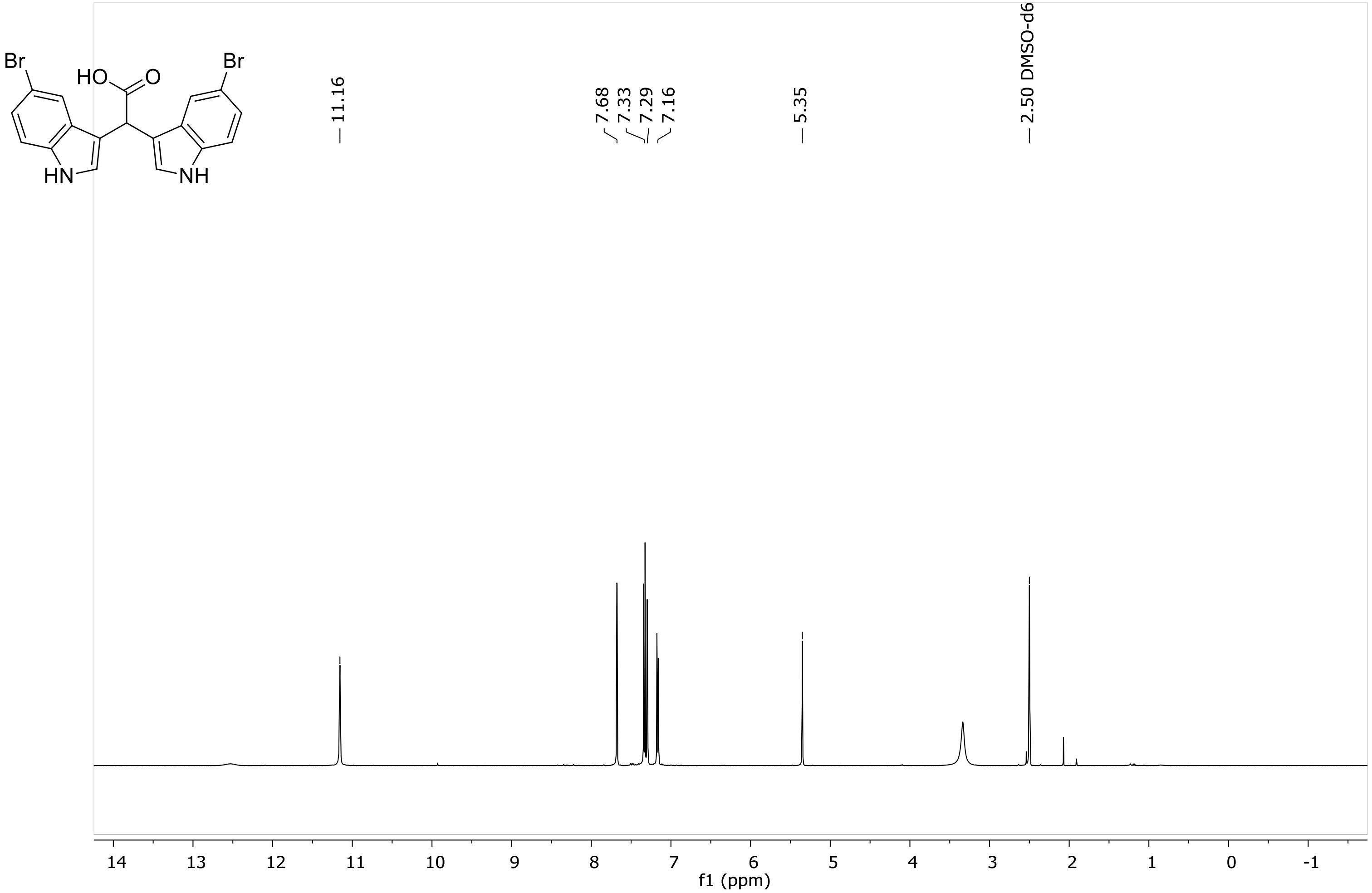

Figure S5. ^{1}H NMR spectrum (500 MHz) of 2,2-bis(5-bromo-1*H*-indol-3-yl)acetic acid (**12**) in DMSO-d_6

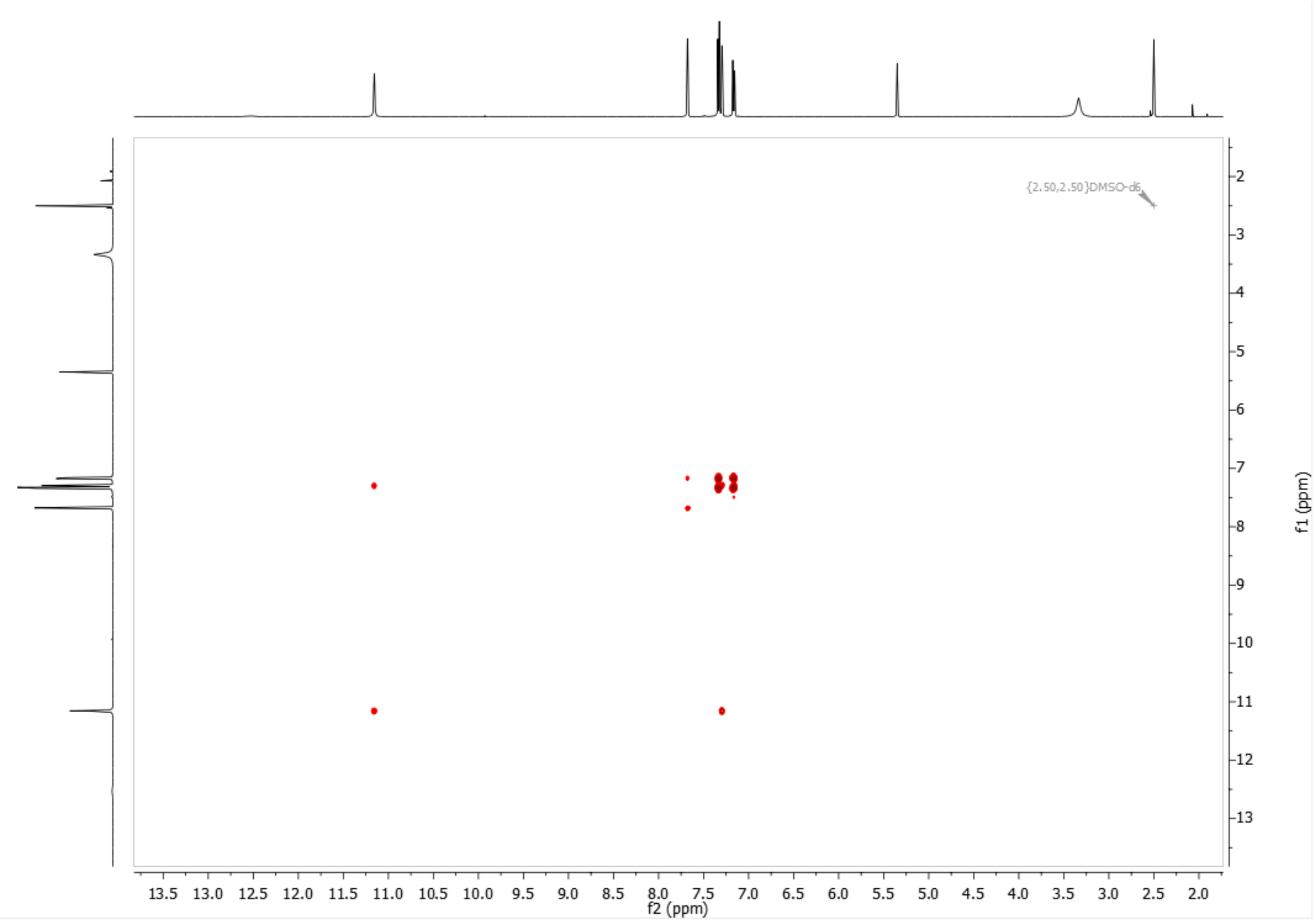

Figure S6. COSY NMR spectrum (500 MHz) of 2,2-bis(5-bromo-1*H* -indol-3-yl)acetic acid (**12**) in DMSO-d_6

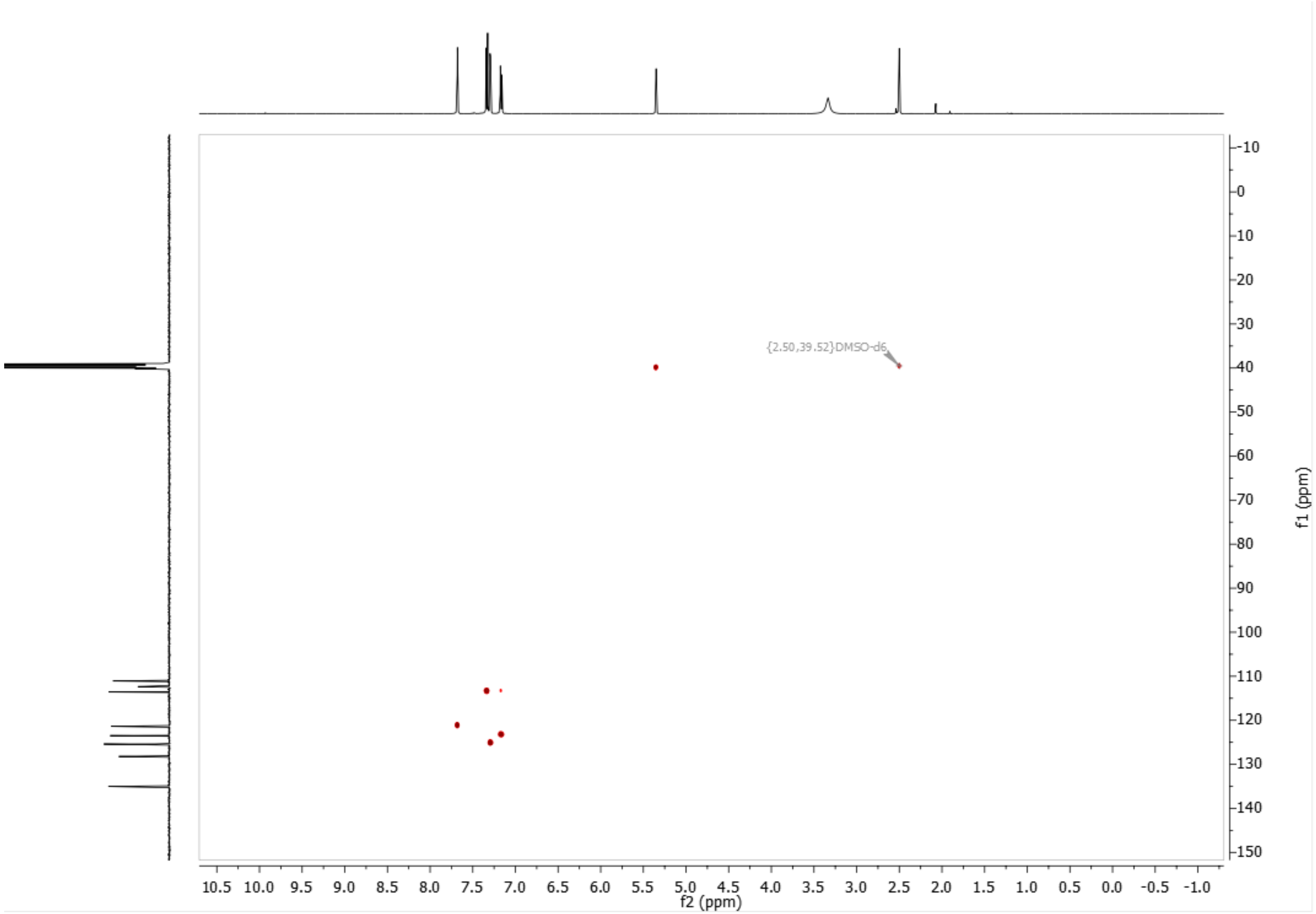

Figure S7. HSQC NMR spectrum (500 MHz) of 2,2-bis(5-bromo-1*H* -indol-3-yl)acetic acid (**12**) in DMSO-d_6

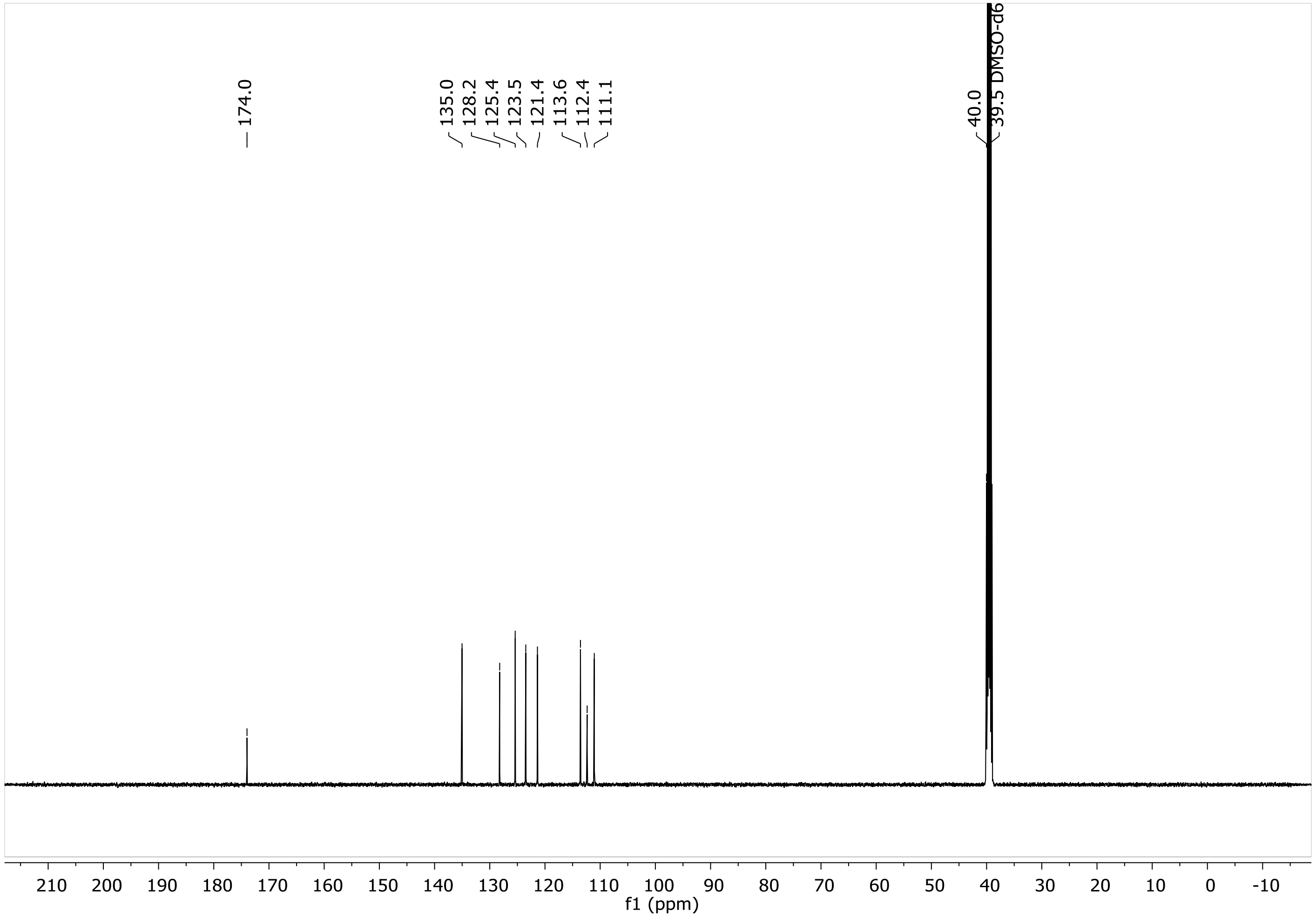

Figure S8. ^{13}C NMR spectrum (125 MHz) of 2,2-bis(5-bromo-1*H* -indol-3-yl)acetic acid (**12**) in DMSO-d_6

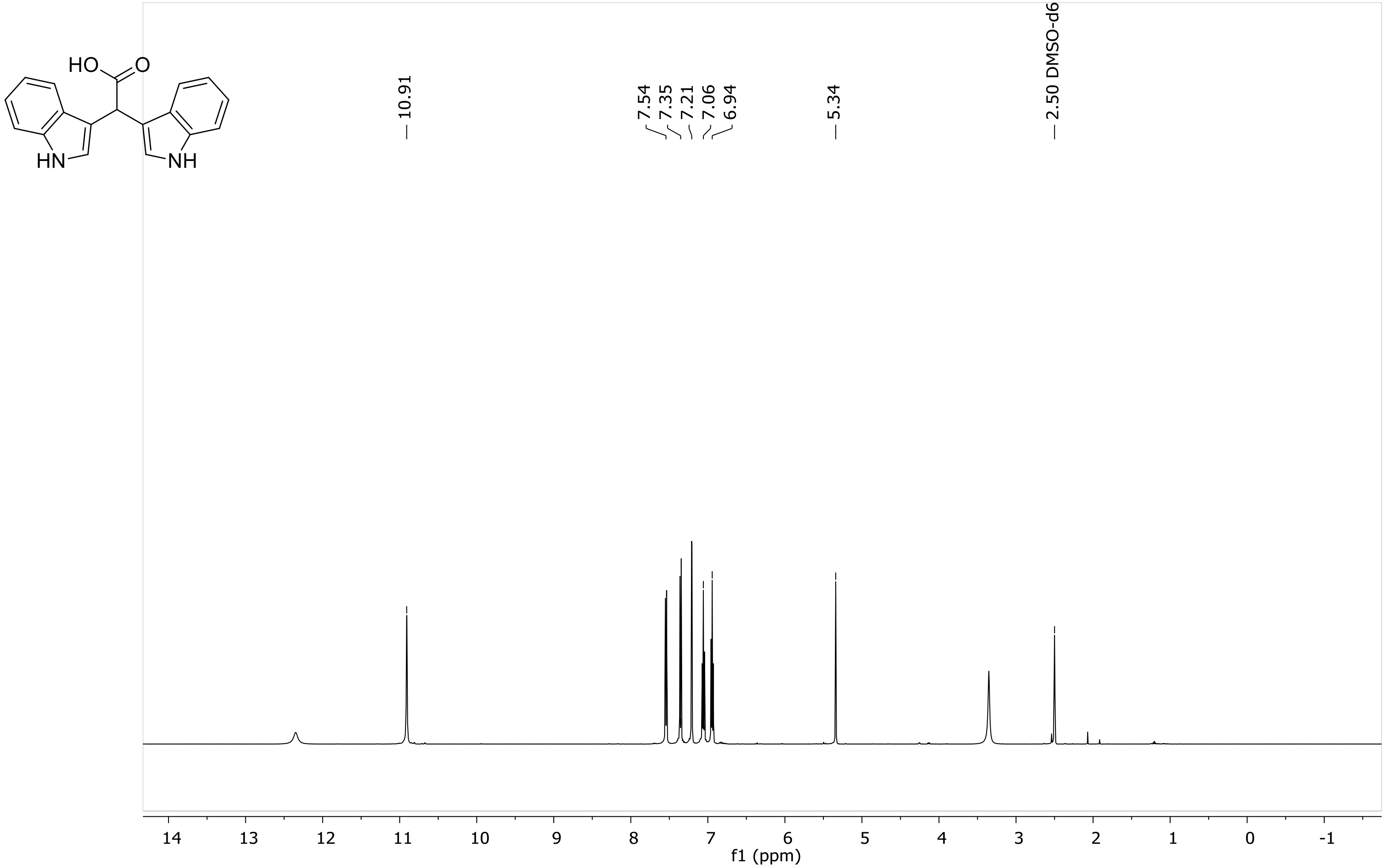

Figure S9. ^{1}H NMR spectrum (500 MHz) of 2,2-di(1*H*-indol-3-yl)acetic acid (**13**) in DMSO-d_6

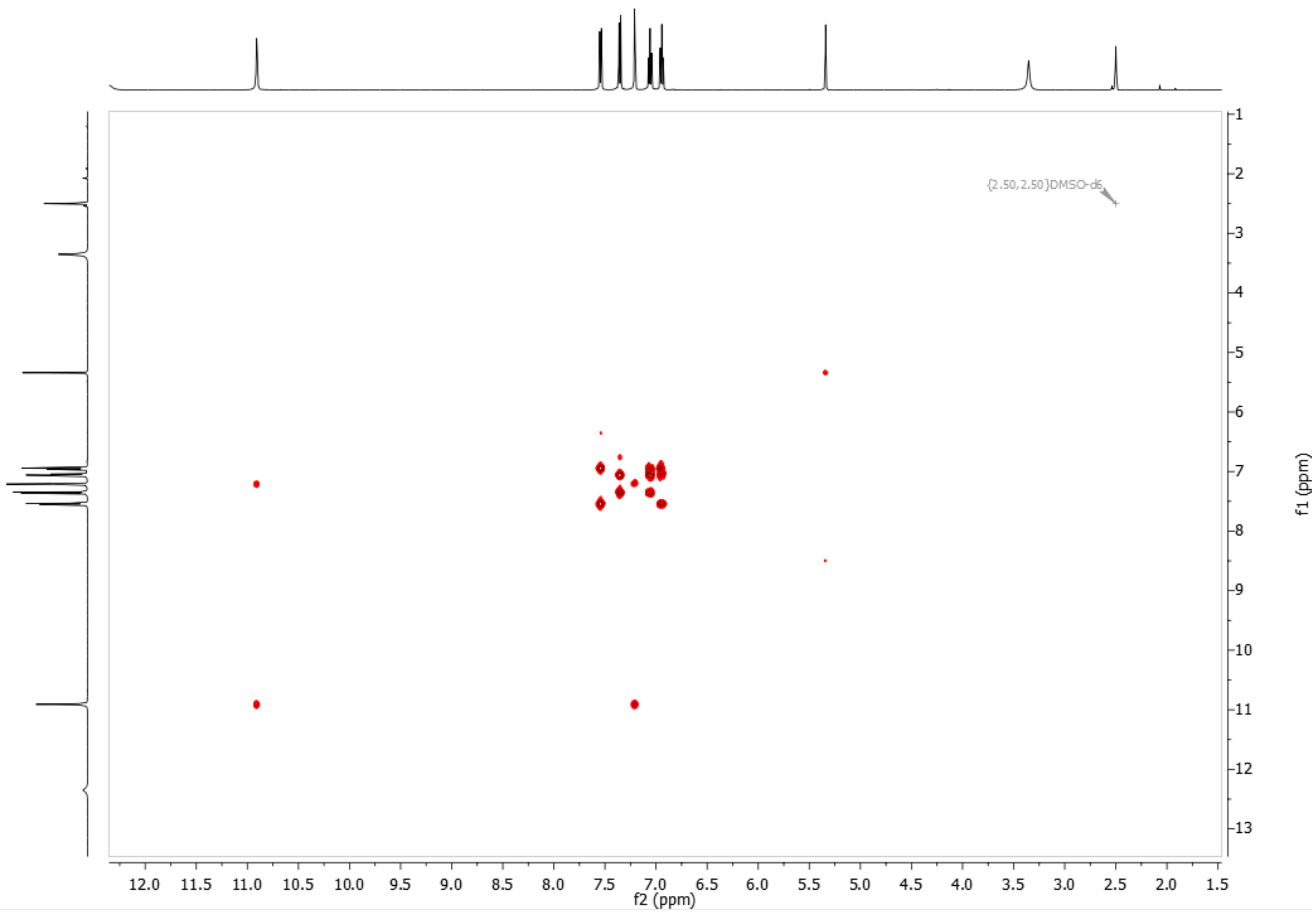

Figure S10. COSY NMR spectrum (500 MHz) of 2,2-di(1*H*-indol-3-yl)acetic acid (**13**) in DMSO-d_6

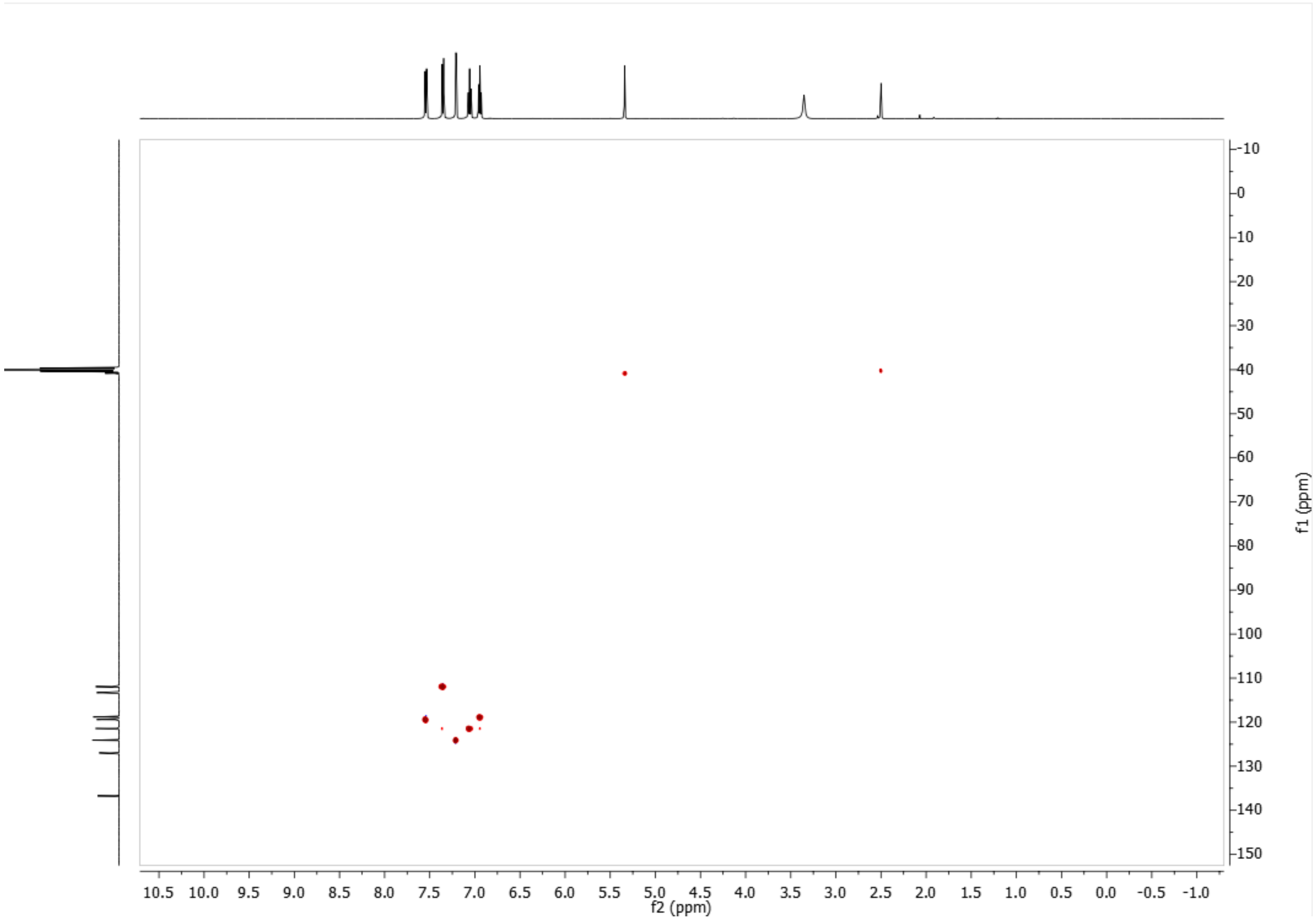

Figure S11. HSQC NMR spectrum (500 MHz) of 2,2-di(1*H*-indol-3-yl)acetic acid (**13**) in DMSO-d_6

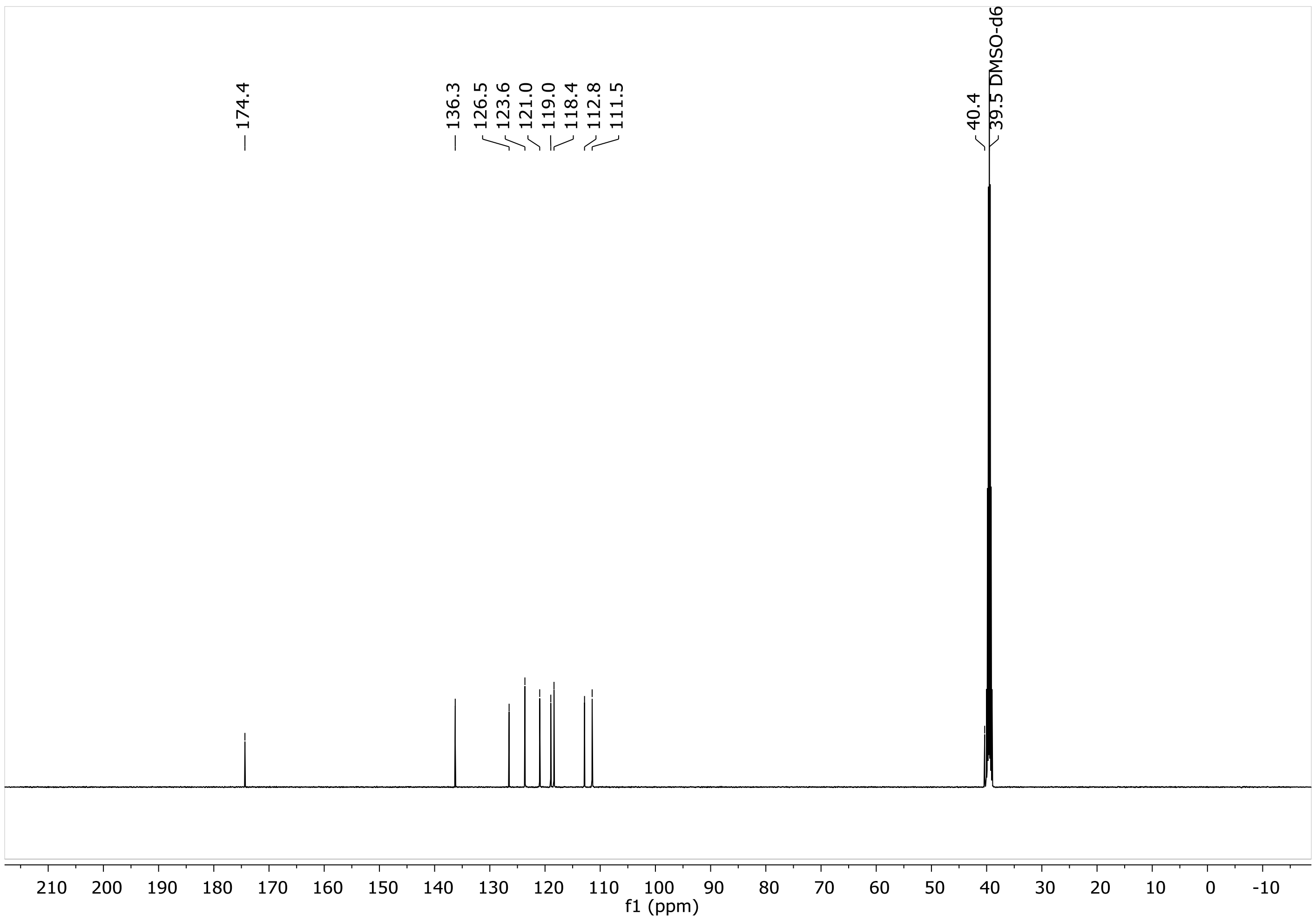

Figure S12. ^{13}C NMR spectrum (125 MHz) of 2,2-di(1*H*-indol-3-yl)acetic acid (**13**) in DMSO-d_6

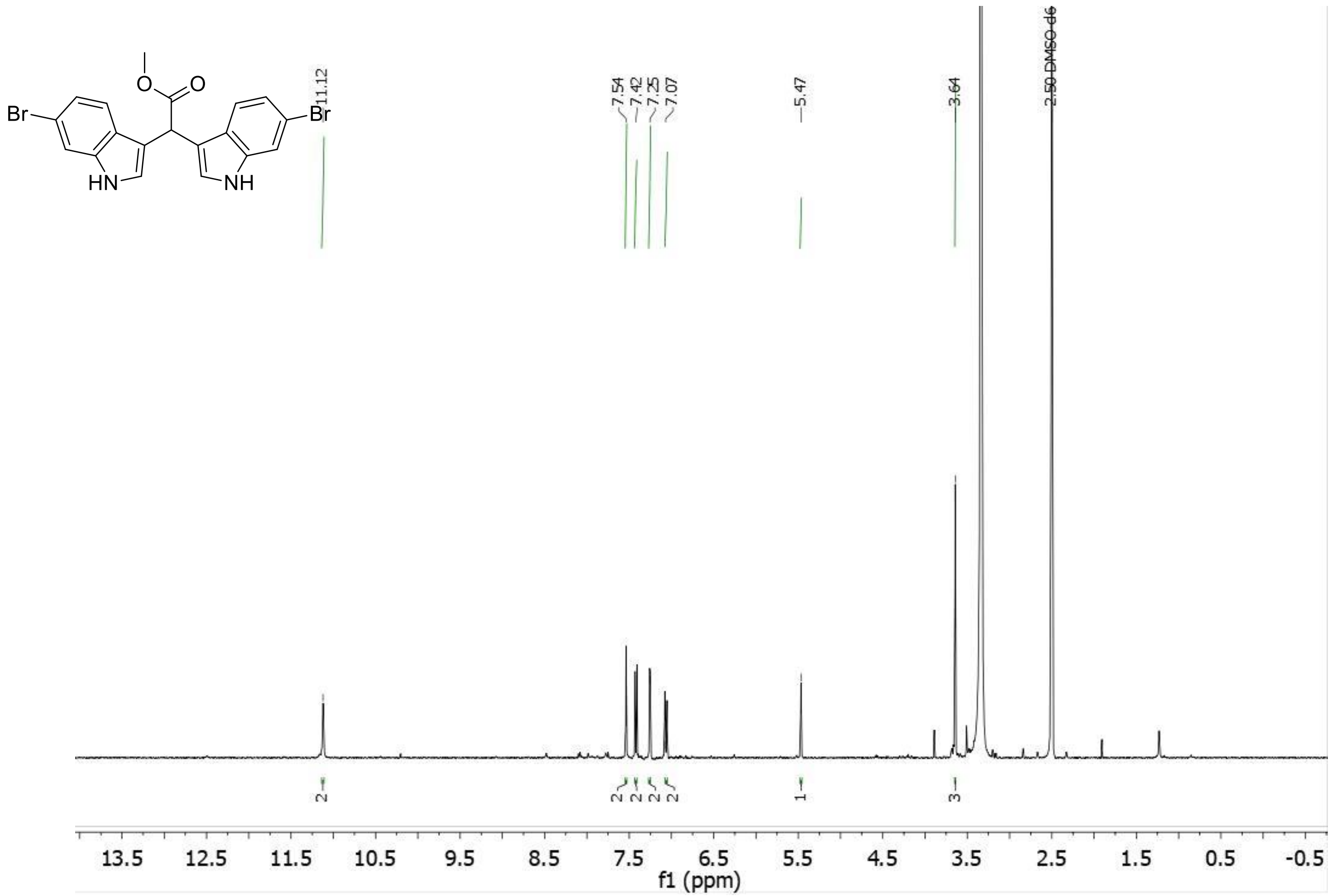

Figure S13. ^{1}H NMR spectrum (500 MHz) of methyl 2,2-bis(6-bromo-1*H* -indol-3-yl)acetate (**14**) in DMSO-d_6

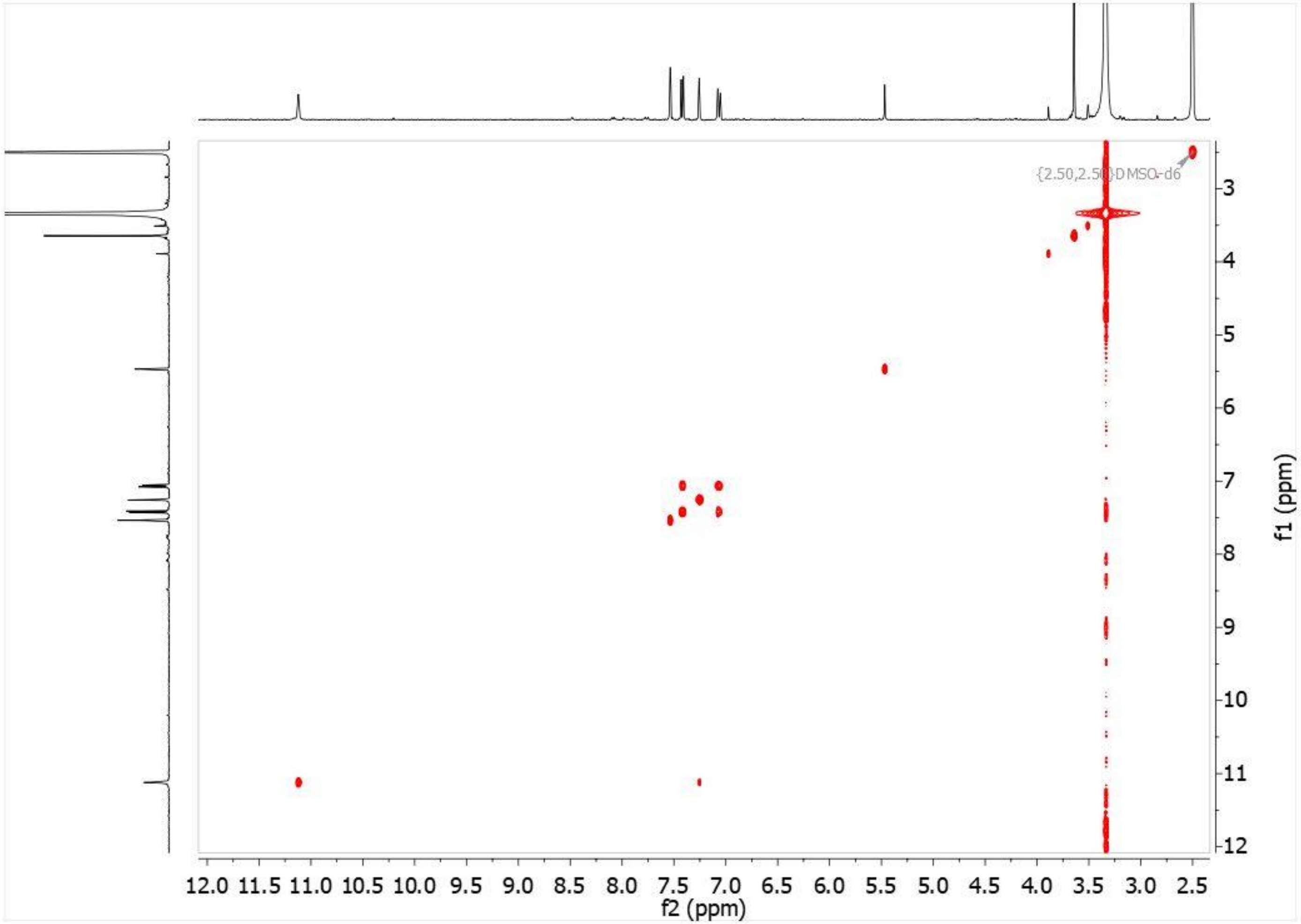

Figure S14. COSY spectrum (500 MHz) of methyl 2,2-bis(6-bromo-1*H* -indol-3-yl)acetate (**14**) in DMSO-d_6

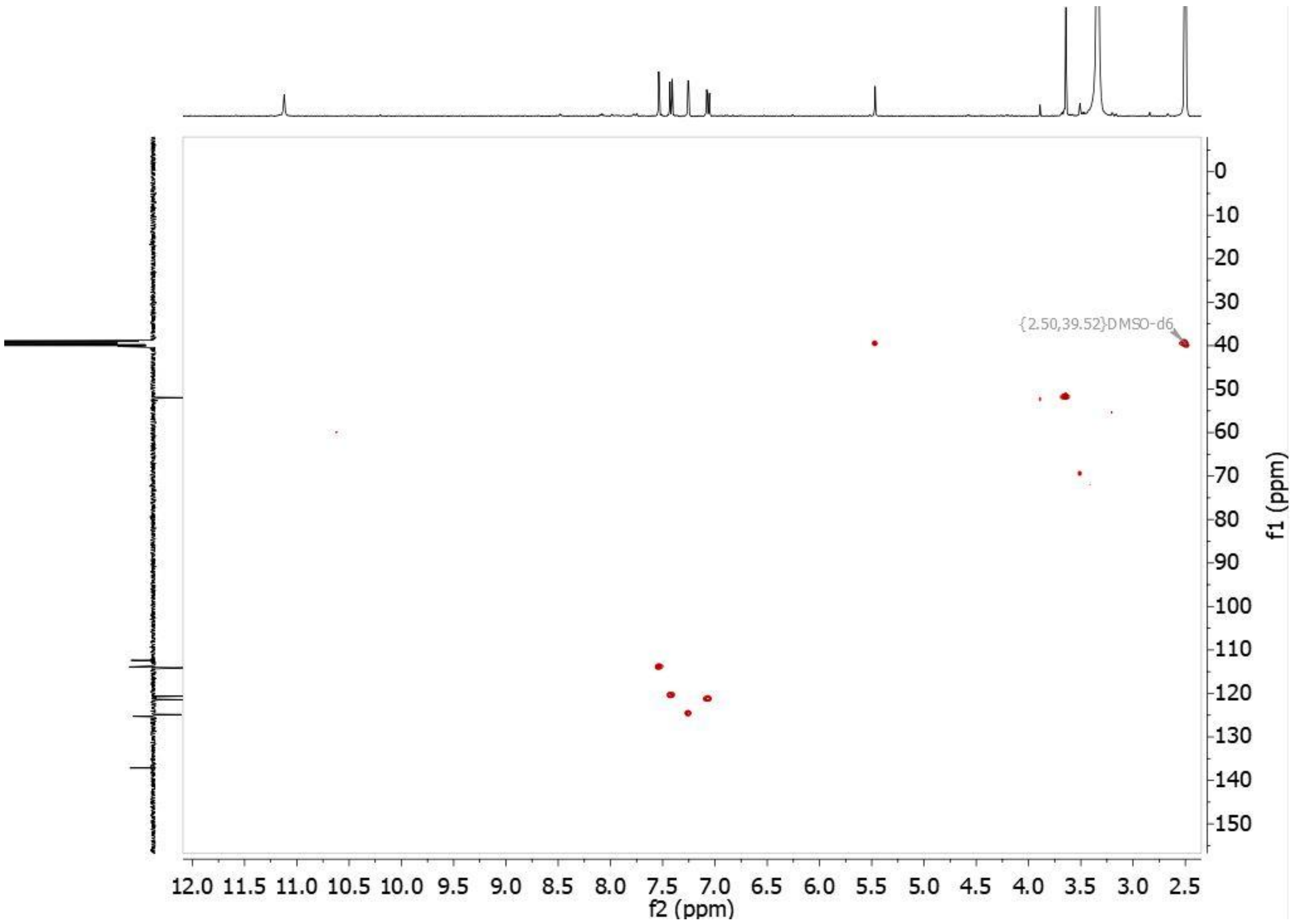

Figure S15. HSQC spectrum (500 MHz) of methyl 2,2-bis(6-bromo-1*H* -indol-3-yl)acetate (**14**) in DMSO-d_6

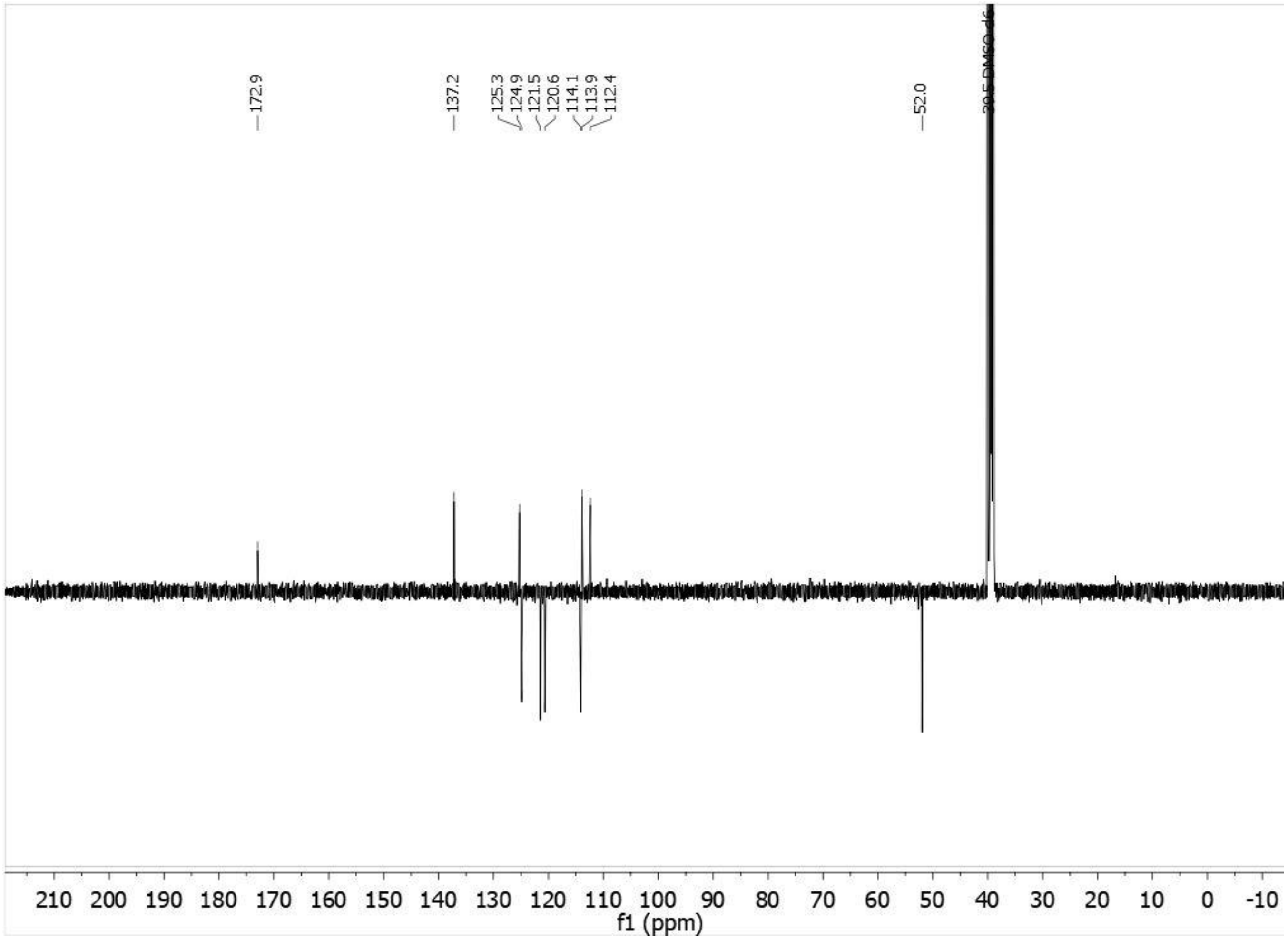

Figure S16. ^{13}C NMR spectrum (125 MHz) of methyl 2,2-bis(6-bromo-1*H* -indol-3-yl)acetate (**14**) in DMSO-d_6

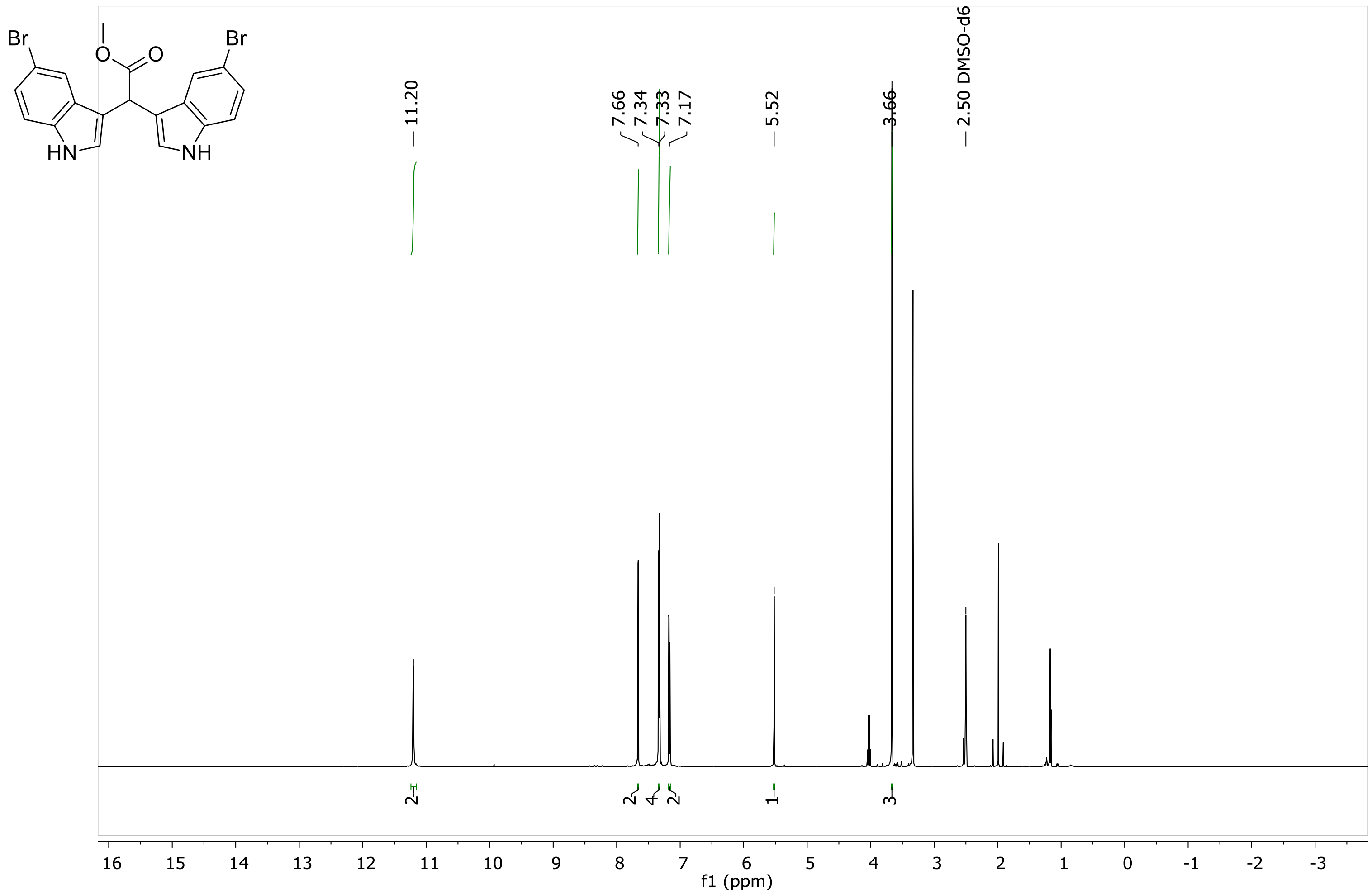

Figure S17. ^{1}H NMR spectrum (500 MHz) of methyl 2,2-bis(5-bromo-1*H* -indol-3-yl)acetate (**15**) in DMSO-d_6

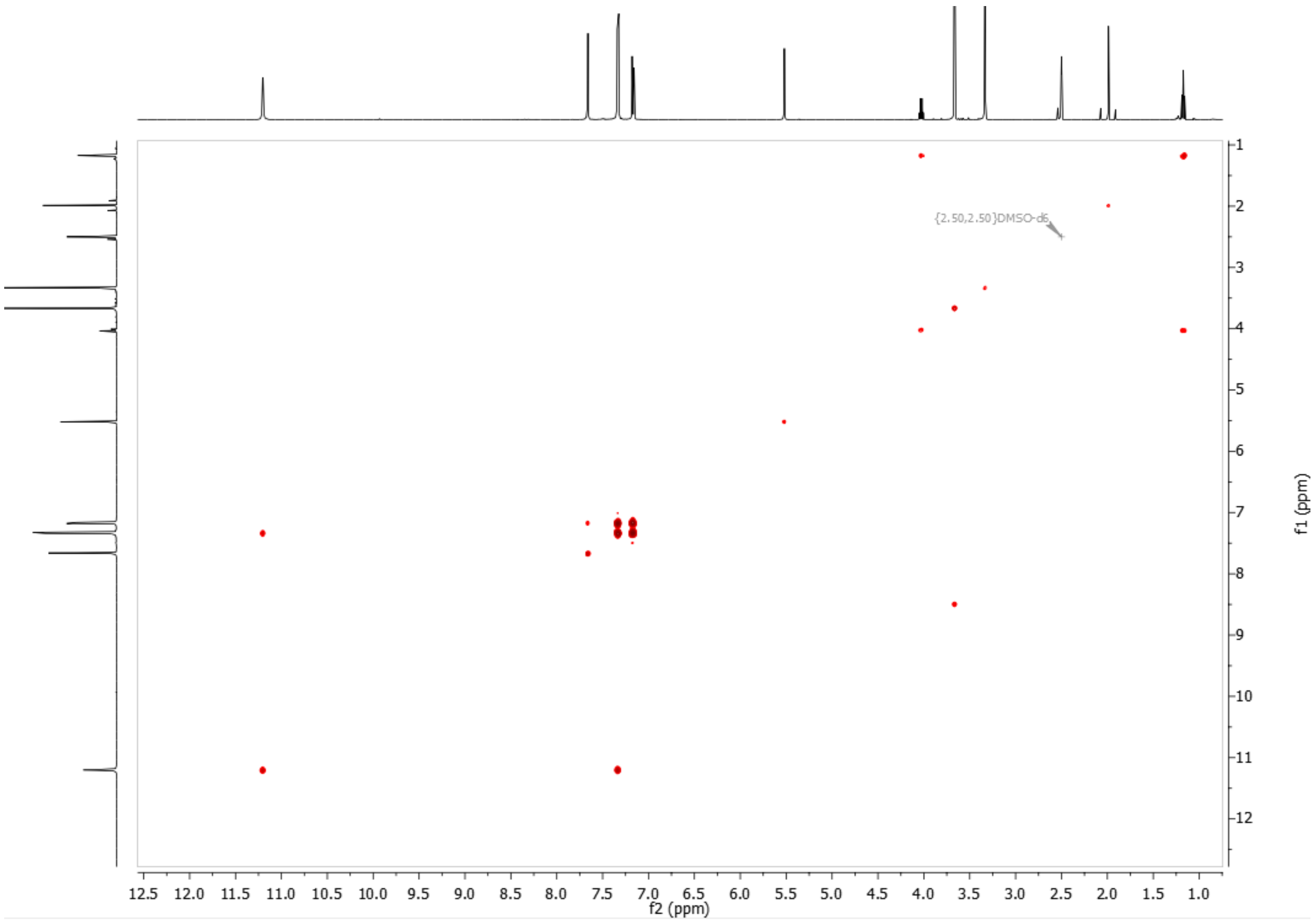

Figure S18. COSY NMR spectrum (500 MHz) of methyl 2,2-bis(5-bromo-1*H* -indol-3-yl)acetate (**15**) in DMSO-d_6

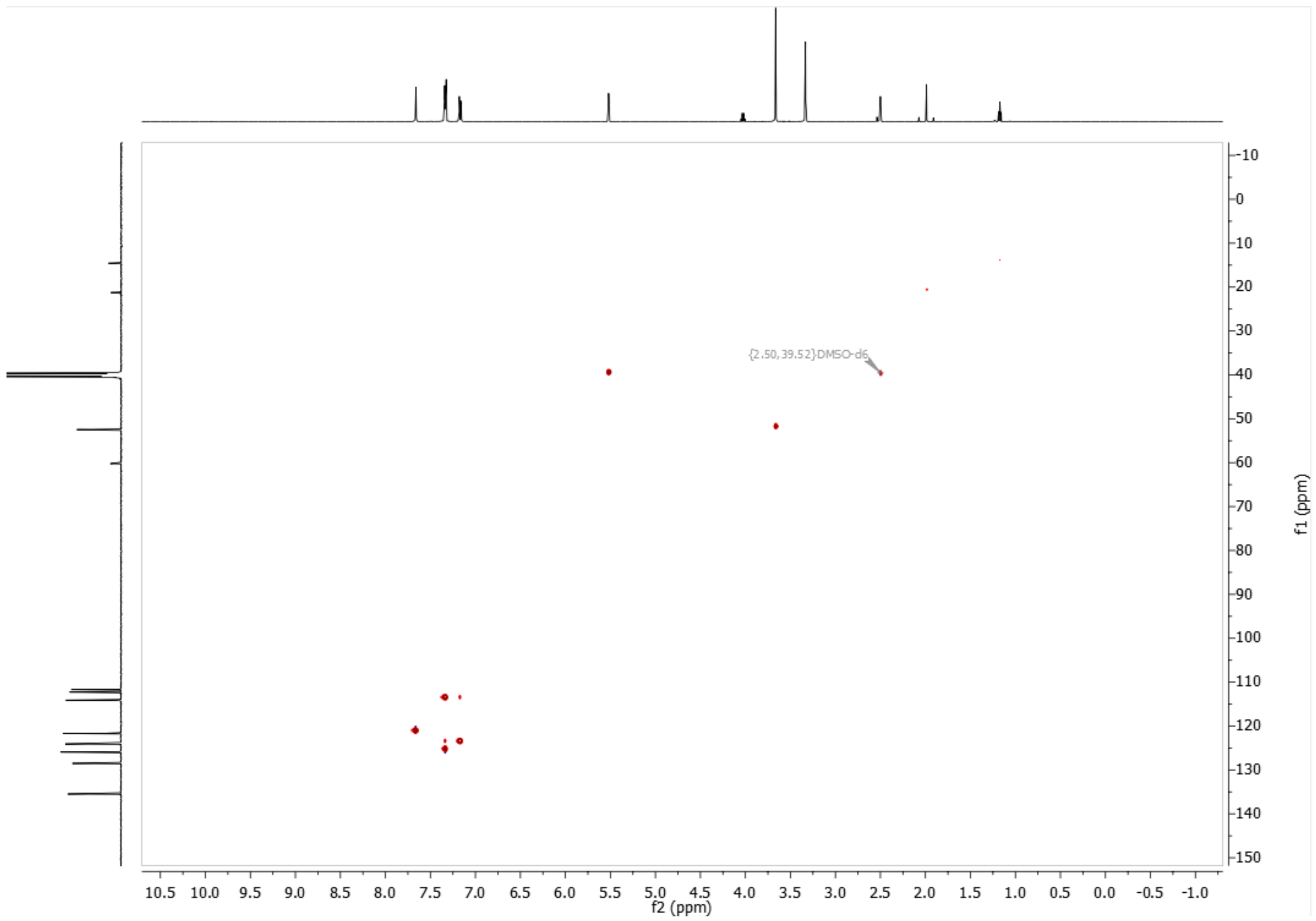

Figure S19. HSQC NMR spectrum (500 MHz) of methyl 2,2-bis(5-bromo-1*H* -indol-3-yl)acetate (**15**) in DMSO-d_6

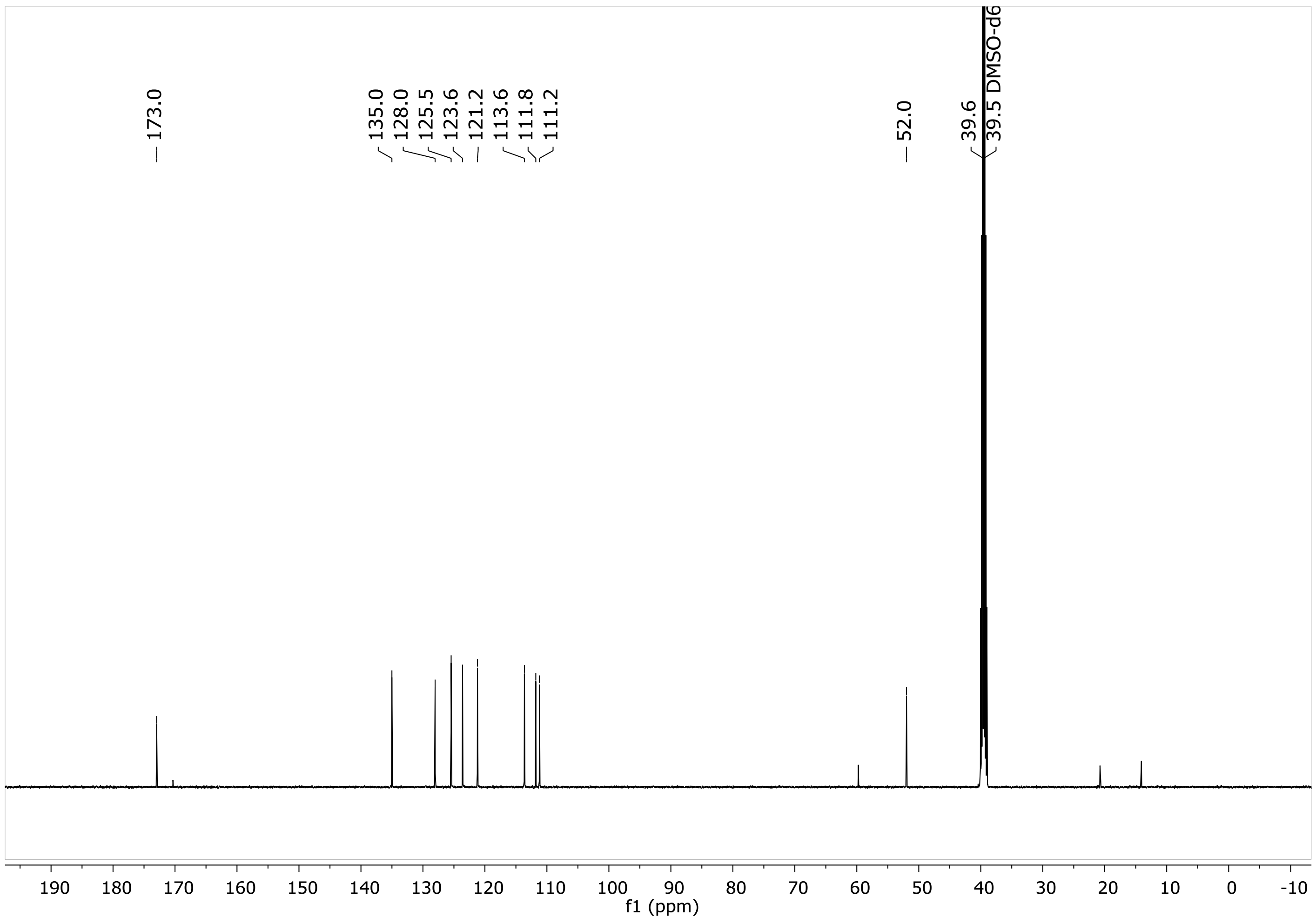

Figure S20. ^{13}C NMR spectrum (125 MHz) of methyl 2,2-bis(5-bromo-1*H* -indol-3-yl)acetate (**15**) in DMSO-d_6

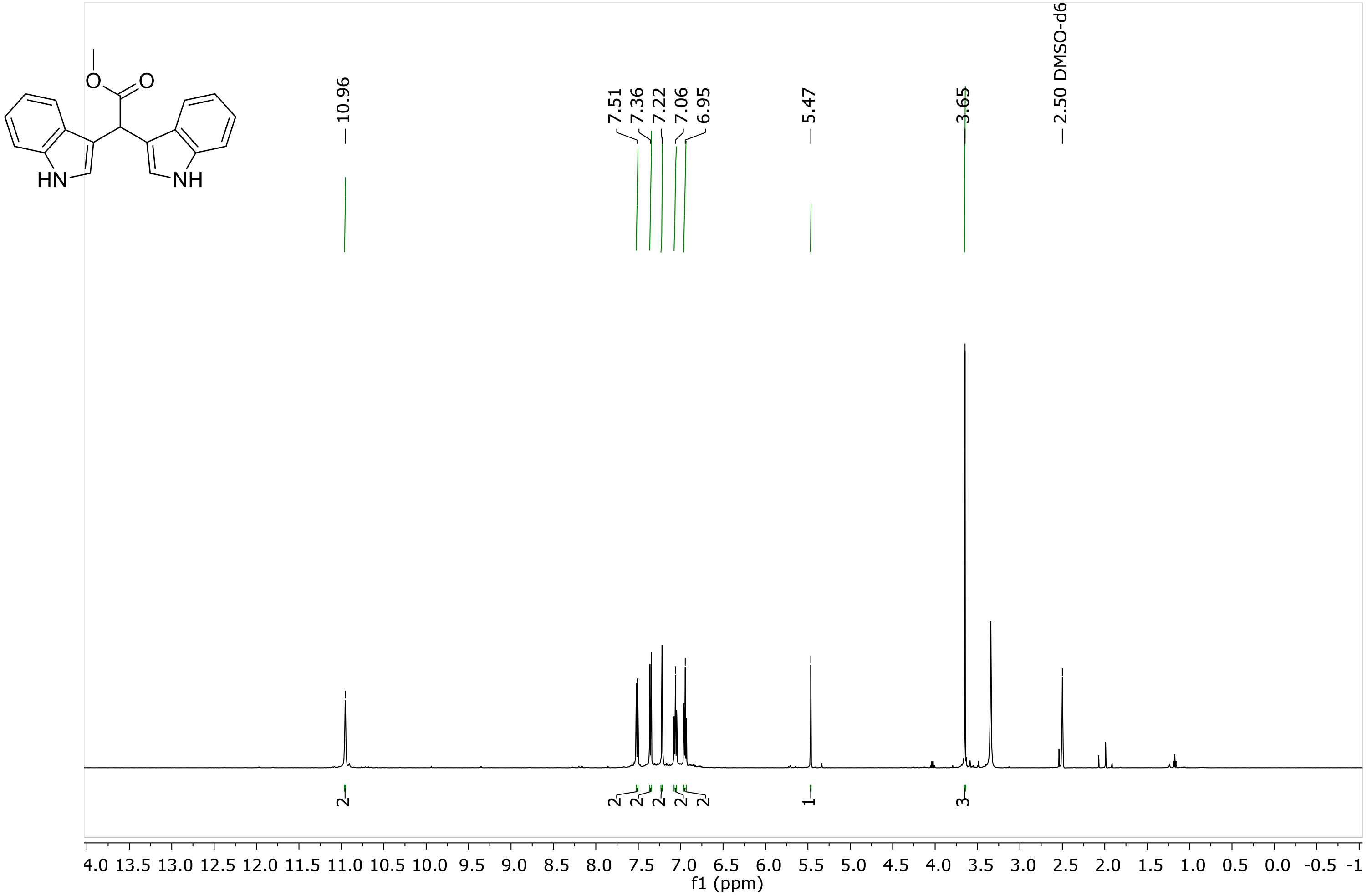

Figure S21. ^{1}H NMR spectrum (500 MHz) of methyl 2,2-di(1*H*-indol-3-yl)acetate (**16**) in DMSO-d_6

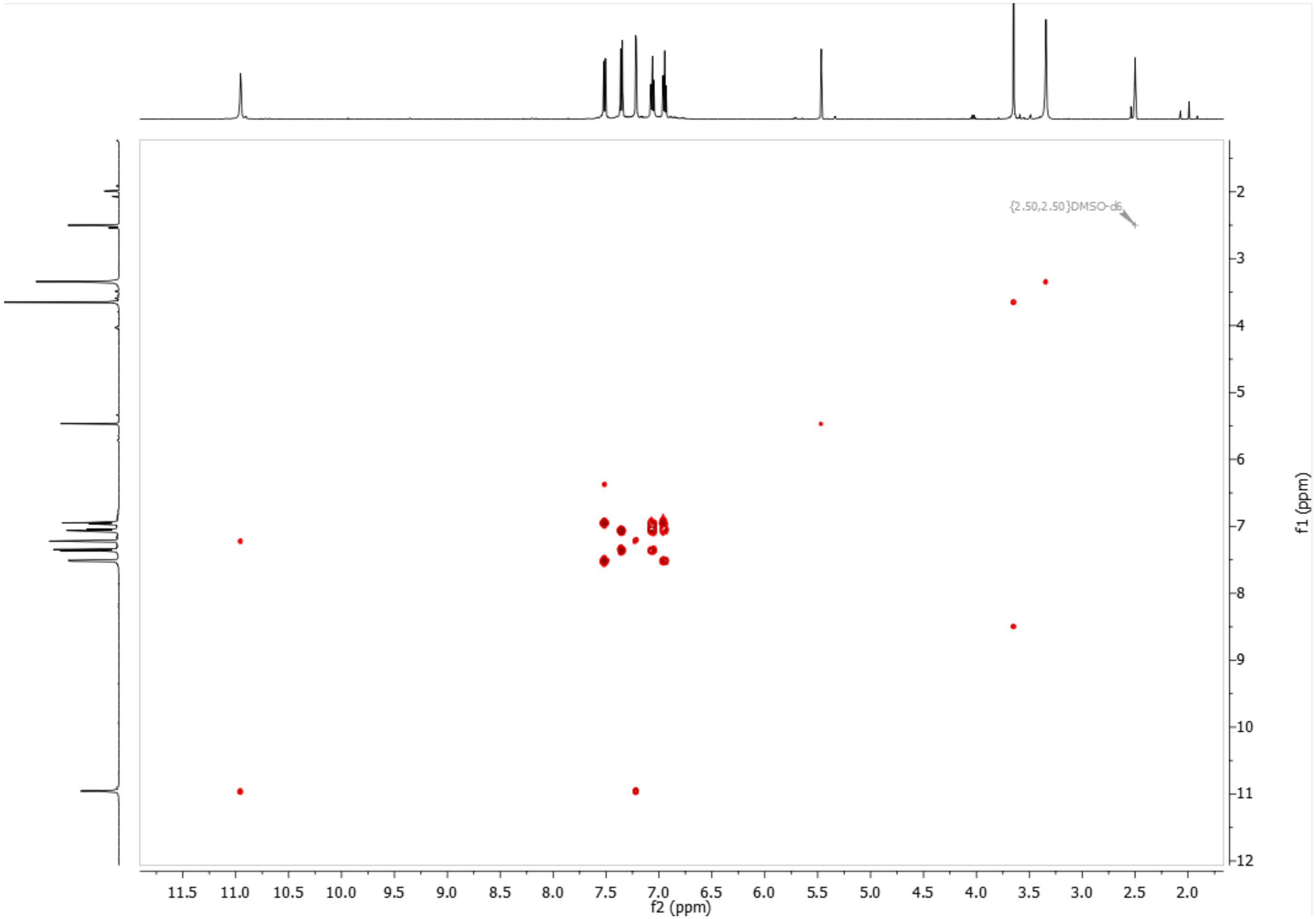

Figure S22. COSY NMR spectrum (500 MHz) of methyl 2,2-di(1*H*-indol-3-yl)acetate (**16**) in DMSO-d_6

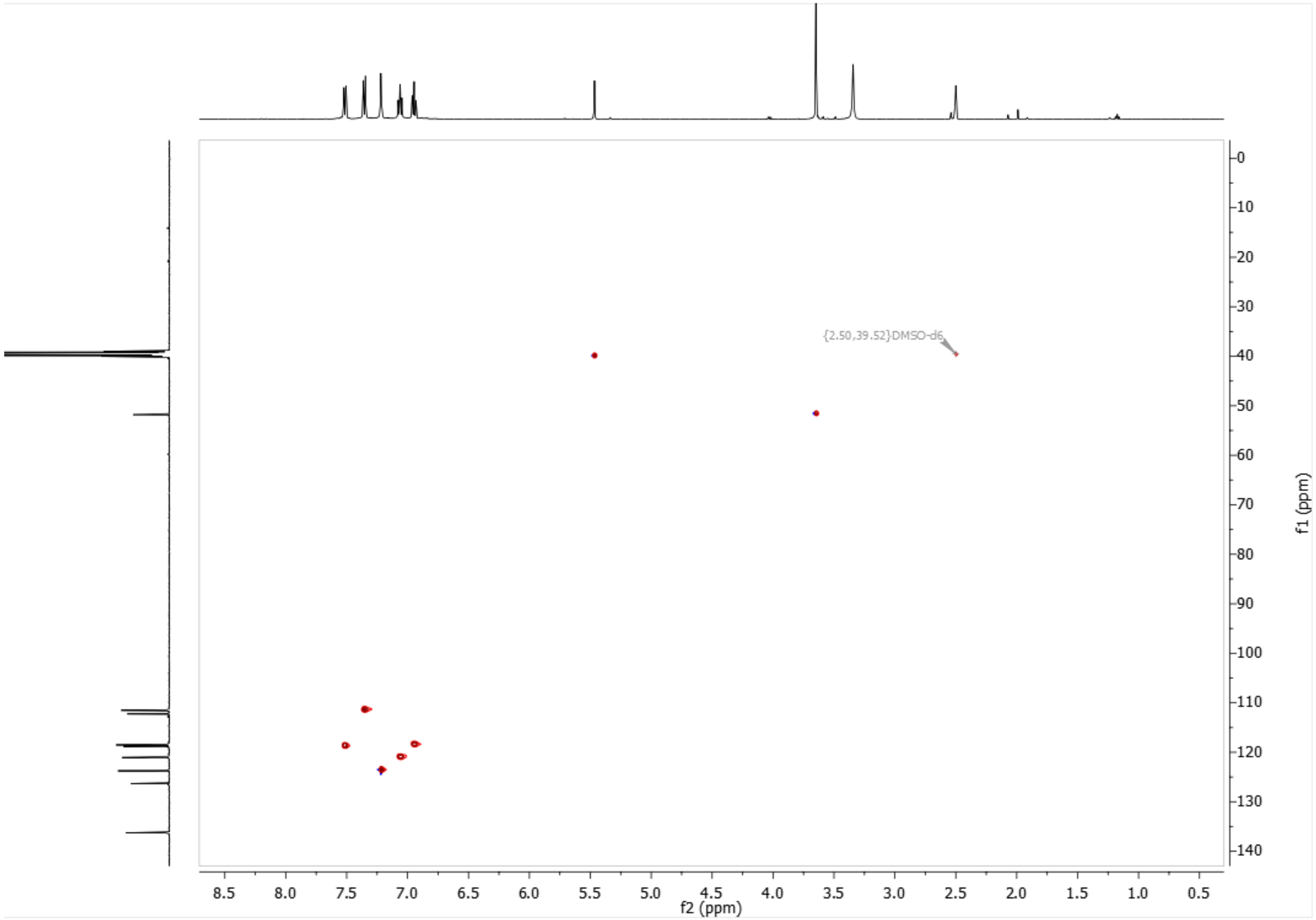

Figure S23. HSQC NMR spectrum (500 MHz) of methyl 2,2-di(1*H*-indol-3-yl)acetate (**16**) in DMSO-d_6

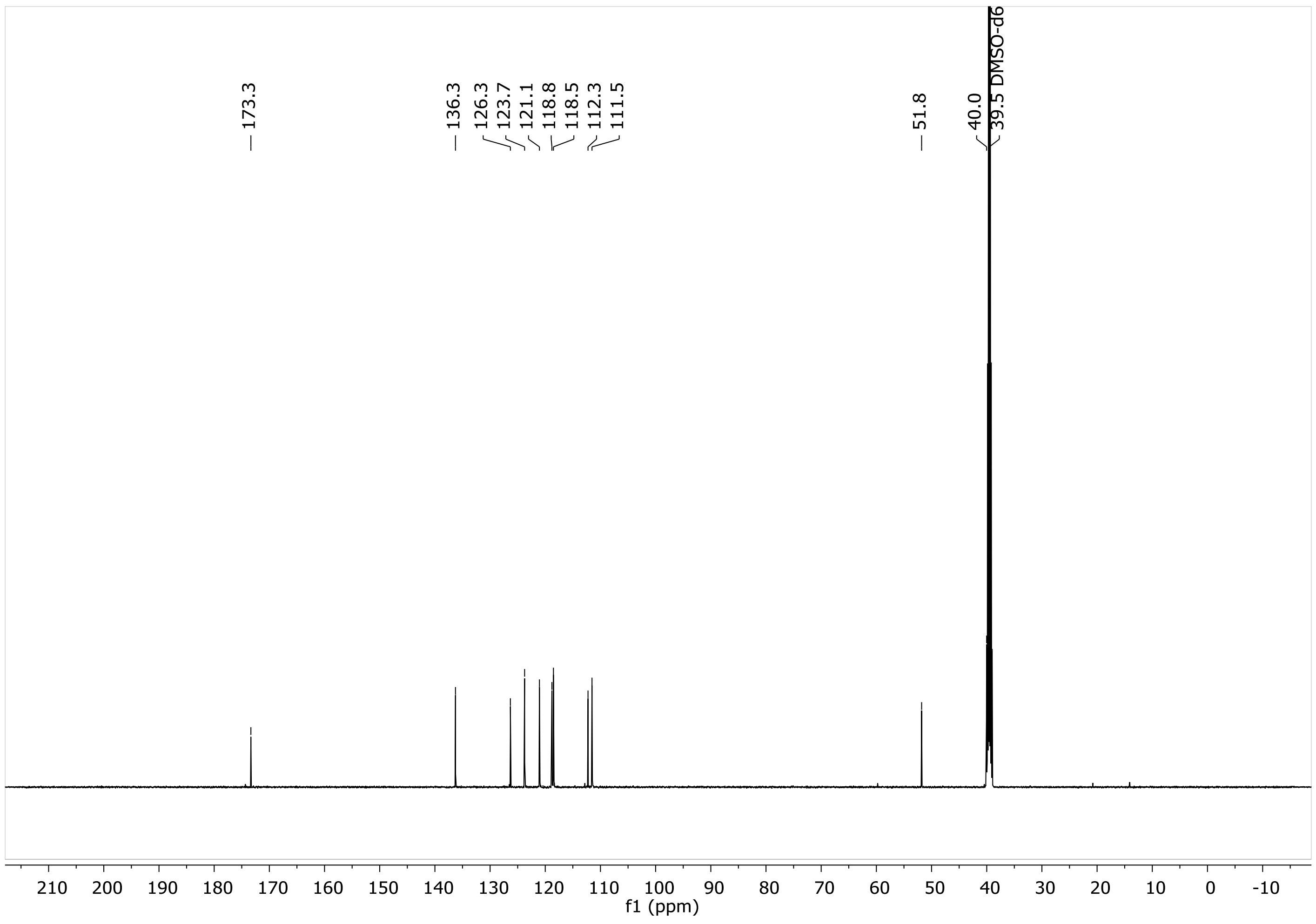

Figure S24. ^{13}C NMR spectrum (125 MHz) of methyl 2,2-di(1*H*-indol-3-yl)acetate (**16**) in DMSO-d_6

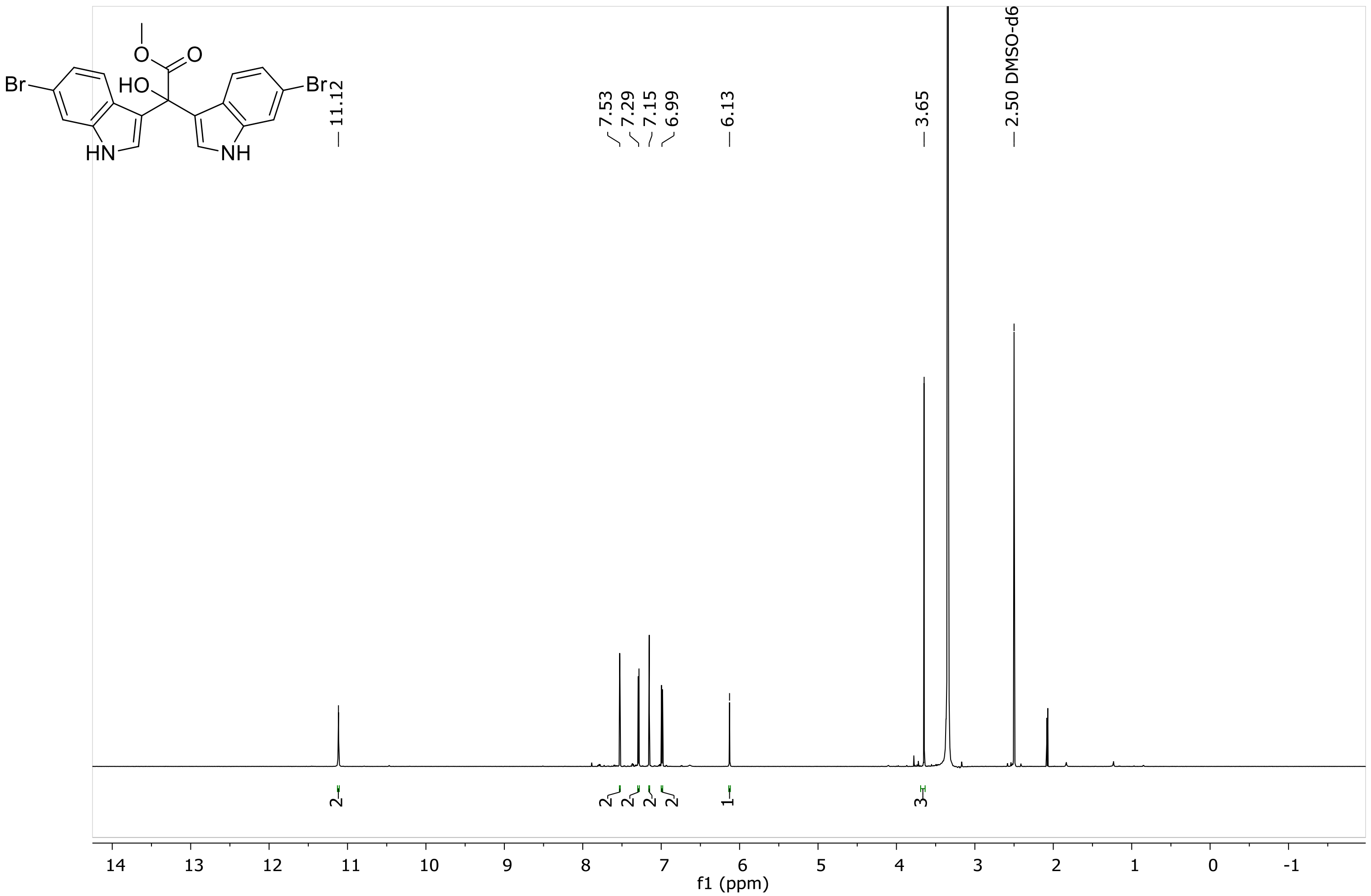

Figure S25. ^{1}H NMR spectrum (800 MHz) of methyl 2,2-bis(6-bromo-1*H* -indol-3-yl)-2-hydroxyacetate (**17**) in DMSO-d_6

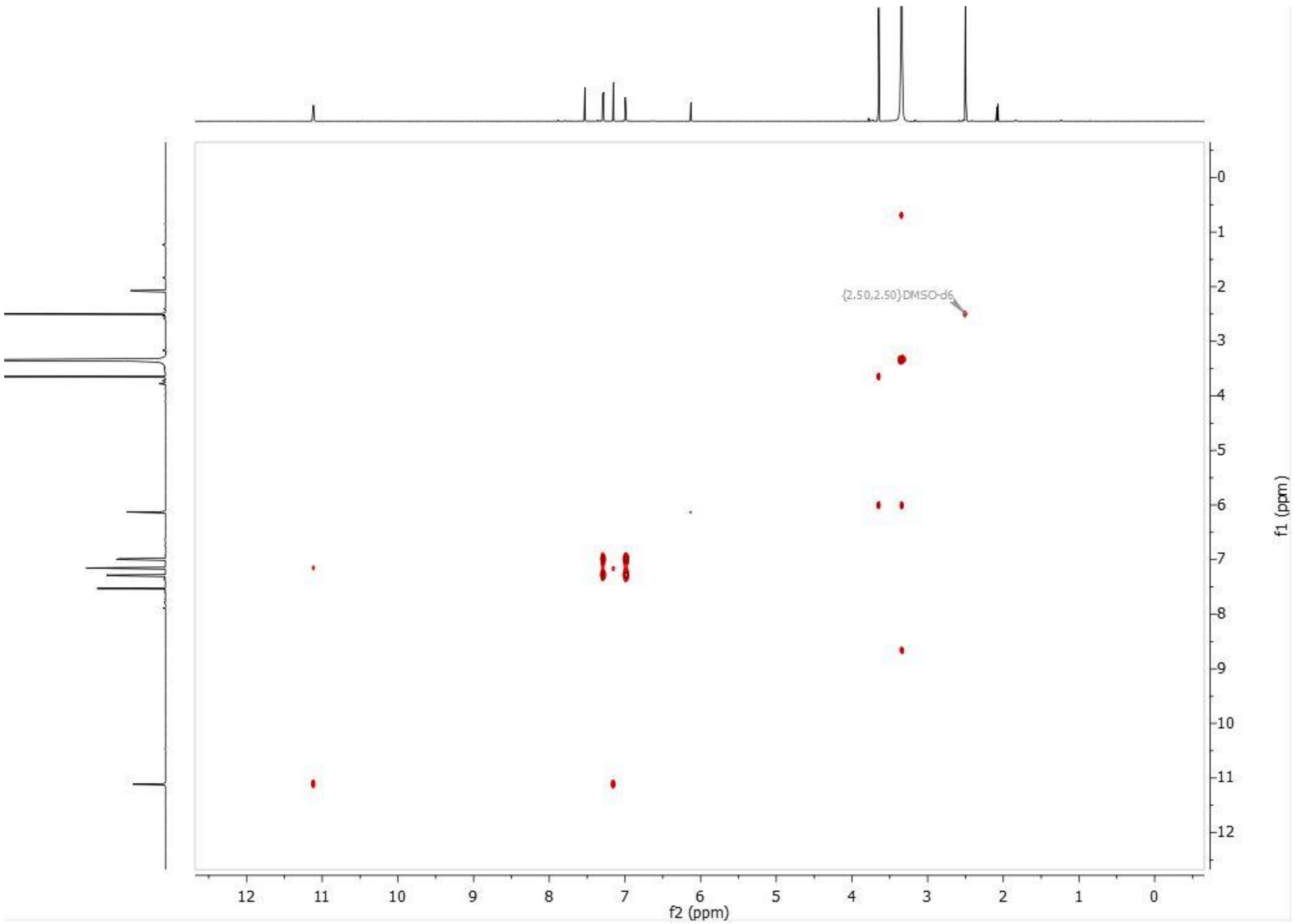

Figure S26. COSY NMR spectrum (800 MHz) of methyl 2,2-bis(6-bromo-1*H* -indol-3-yl)-2-hydroxyacetate (**17**) in DMSO-d_6

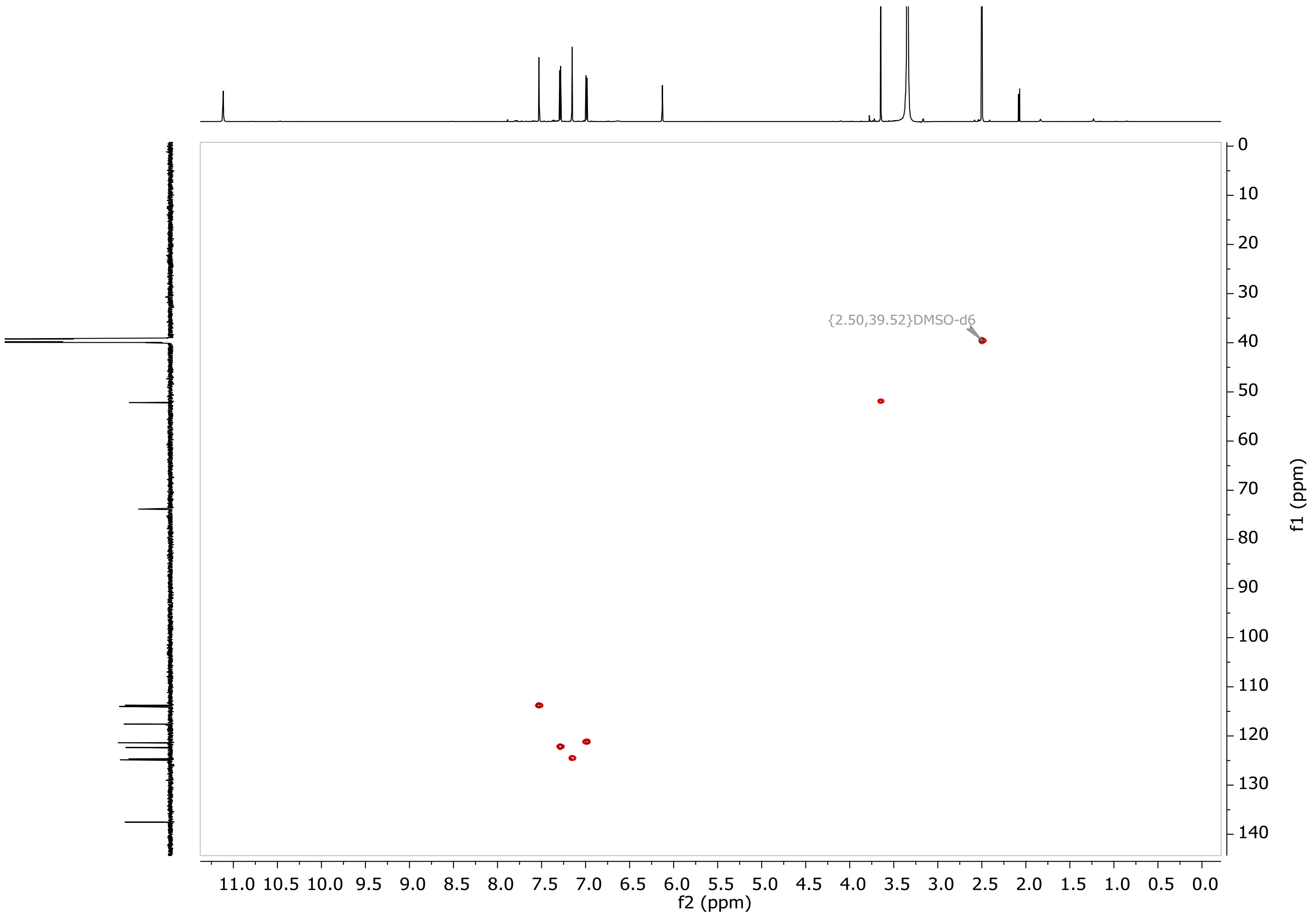

Figure S27. HSQC spectrum (500 MHz) of methyl 2,2-bis(6-bromo-1*H* -indol-3-yl)-2-hydroxyacetate (**17**) in DMSO-d_6

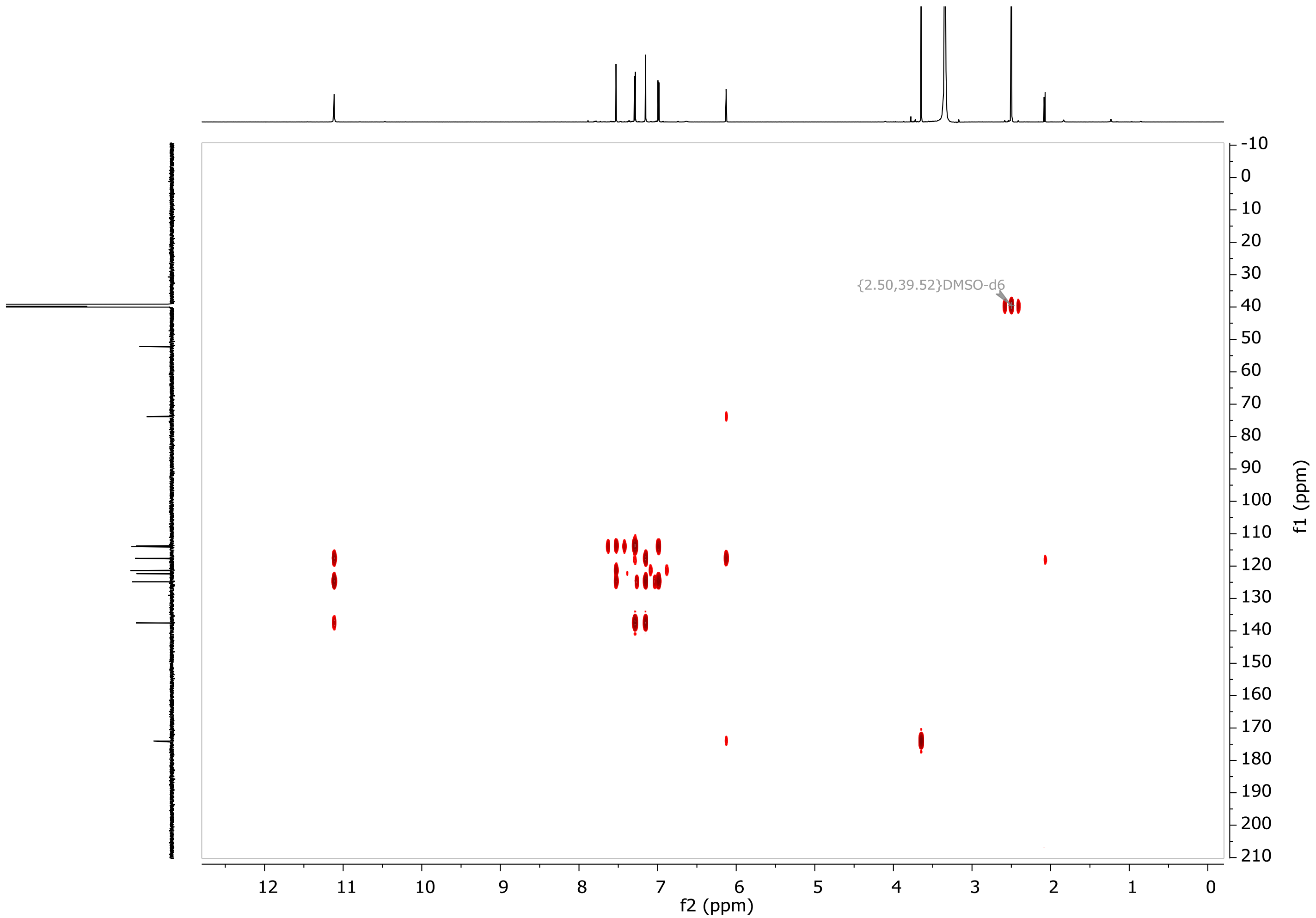

Figure S28. HMBC spectrum (500 MHz) of methyl 2,2-bis(6-bromo-1*H* -indol-3-yl)-2-hydroxyacetate (**17**) in DMSO-d_6

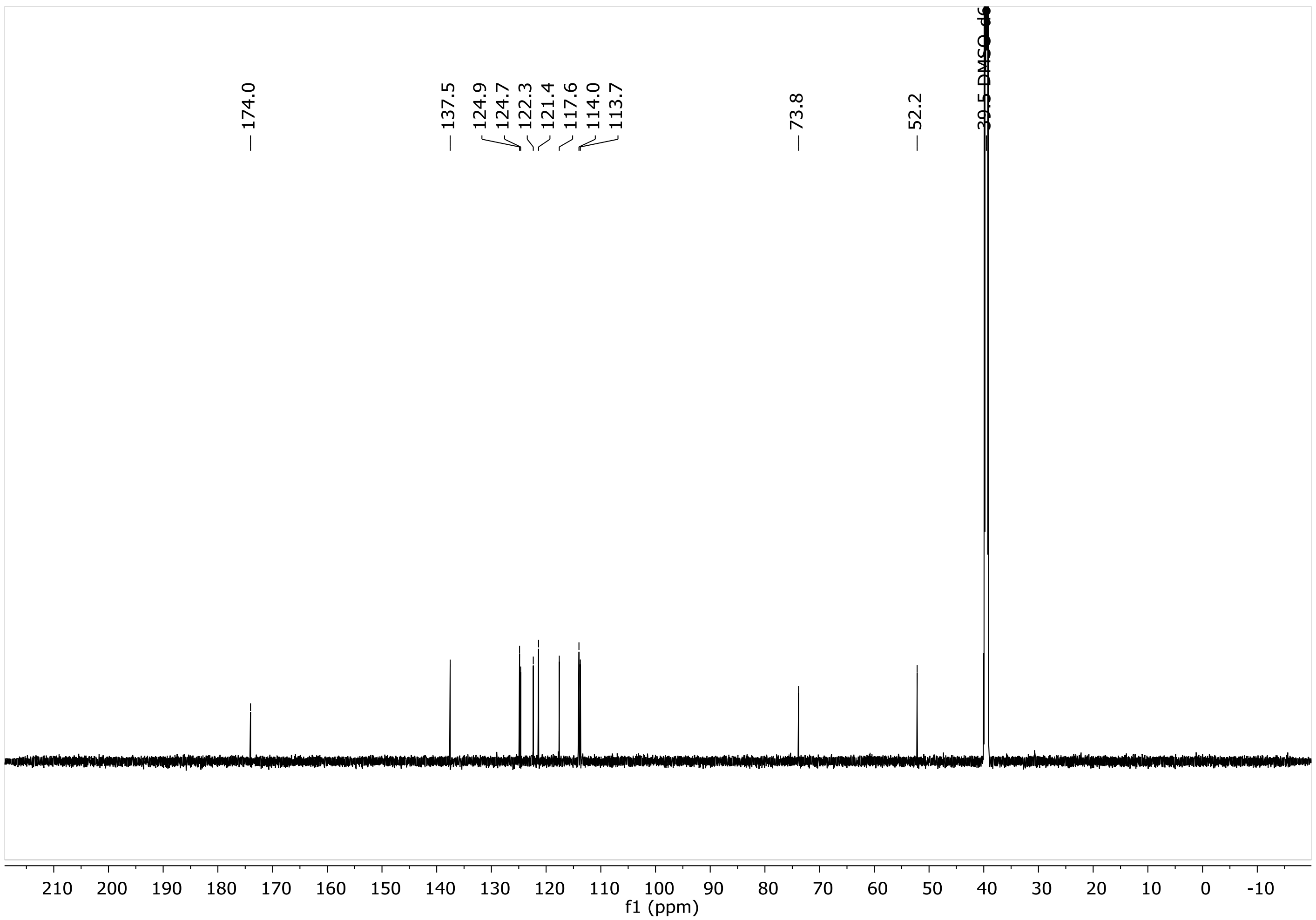

Figure S29. ^{13}C NMR spectrum (200 MHz) of methyl 2,2-bis(6-bromo-1*H* -indol-3-yl)-2-hydroxyacetate (**17**) in DMSO-d_6

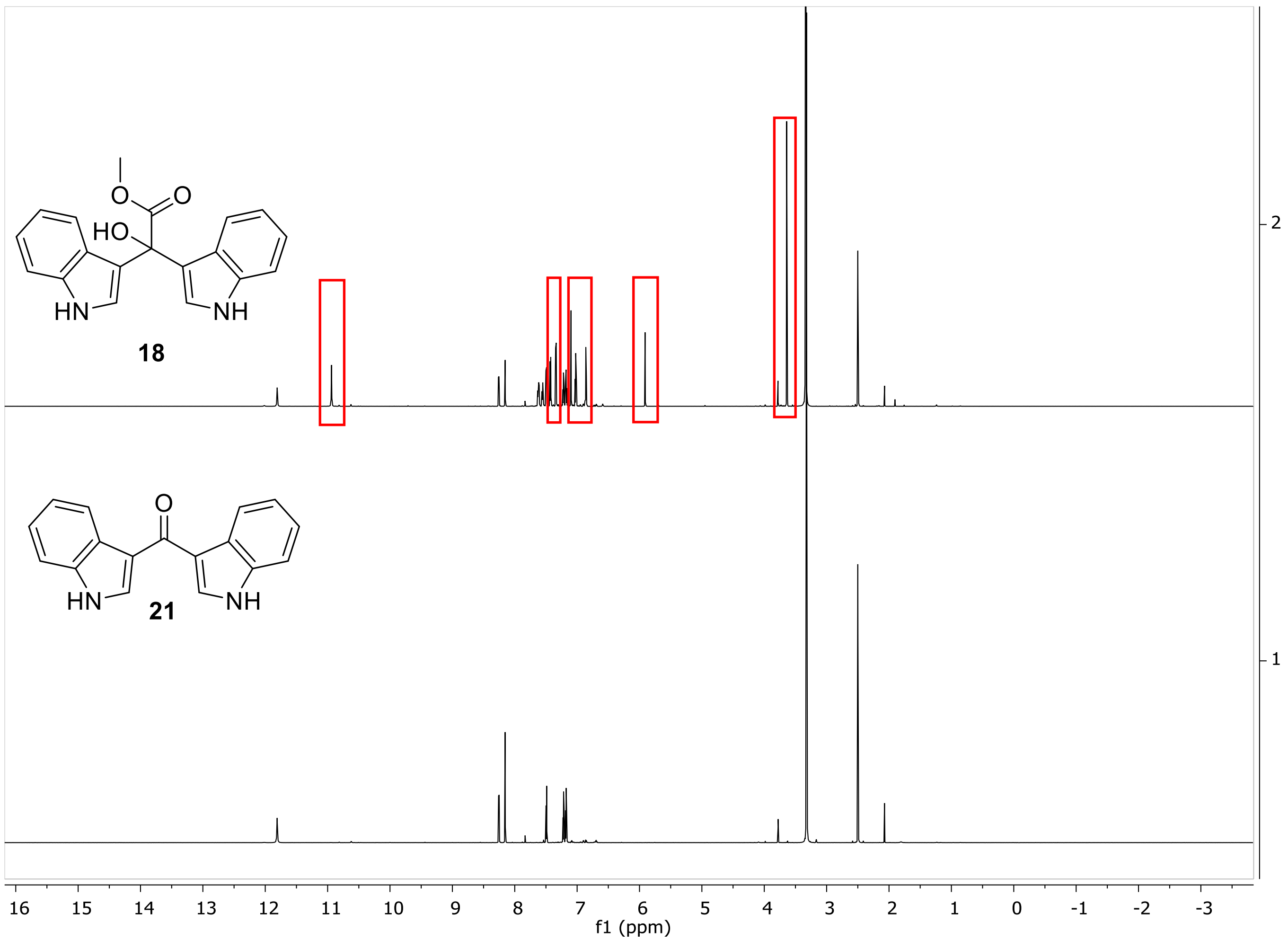

Figure S30. ^{1}H NMR spectrum (500 MHz) of fraction containing 2:1 methyl 2-hydroxy-2,2-di(1*H* -indol-3-yl)acetate (**18** - red highlighted) and di(1*H* -indol-3-yl)methanone (**21** - bottom spectrum) DMSO-*d*$_6$

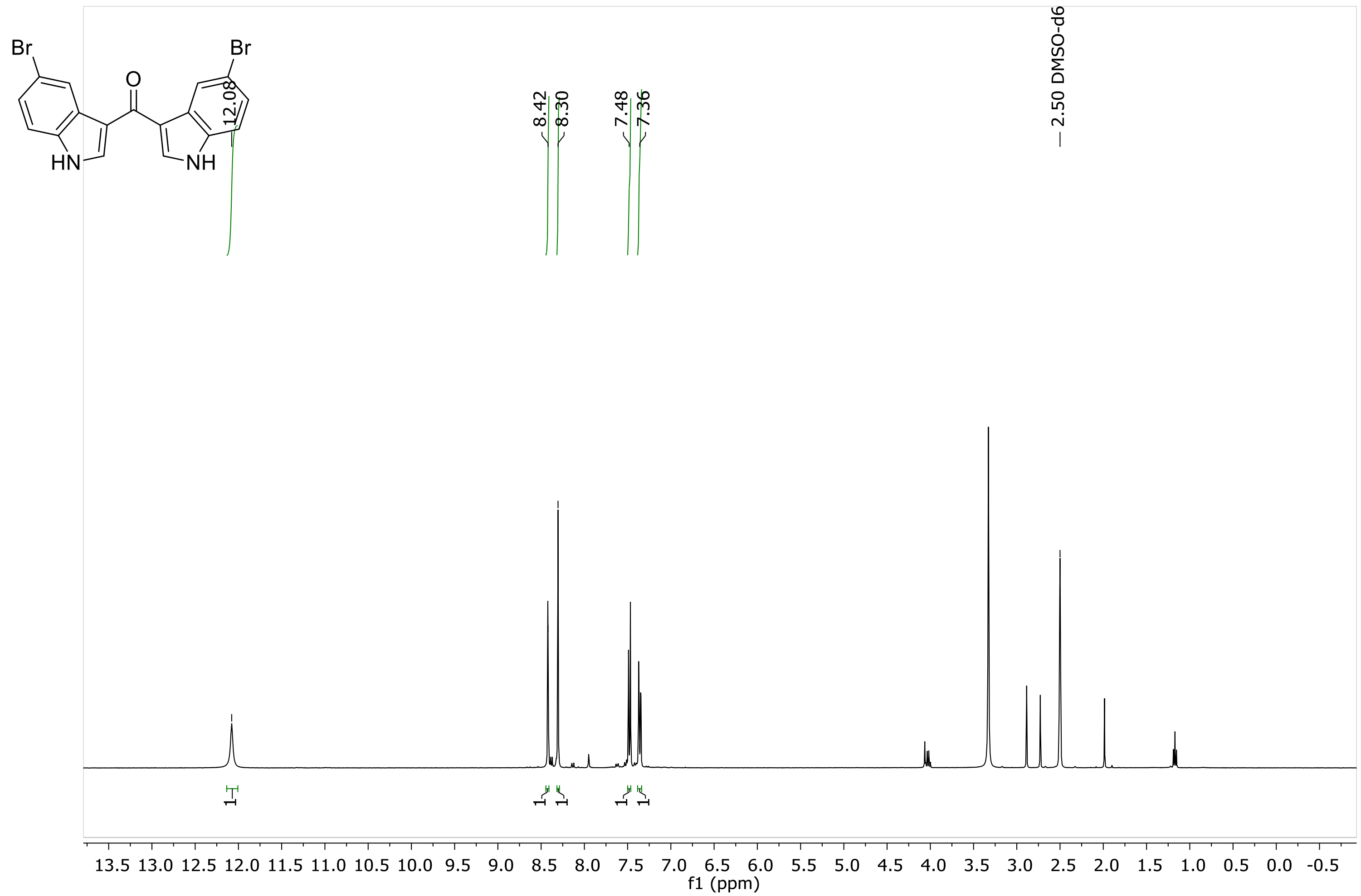

Figure S31. ^{1}H NMR spectrum (400 MHz) of bis(5-bromo-1*H* -indol-3-yl)methanone (**19**) in DMSO-d_6

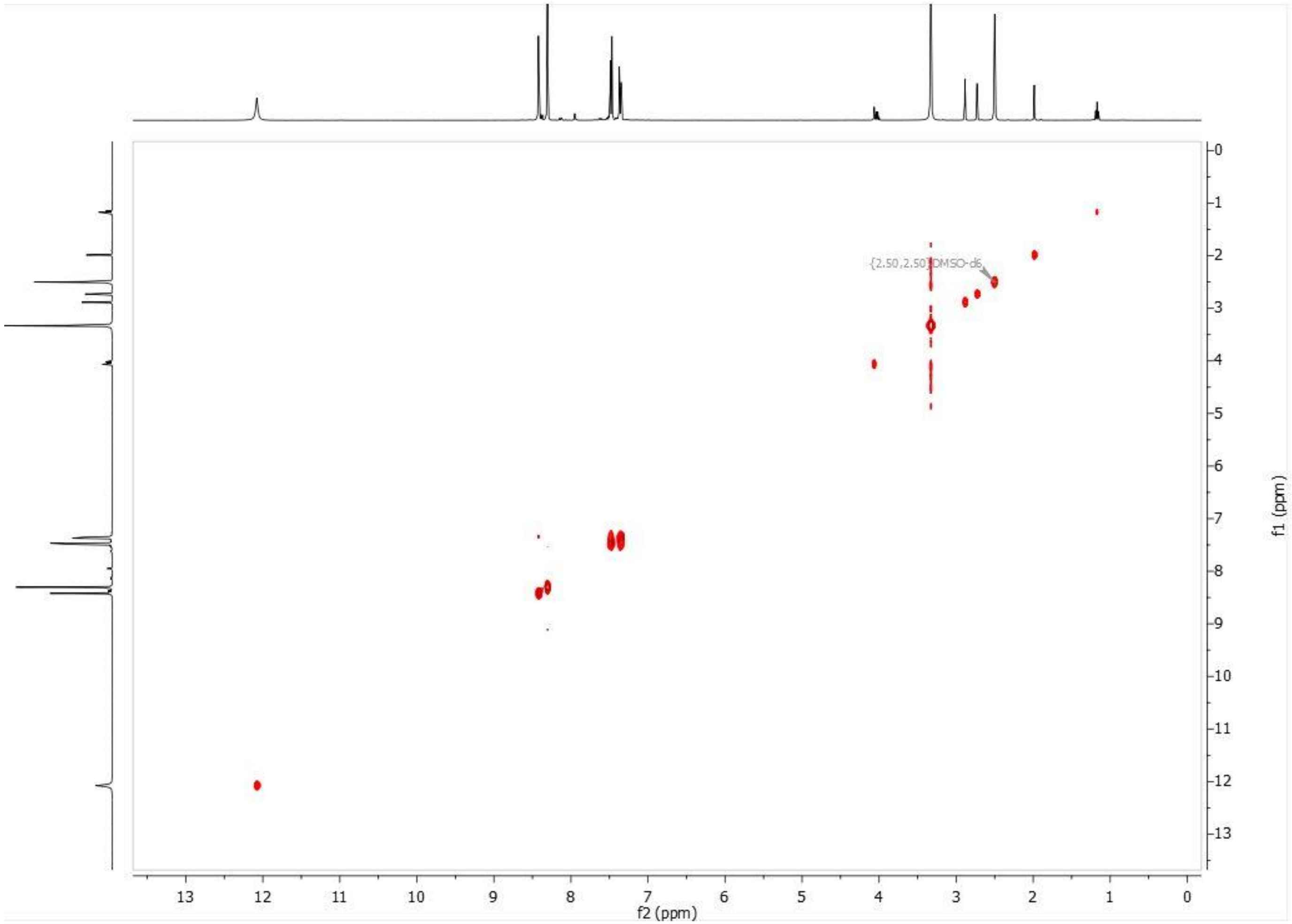

Figure S32. COSY NMR spectrum (400 MHz) of bis(5-bromo-1*H* -indol-3-yl)methanone (**19**) in DMSO-d_6

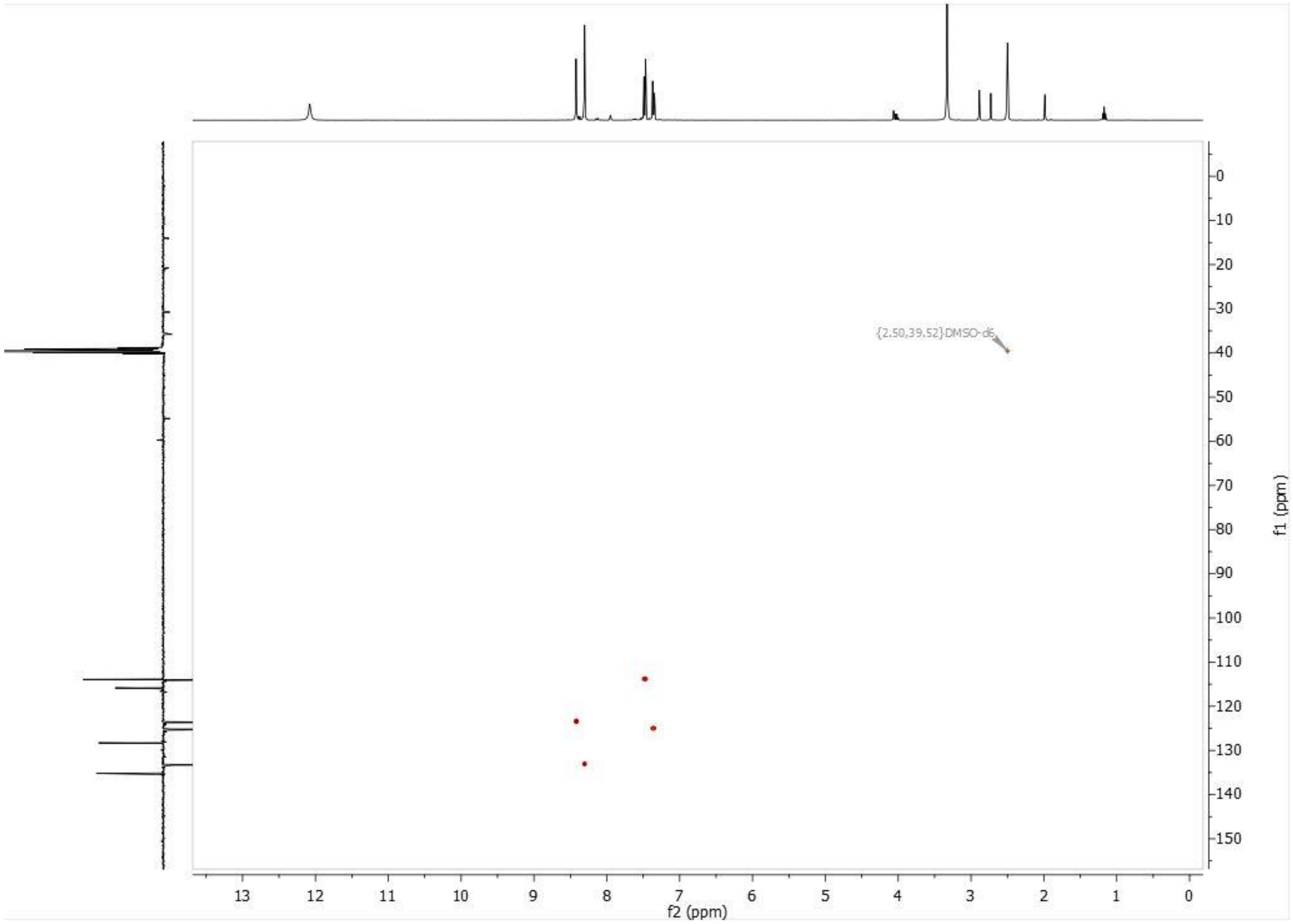

Figure S33. HSQC NMR spectrum (400 MHz) of bis(5-bromo-1*H* -indol-3-yl)methanone (**19**) in DMSO-d_6

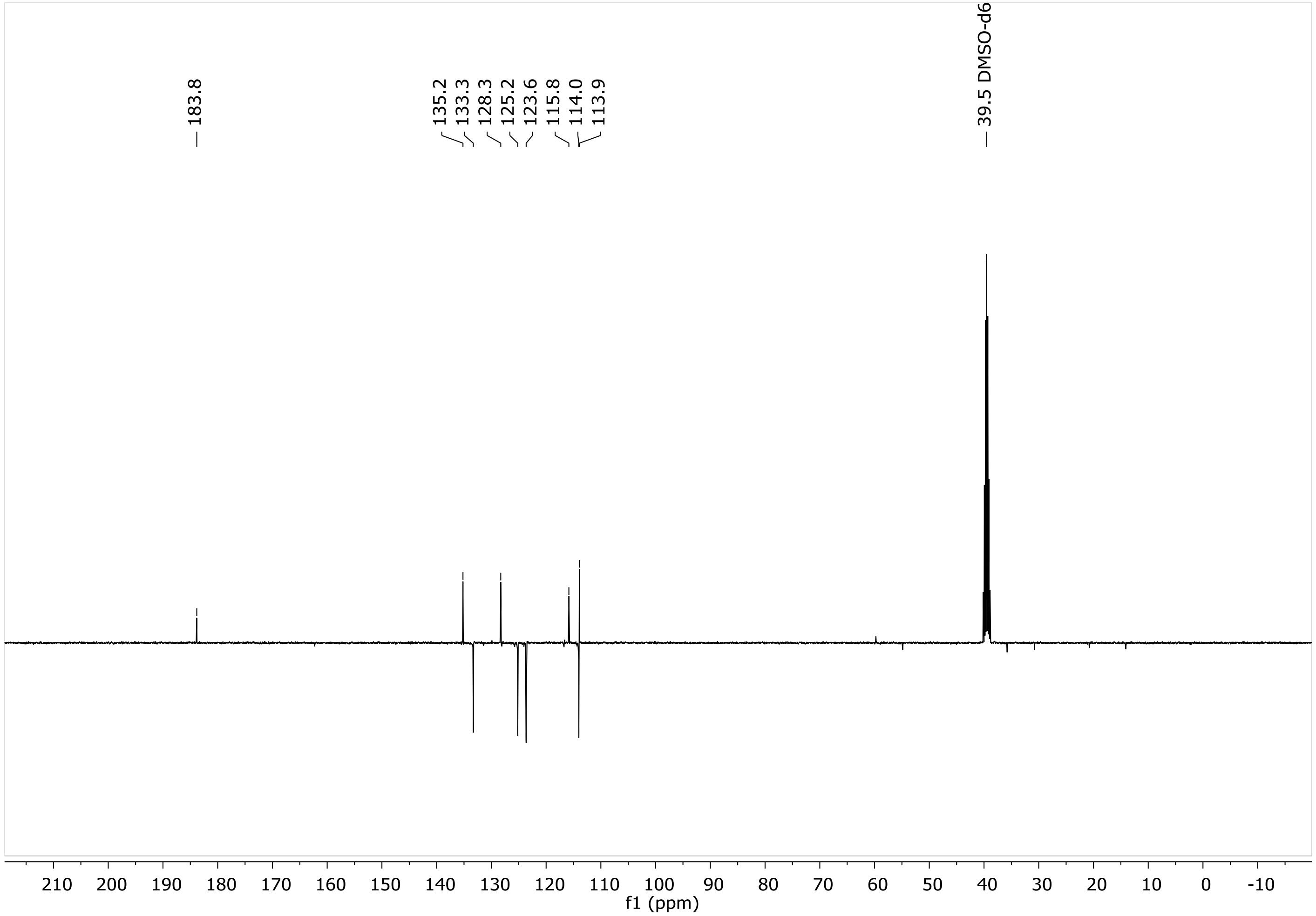

Figure S34. ^{13}C DEPT NMR spectrum (101 MHz) of bis(5-bromo-1*H* -indol-3-yl)methanone (**19**) in DMSO-d_6

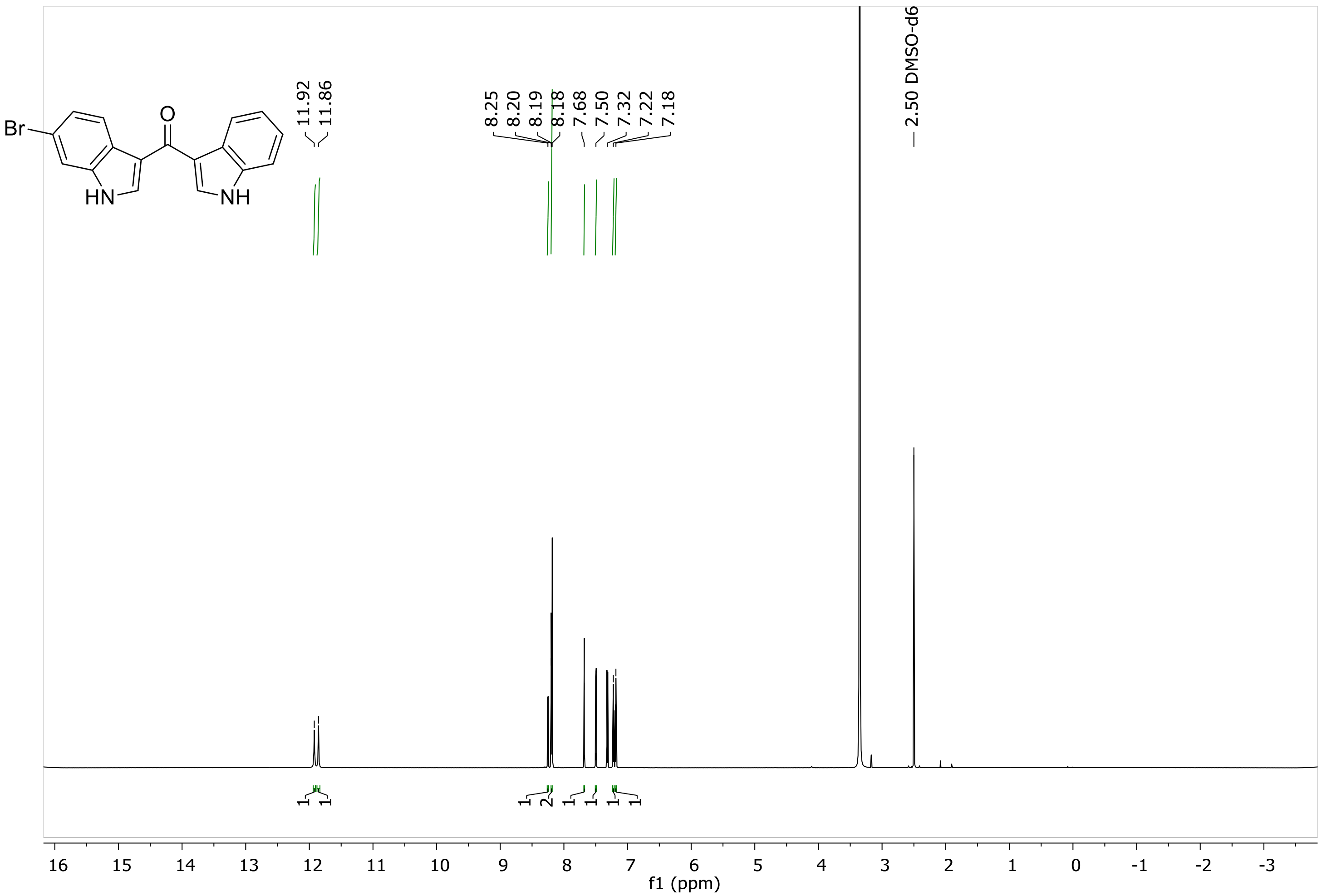

Figure S35. ^{1}H NMR spectrum (500 MHz) of (6-bromo-1*H* -indol-3-yl)(1*H* -indol-3-yl)methanone (**20**) in DMSO-d_6

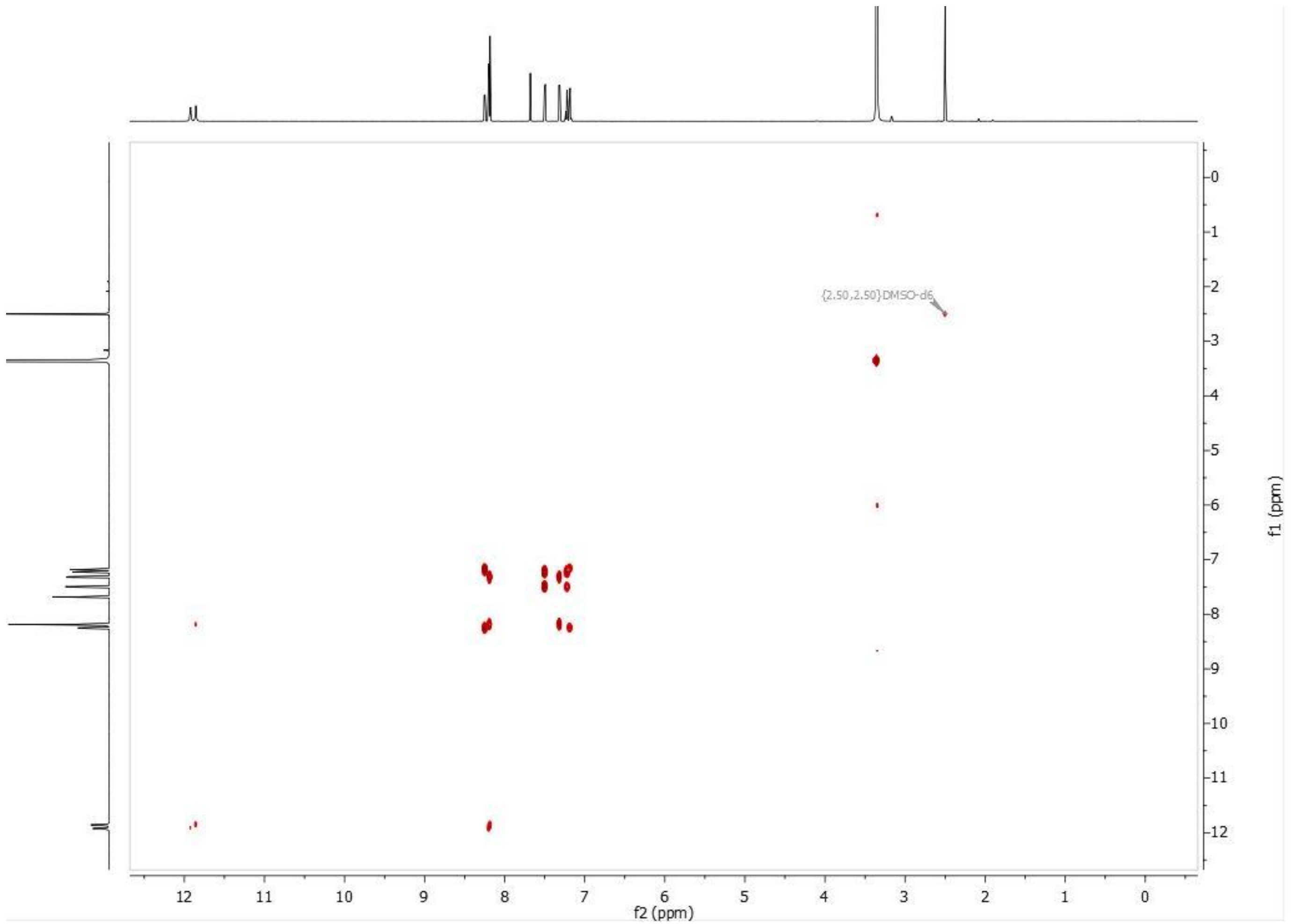

Figure S36. COSY NMR spectrum (500 MHz) of (6-bromo-1*H* -indol-3-yl)(1*H* -indol-3-yl)methanone (**20**) in DMSO-d_6

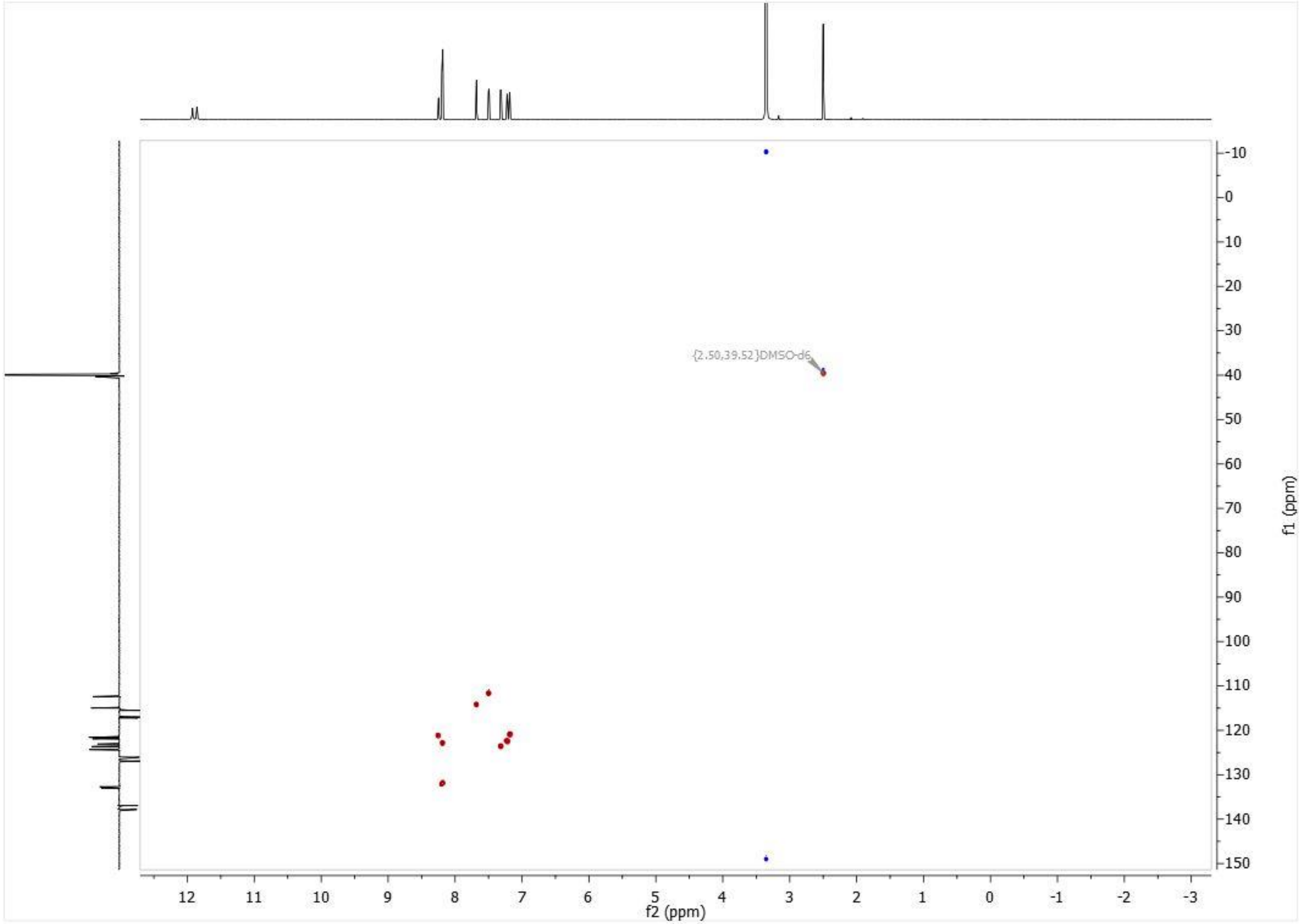

Figure S37. HSQC NMR spectrum (500 MHz) of (6-bromo-1*H* -indol-3-yl)(1*H* -indol-3-yl)methanone (**20**) in DMSO-d_6

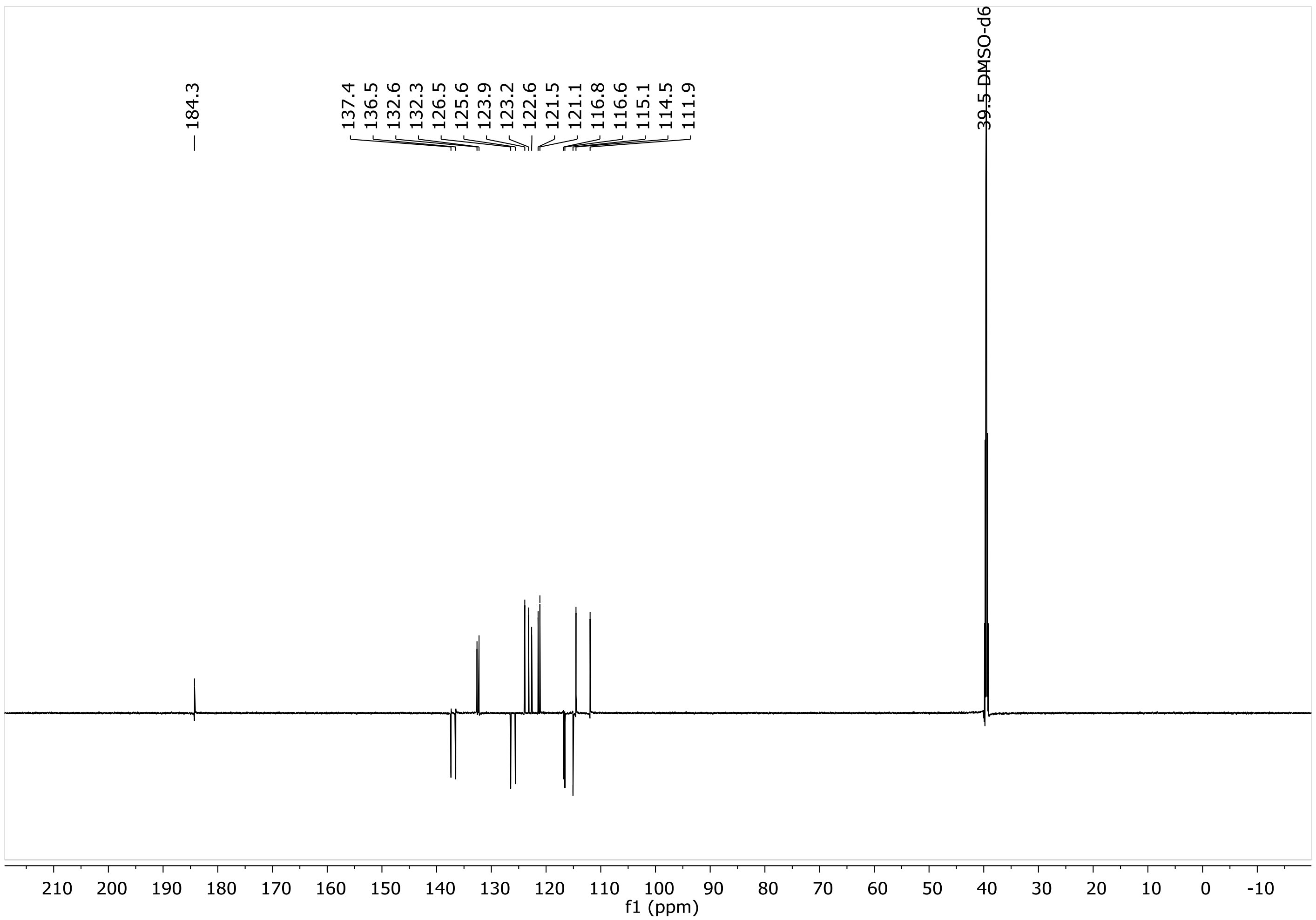

Figure S38. ^{13}C DEPT NMR spectrum (125 MHz) of (6-bromo-1*H* -indol-3-yl)(1*H* -indol-3-yl)methanone (**20**) in DMSO-d_6

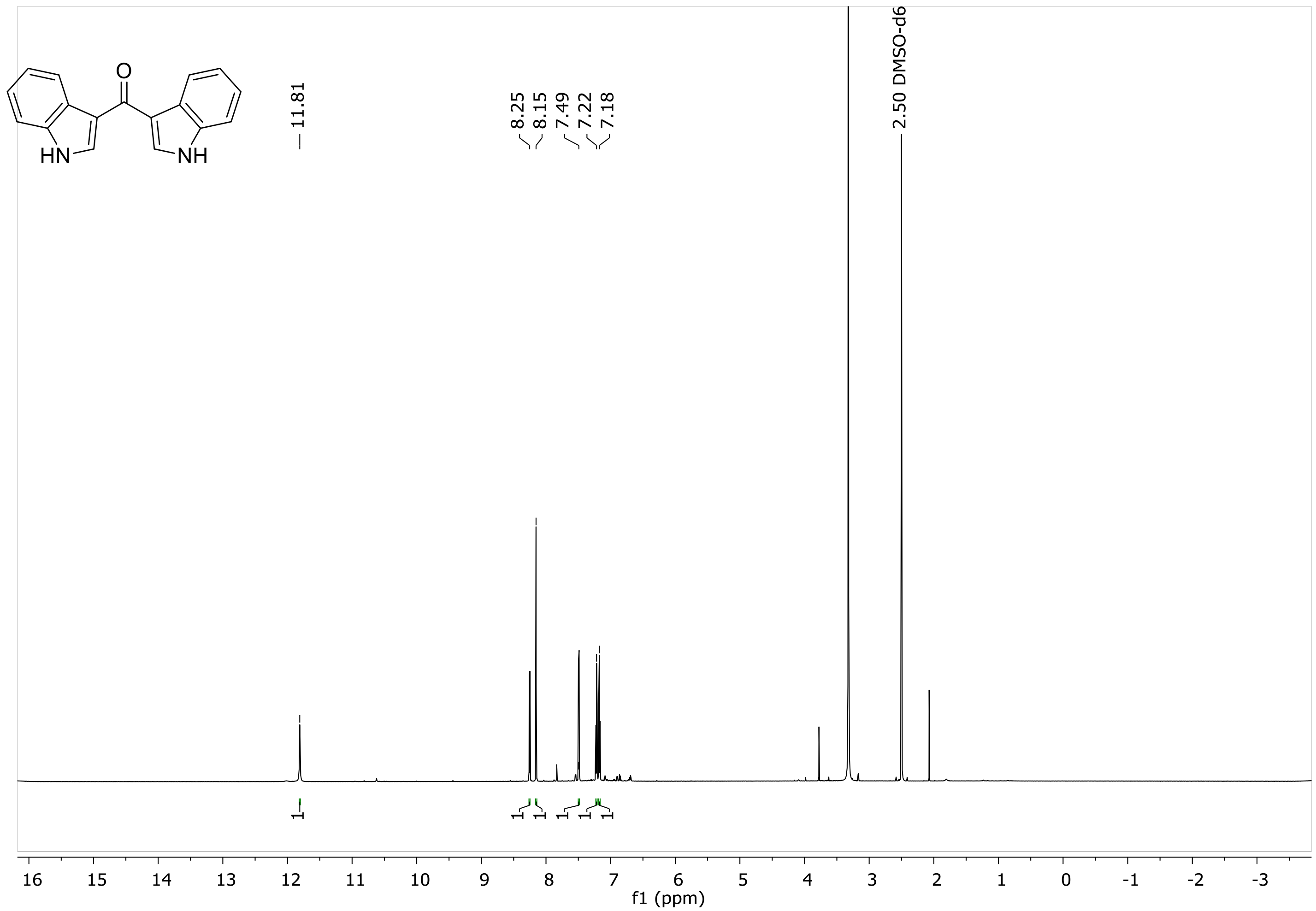

Figure S39. ^{1}H NMR spectrum (500 MHz) of di(1*H* -indol-3-yl)methanone (**21**) in DMSO-d_6

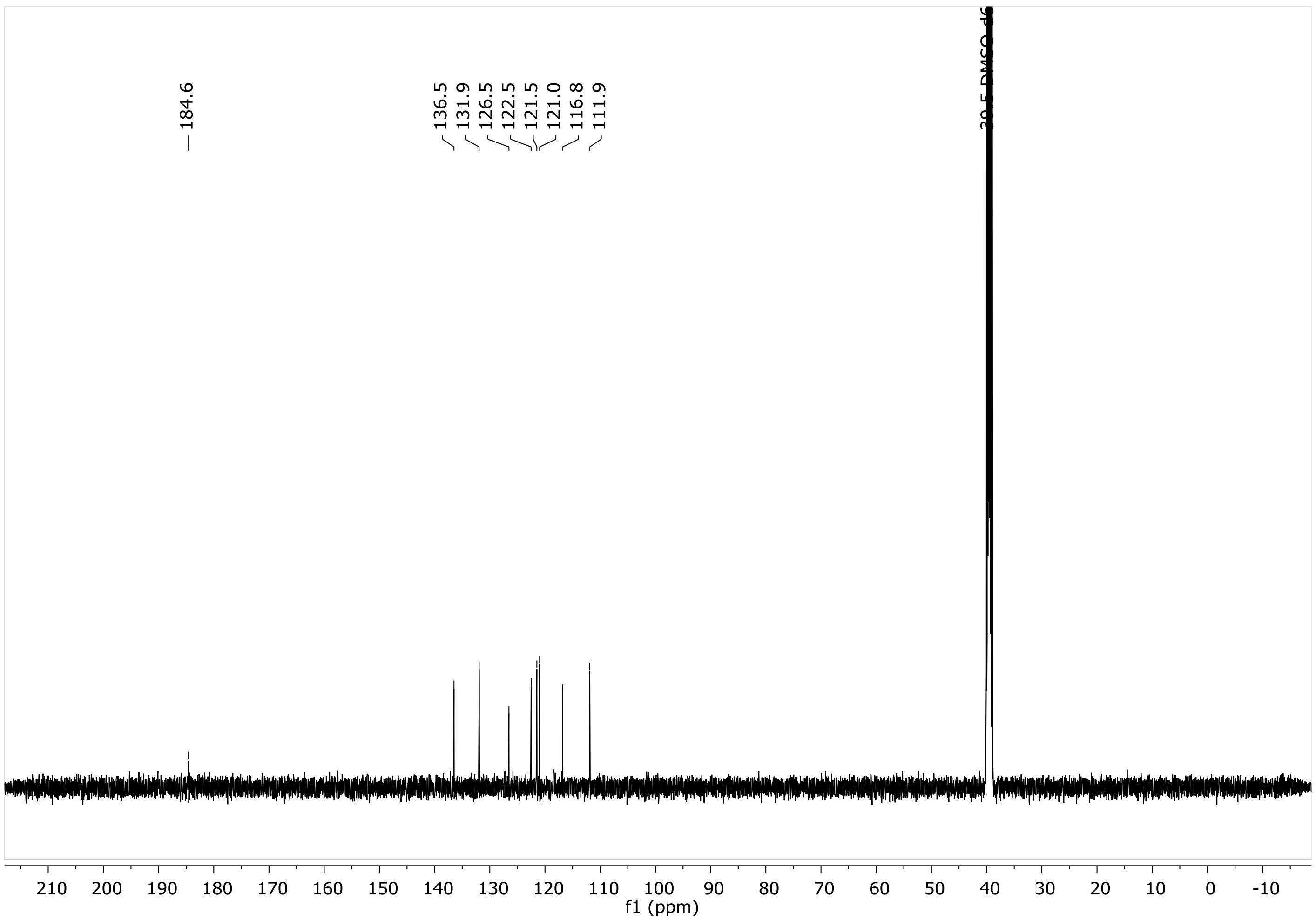

Figure S40. ^{13}C NMR spectrum (125 MHz) of di(1*H* -indol-3-yl)methanone (**21**) in DMSO-d_6

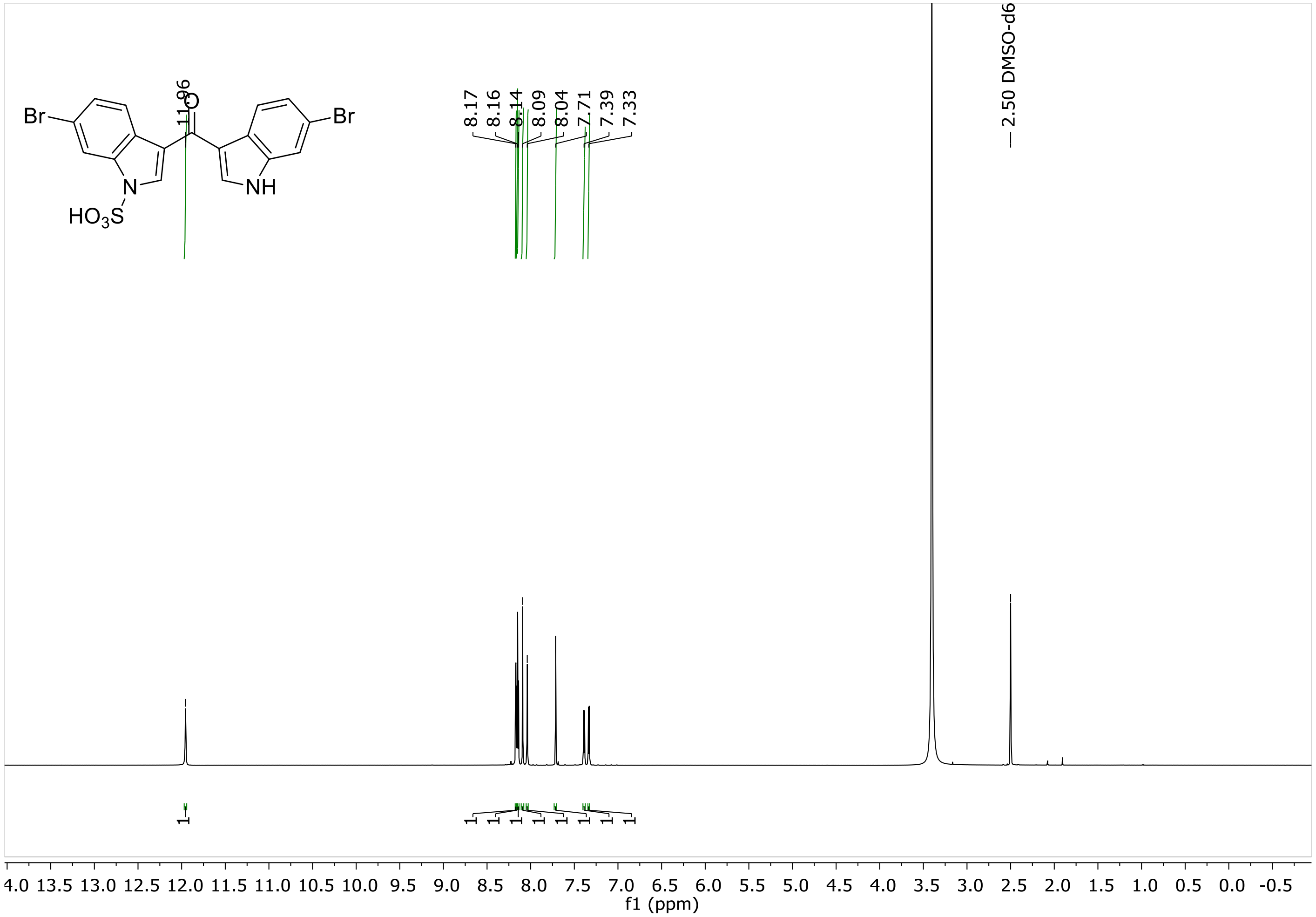

Figure S41. ^{1}H NMR spectrum (800 MHz) of synthetic echinosulfone A (**1**) in DMSO-d_6

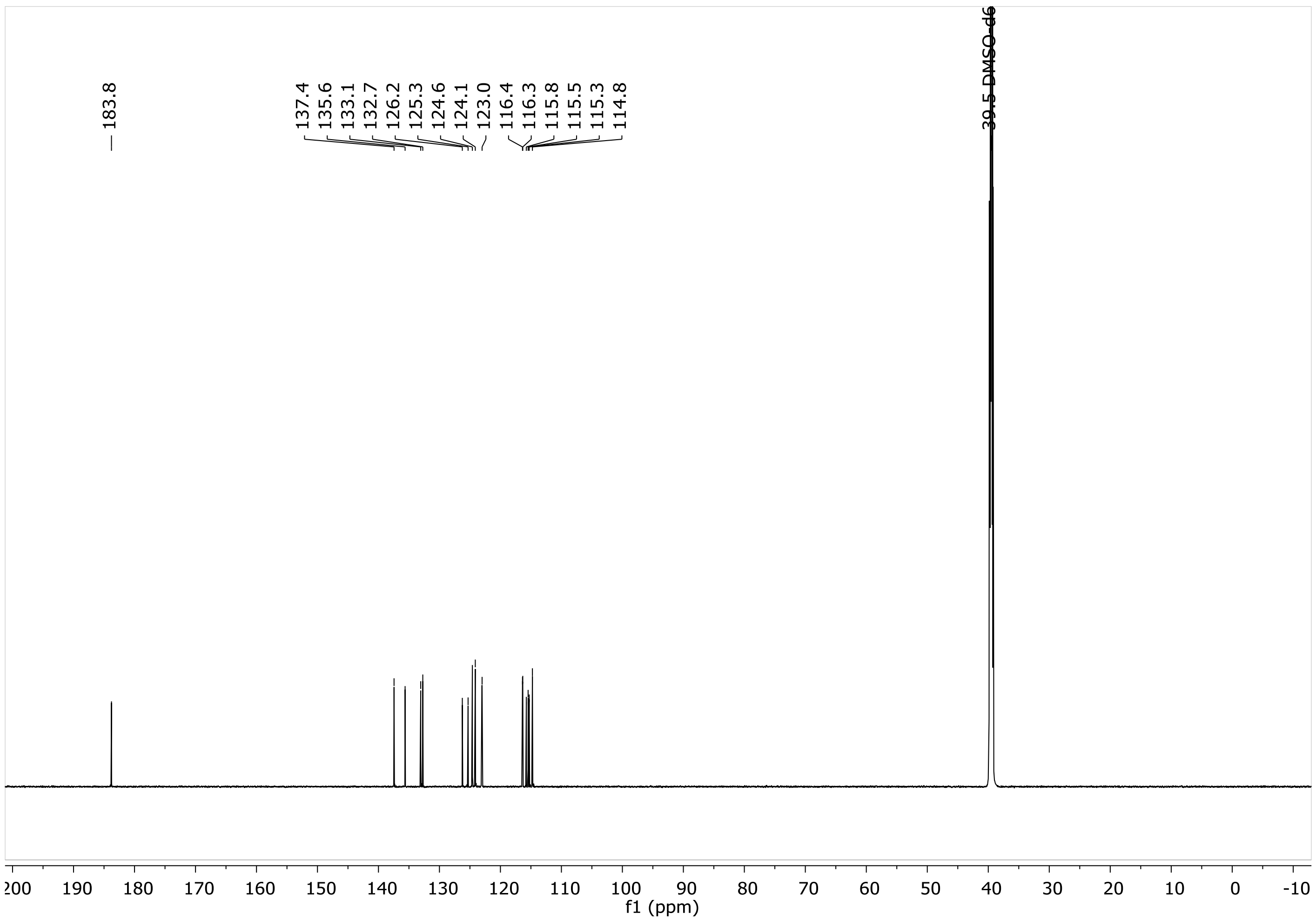

Figure S42. ^{13}C NMR spectrum (200 MHz) of echinosulfone A (**1**) in DMSO-d_6

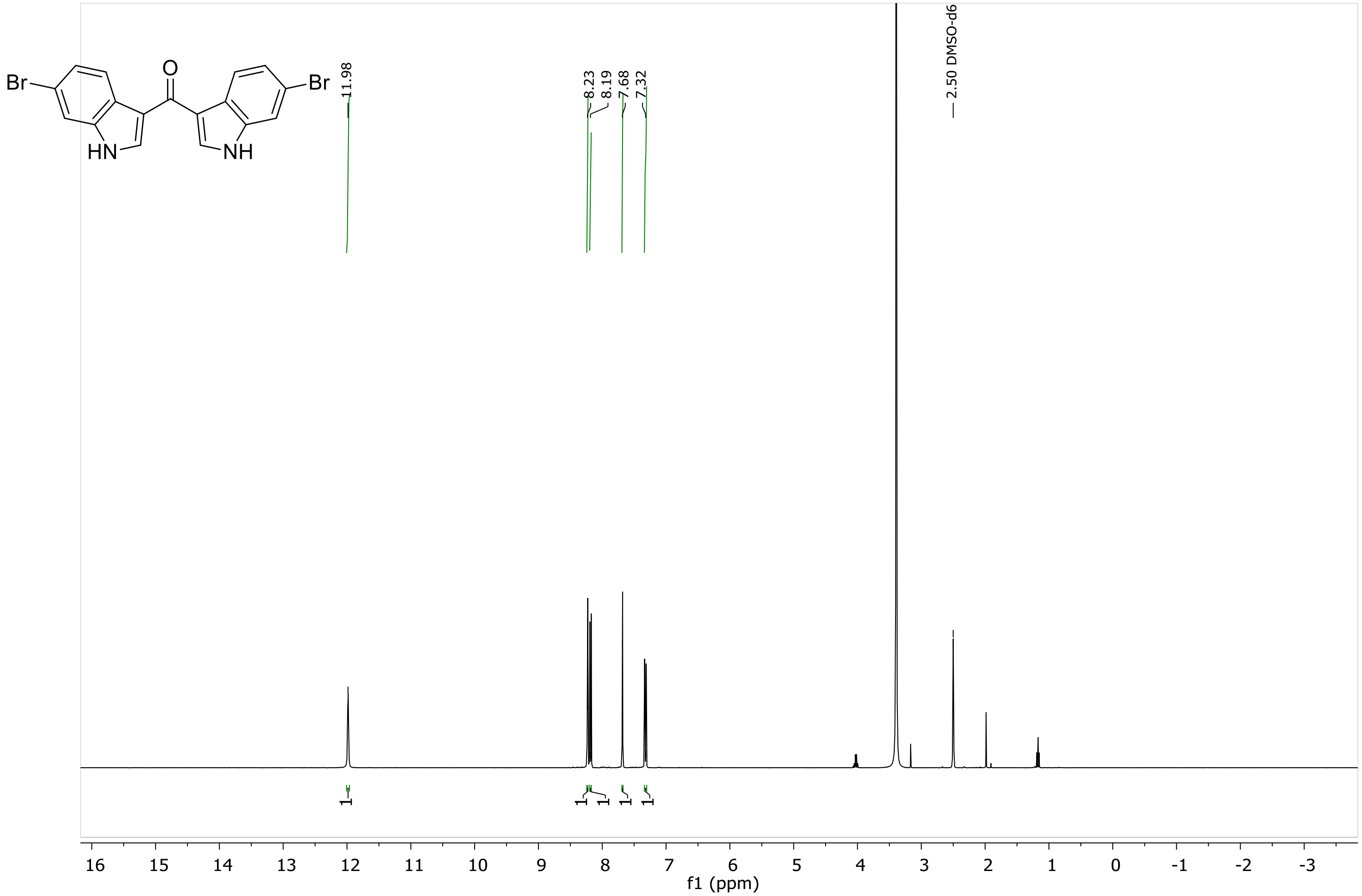

Figure S43. ^{1}H NMR spectrum (500 MHz) of bis(6-bromo-1*H* -indol-3-yl)methanone (**22**) in DMSO-d_6

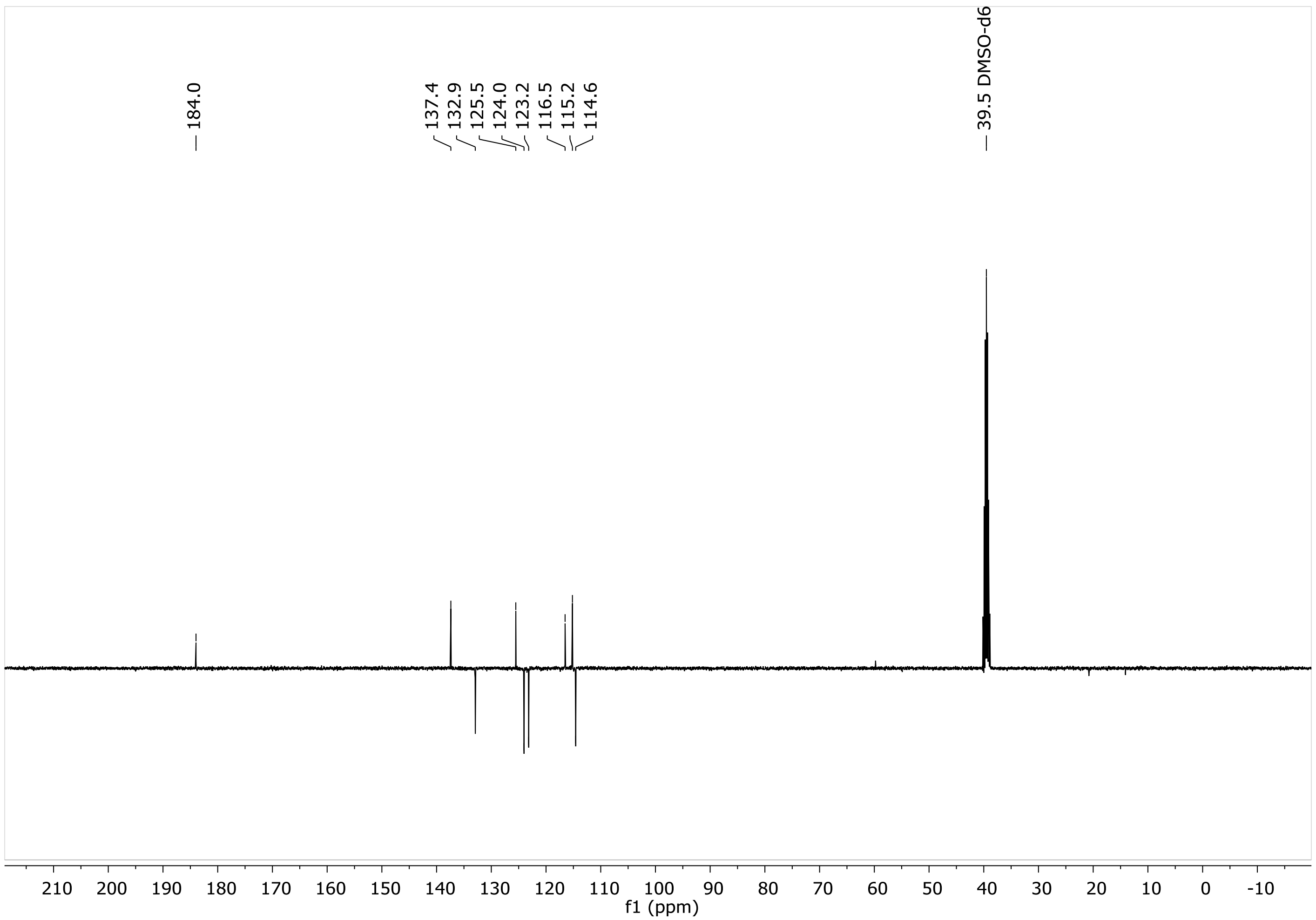

Figure S44. ^{13}C NMR spectrum (125 MHz) of echinosulfone A (**1**) bis(6-bromo-1*H* -indol-3-yl)methanone (**22**) in DMSO-d_6

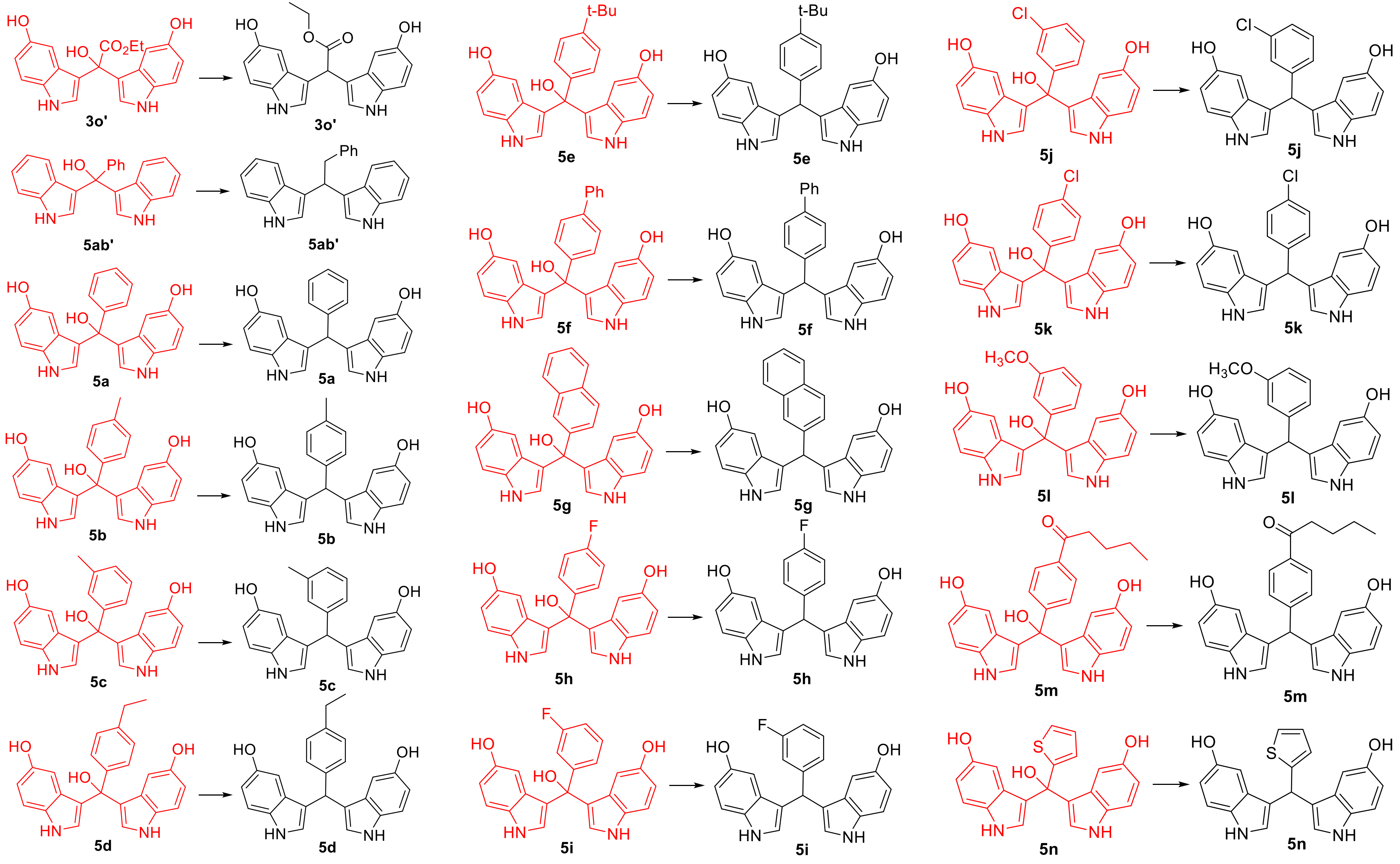

Figure S45. Incorrectly assigned synthetic α-hydroxy bis-indoles (red) and revised structures (black) for **3o'**, **5ab'**, and **5a-n**.[1]

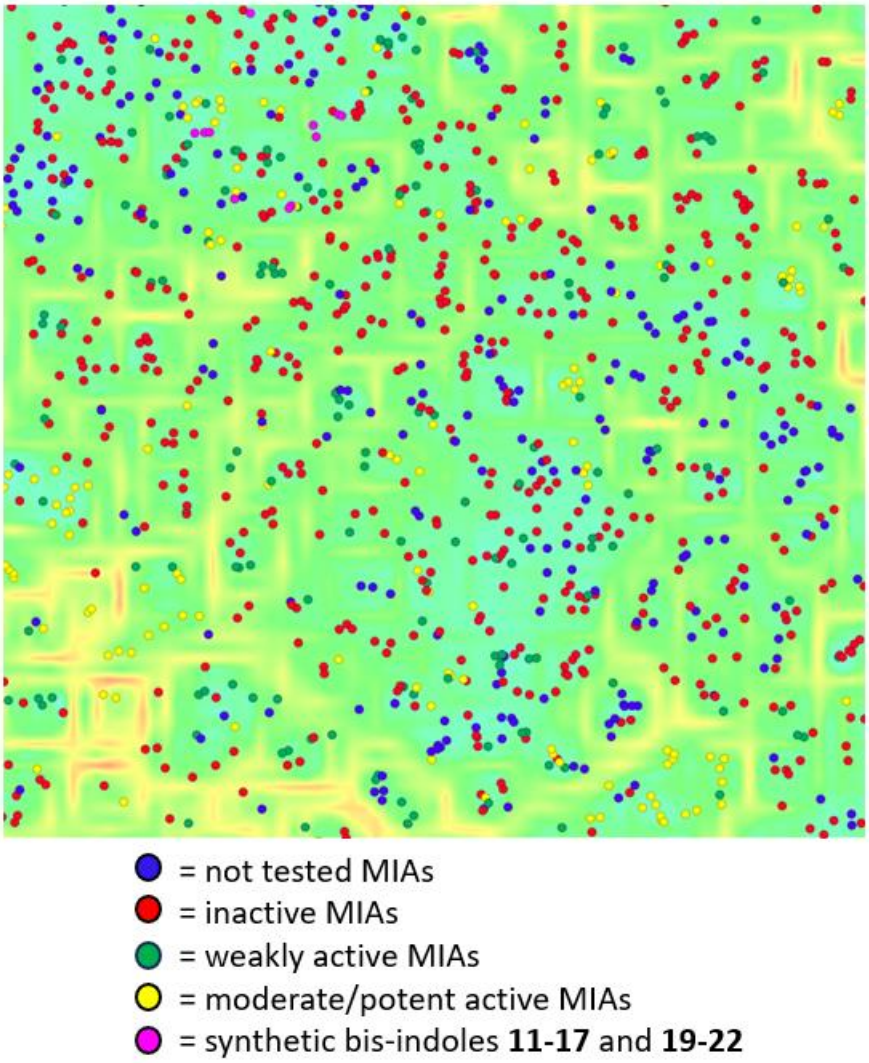

Figure S46. Chemical diversity of marine indole alkaloid (*n* = 2048) integrated with synthetic bis-indoles **11-17** and **19-22** visualized as 50 x 50 self-organizing map (SOM) using the Skelspheres 1024-bit chemical fingerprint descriptor. The reported biological activities for marine indole alkaloids (Holland and Carroll)[2] are coloured according to potency outlined in Table S1.[2,3]

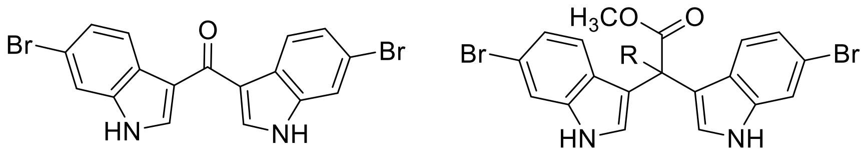

22

3a R = OCH_3

Figure S47. Desulfonation products of echinosulfone A (**22**) and echinosulfonic acid B (**3a**) from Sala et al.[4]

Table S1. Bioactivity classifications used for cheminformatic analysis of marine indole alkaloids (adapted from Holland and Carroll,[2] and Carroll et al.)[3]

Bioactivity criteria	IC_{50}
**Cytotoxic/antiparasitic/antioxidant/anti-inflammatory/antiviral/enzyme inhibitory***	
**Potent**	<0.1 µM
**Moderate**	0.1–1.0 µM
**Weak**	1.0–10 µM
**Inactive**	>10 µM
**Antibacterial/Antifungal Activity**	
	MIC
**Potent**	<1.0 µg/mL
**Moderate**	1.0–8.0 µg/mL
**Weak**	8.0–32.0 µg/mL
**Inactive**	>32.0 µg/mL

References.

(1) Li, Q.; Liang, X. X.; Zhang, W.; Han, M. Y. Friedel–Crafts Reaction of Acylsilanes: Highly Chemoselective Synthesis of 1-Hydroxy-Bis(Indolyl)Methanes and 1-Silyl-Bis(Indolyl)Methanes Derivatives. *Molecules* **2023**, *28* (15). https://doi.org/10.3390/molecules28155685.

(2) Holland, D. C.; Carroll, A. R. Marine Indole Alkaloid Diversity and Bioactivity. What Do We Know and What Are We Missing? *Natural Product Reports*. Royal Society of Chemistry February 15, 2023. https://doi.org/10.1039/d2np00085g.

(3) Carroll, A. R.; Copp, B. R.; Davis, R. A.; Keyzers, R. A.; Prinsep, M. R. Marine Natural Products. *Nat Prod Rep* **2022**, *39* (6), 1111–1368. https://doi.org/10.1039/d1np00076d.

(4) Sala, S.; Nealon, G. L.; Sobolev, A. N.; Fromont, J.; Gomez, O.; Flematti, G. R. Structure Reassignment of Echinosulfone A and the Echinosulfonic Acids A − D Supported by Single-Crystal X - Ray Di Ff Raction and Density Functional Theory Analysis. *J Nat Prod* **2020**. https://doi.org/10.1021/acs.jnatprod.9b00902.
